# Acid-Catalyzed Dehydrative Nucleophilic Substitutions of 2,6-Di(hydroxymethyl) BODIPYs: A Platform for BODIPY Functionalization

**DOI:** 10.1021/acs.orglett.5c04625

**Published:** 2025-12-08

**Authors:** Clara Uriel, Alberto Hernández, Natalia Casado, Jorge Bañuelos, Eduardo Duque-Redondo, Lourdes Infantes, Inmaculada García-Moreno, Ana M. Gómez, J. Cristóbal López

**Affiliations:** † Instituto de Química Orgánica General (IQOG), 201430 Consejo Superior de Investigaciones Científicas (CSIC), Juan de la Cierva 3, 28006 Madrid, Spain; ‡ Departamento de Química Física. 16402 Universidad del País Vasco (EHU), Apartado 644, 48080 Bilbao, Spain; § Instituto de Química-Física “Blas Cabrera” (IQF), Consejo Superior de Investigaciones Científicas (CSIC), Serrano 119, 28006 Madrid, Spain

## Abstract

We report the synthesis of the novel 2,6-di­(hydroxymethyl)-1,3,5,7-tetramethyl-8-phenyl BODIPY and its utility in efficient Lewis-acid-catalyzed dehydrative nucleophilic substitution reactions. This diol, readily prepared via a formylation/reduction sequence from the corresponding tetramethyl BODIPY, undergoes bisfunctionalization with a variety of nucleophiles to yield 2,6-substituted BODIPY fluorophores. Notably, the use of trimethylsilyl azide as a nucleophile provides a “clickable” BODIPY, suited for bioconjugation. Furthermore, employing BODIPY or BOPHY as a nucleophile successfully led to a BODIPY trimer and the first example of bis­(BOPHY)–BODIPY, respectively, with the latter showcasing its potential as a novel light-harvesting fluorophore.

## Graphical abstract

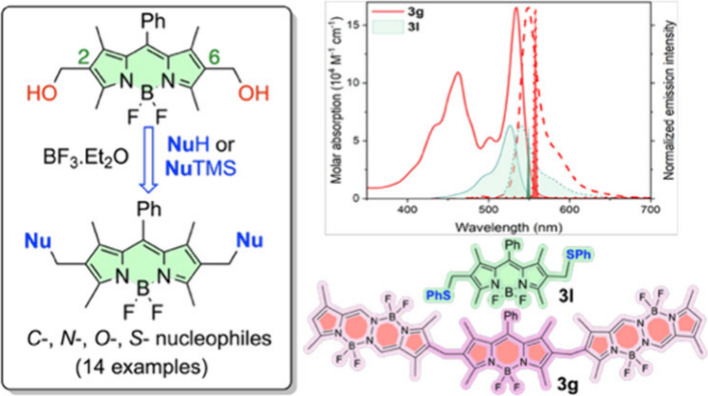

BODIPY (4,4′-difluoro-4-bora-3a,4a-diaza-*s*-indacene) dyes, e.g., **1** (Figure 1),^1^ are renowned small-molecule fluorophores. This prominence stems from their exceptional photophysical properties, including high fluorescence quantum yields, remarkable photostability, and large molar absorptivity. Consequently, BODIPYs have found extensive applications in diverse fields, from bioimaging,^2^ sensing,^3^ and drug delivery^4^ to materials science.^5^ Specific examples include their use in photodynamic therapy,^6^ biomolecule labeling,^7^ tunable laser dyes,^8^ organic photovoltaics, and photosensitizers and as components in organic light-emitting diodes (OLEDs)^9^ and light-harvesting systems.^10^ Perhaps their greatest advantage lies in their chemical flexibility and stability, which makes them the fluorophores of choice for many applications. This inherent adaptability allows for post-functionalization^11^ to fine-tune their photophysical, physical, and chemical properties.^12^ For instance, planned functionalization of the BODIPY skeleton can modulate photophysical characteristics,^13^ induce bathochromic^14^ or hypsochromic^15^ shifts in absorption and emission bands,^16^ and enhance photostability.^17^ Furthermore, post-functionalization facilitates the attachment of other (bio)­molecules and can address issues, such as (water) solubility^18^ and aggregation.^19^

**Figure 1:**
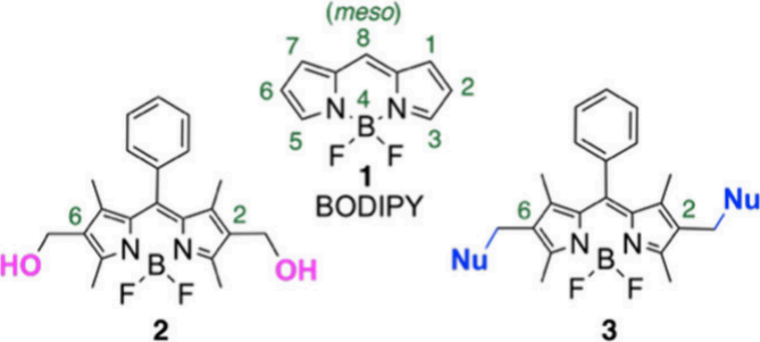
BODIPY **1**, 2,6-di­(hydroxymethyl) BODIPY **2**, and 2,6-bis-functionalized BODIPY **3**.

In this letter, we report the Lewis-acid-catalyzed dehydrative substitution reactions^20^ of the previously undescribed 2,6-di­(hydroxymethyl) BODIPY **2** (Figure 1).^21^ This, atom-economical,^22^ method provides access to a variety of 2,6-bis-functionalized BODIPY derivatives, **3** (Figure 1).

These investigations originated from our interest in the synthesis of BODIPY–carbohydrate conjugates.^23^ We attempted the glycosylation of BODIPY diol **2**,^24^ with a highly reactive d-glucopyranosyl trichloroacetimidate donor **4**,^25^ to gain access to bis-glycosylated BODIPY **5**. However, treating compounds **2** and **4** with BF_3_·OEt_2_ in CH_2_Cl_2_ at −78 °C (conditions known to activate glycosyl donor **4**) resulted in extensive decomposition and polymerization of BODIPY **2** (Scheme 1).^26^ Suspecting an acid-mediated BODIPY–carbocation formation, we introduced allyltrimethylsilane to trap the reactive intermediates. This successfully yielded **3a** (Scheme 1), confirming our mechanistic hypothesis (Scheme S4 of the Supporting Information).

**Scheme 1:**
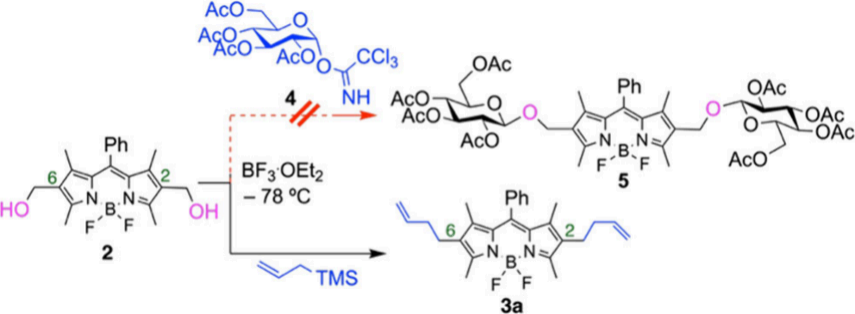
Attempted Glycosylation of BODIPY Diol **2** with **4** and Acid-Catalyzed Allylation of **2** Leading to BODIPY **3a**

To evaluate the scope of this transformation, we tested a series of diverse (C-, S-, H-, N-, and O-) nucleophiles, and the results are summarized in Scheme 2. Beyond the reaction of BODIPY diol **2** with allyltrimethylsilane affording **3a** (Schemes 1 and 2), we also employed trimethylsilyl cyanide to yield bis-cyanide **3b**. Similarly, the reaction of **2** with pentane-2,4-dione furnished the carbonyl derivative **3c**. When indole was used as the nucleophile, bis­(3-indolyl) BODIPY **3d** was obtained. The reaction of BODIPY **2** with *p*-methylphenol yielded bis-phenol derivative **3e**, consistent with a Friedel–Crafts alkylation occurring *ortho* to the phenol group. Notably, 1,3,5,7-tetramethyl-8-phenyl BODIPY and 1,3,6,8-tetramethyl BOPHY could also serve as nucleophilic partners, producing BODIPY trimer **3f** and 2,6-bis­(BOPHY)-BODIPY **3g**, respectively.

**Scheme 2:**
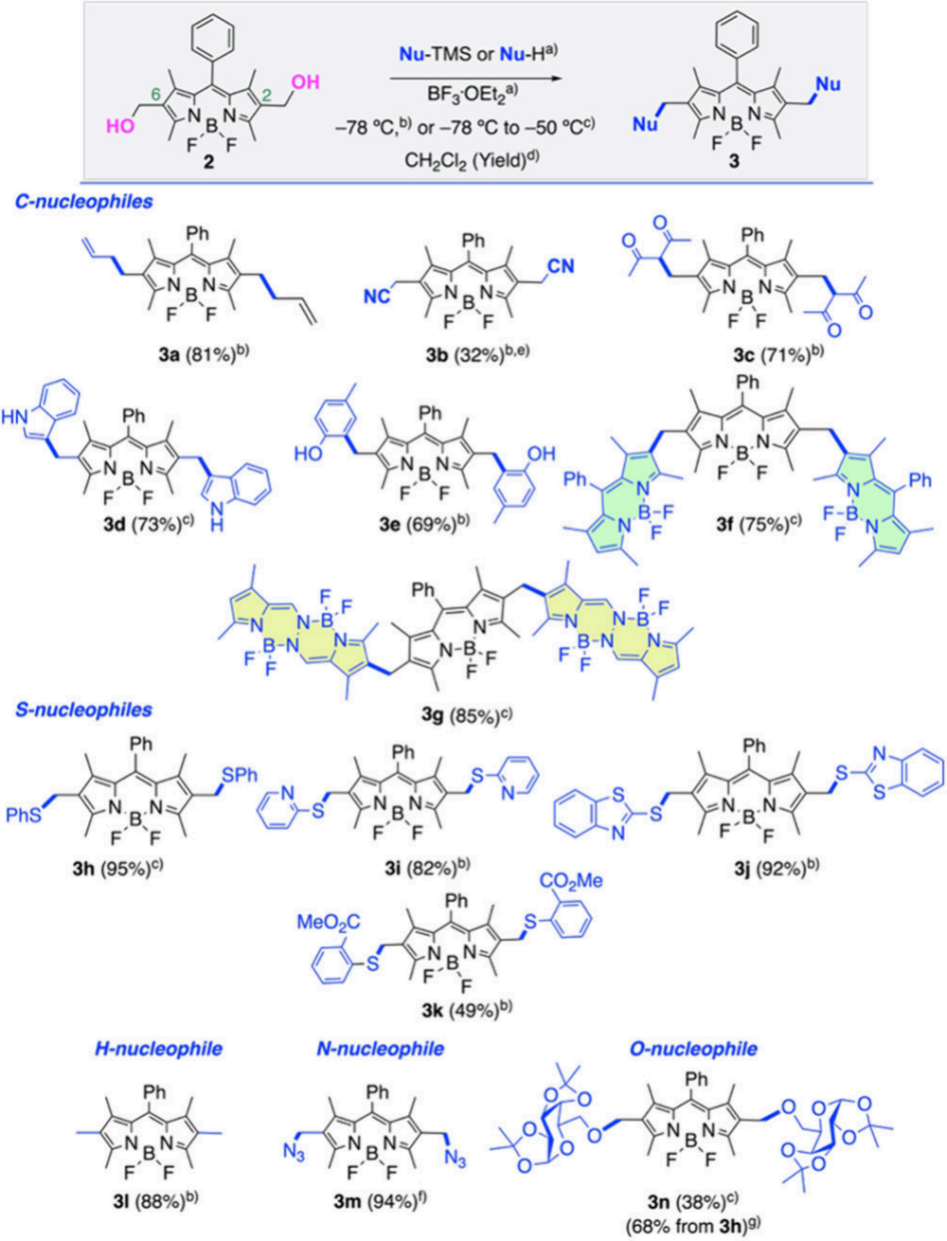
Synthesis of 2,6-Bis-Functionalized BODIPYs **3a**–**3n** by Reaction of BODIPY Diol **2** with C-, S-, H-, N-, and O-Nucleophiles

a All reactions were carried out in CH_2_Cl_2_, using 0.5 equiv of BF_3_.OEt_2_ and 1.5 or 2.0 equiv of nucleophile per OH group.
b At −78 °C.
c At −78 °C for 30 min and then −50 °C.
d Isolated yields.
e The corresponding B­(F,CN)-BODIPY was also isolated (54%).
f The reaction was run at 0 °C for 90 min.
g
**3n** (68%) was obtained from BODIPY **3h** (NIS and CH_2_Cl_2_ at −60 °C for 60 min).

Thiol nucleophiles also coupled efficiently with BODIPY diol **2**, providing good to excellent yields of bis-thioethers **3h**, **3i**, **3j**, and **3k**. The utilization of Et_3_SiH as a nucleophile enabled the formation of permethylated BODIPY **3l**. Furthermore, trimethylsilyl azide proved effective as a nucleophilic partner, leading to clickable BODIPY **3m**. The synthesis of **3m** could be carried out on a gram scale, and the corresponding product was isolated in 90% yield.

Finally, to evaluate the viability of alcohols as nucleophiles in dehydrative coupling, we tested commercially available galactose diacetonide. The direct reaction of BODIPY diol **2** with galactose diacetonide led to the formation of bis-saccharidic derivative **3n**, albeit in a relatively low yield (38%), likely due to potential activation of the ether functionalities of **3n** under the acidic reaction conditions. Nevertheless, we successfully achieved higher yields of **3n** (68%) under neutral conditions via the NIS-catalyzed reaction of bis-thioether **3h** with galactose diacetonide.

Bis-azidomethyl BODIPY **3m** proved to be an effective substrate for the copper­(I)-catalyzed azide–alkyne cycloaddition (CuAAC) click reaction.^27,28^ Thus, treatment of **3m** with 3-phenyl-1-propyne and propargyl 2,3,4,6-tetra-*O*-acetyl-α-d-mannopyranoside resulted in the formation of the bis-triazolyl derivatives **7a** and **7b** (Scheme 3). Our primary goal was to compare the properties of these new derivatives to their readily accessible isomeric counterparts, **9a** and **9b** (obtained from bis-propargyl derivative **8**),^29^ which are known for their J-aggregation-induced emission (Scheme 3).^30^ We anticipated that exploring the J-aggregation properties of **7a** and **7b** would provide valuable structural insights.

**Scheme 3:**
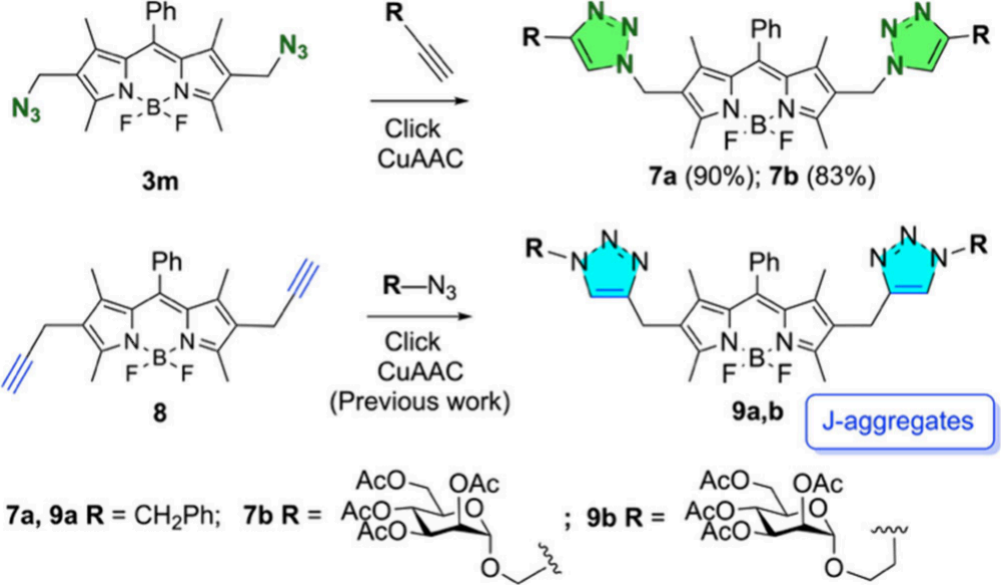
Click CuAAC Reactions of BODIPY **3m** Leading to BODIPY Derivatives **7a** and **7b** and Previous Work: Click CuAAC Reaction of BODIPY **8** Leading to Isomeric Bis-triazolyl BODIPYs **9a** and **9b** ^29,30^

In addition, having “complementary” bis-azidomethyl BODIPY **3m** and bis-propargyl BODIPY **8** in hand, we decided to test the viability of a “bis-click” approach to a BODIPY-based macrocycle.^31^ The bis-cyclization took place smoothly and provided cyclic derivative **10**, which showed poor solubility in common solvents (Scheme 4).

**Scheme 4:**
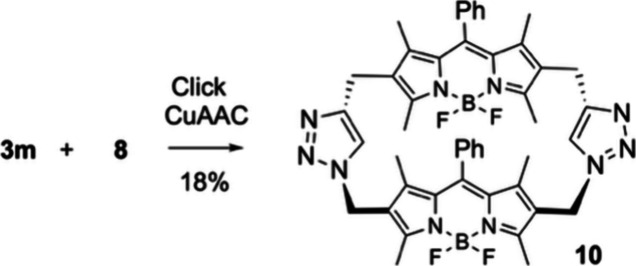
CuAAC-Mediated “Click” Macrocyclization of **3m** and **8**

The incorporation of C- (**3c**), S- (**3h**), N- (**3m**), and O- (**3n**) nucleophiles at the 2,6-methyl groups minimally affected the BODIPY photophysical behavior compared to permethylated BODIPY **3l** (Table 1). The methylene spacer prevented significant electronic resonance, leading to the expected absorption and fluorescence profiles. While the more electronegative N- and O-nucleophiles (**3m** and **3n**) caused hypsochromic shifts (≈10 nm absorption and up to 20 nm emission; Figure S1 of the Supporting Information), all derivatives exhibited strong absorption (ε > 60 000 M^–1^ cm^–1^) and bright fluorophores (Φ_F_ = 60–75%; Table 1). Furthermore, they displayed highly efficient laser emission (with yields up to 50%) centered near 550 nm, largely independent of the incorporated nucleophile (Table 1).

**Table 1:** Photophysical^a^ and Lasing^b^ Properties of Representative Functionalized BODIPYs, with Additional Photophysical Data Collected in Tables S1–S3 of the Supporting Information

	λ_ab_ ^c^ (nm)	ε_max_ ^d^ (M^–1^ cm^–1^)	λ_fl_ ^e^ (nm)	ϕ^f^	λ_la_ ^g^ (nm)	% eff^h^
**3l**	526.5	63000	541.5	0.73	548.0	48
**3c**	522.0	88000	535.0	0.72	550.0	47
**3h**	525.0	86000	543.0	0.68	552.0	50
**3m**	511.0	100000	523.0	0.70	548.0	46
**3n**	511.0	56000	522.0	0.76	547.0	49
**7a** ^i^	506.5	76000	518.0	0.67	546.0	43
**9a** ^i^	519.0	65000	532.0	0.79	548.0	50
					562.0	
**3f**	547.5	159000	560.0	0.77		
**3g**	533.0	172000	546.0	0.76	557.0	53
	461.0	117000				
**10** ^j^	522.0	14500	532.0	0.03		
	510.0	13500				

a Dye concentration = 2 μM.

b Dye concentration in the millimolar range was selected to optimize its laser efficiency.

c Absorption.

d Fluorescence wavelength.

e Molar absorption coefficient.

f Fluorescence quantum yield.

g Laser wavelength.

h Efficiency.

i Data in chloroform, except ethyl acetate.

j Data in chloroform, except DMSO.

As previously mentioned, we reported that 2,6-bis­((1-benzyl-1*H*-1,2,3-triazol-4-yl)­methyl)-BODIPY **9a** promotes spontaneous J-aggregation.^30^ In contrast, isomeric (4-benzyl-1*H*-1,2,3-triazol-1-yl)­methyl-BODIPY **7a** showed no spectroscopic evidence of J-aggregation. The latter exhibited laser emission at ca. 545 nm in ethyl acetate with an efficiency of 43% (Table 1 and Figure 2), and its emission profile remained single-peaked and monotonic with respect to the dye concentration and pump energy, lacking the bichromatic features typical of J-aggregates (Figure S2 of the Supporting Information). This remarkable difference highlights the critical role of the linkage site within the triazole moiety in driving supramolecular behavior.

**Figure 2:**
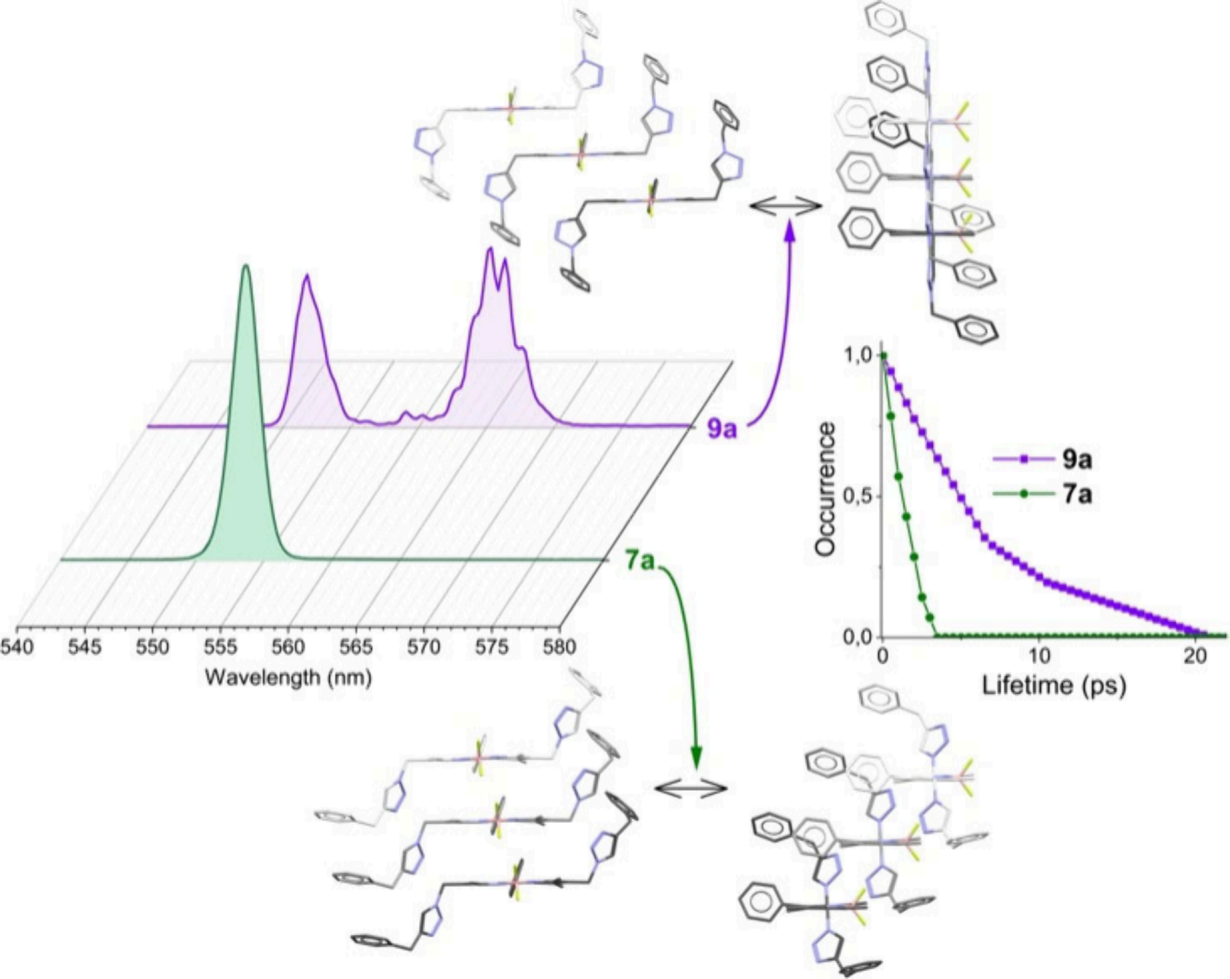
Laser emission of both BODIPY isomers at a dye concentration of 2 mM and pump energy of 5 mJ. The corresponding X-ray diffraction crystalline packing (in two views) and mean lifetime of the intermolecular encounters during the simulated trajectories in a solvent cage are also provided. Detailed crystalline packing and geometrical data can be found in Figure S5 and Table S4 of the Supporting Information, respectively.

Although the substitution pattern in **7a** induced a hypsochromic shift of ≈15 nm in both absorption and emission bands relative to **9a** (Table 1), the overall intrinsic photophysical properties of the BODIPY core remained intact (Figure S3 of the Supporting Information). The observed shifts were attributed to differences in electronegativity between N- and C-based substituents.

To elucidate the molecular origin of the divergent aggregation behavior between **7a** and **9a**, we conducted a comparative analysis of their intermolecular interaction landscapes through all-atom molecular dynamics (MD) simulations in explicit solvent, supplemented by high-resolution single-crystal X-ray diffraction. The combined computational and crystallographic data unveiled markedly distinct supramolecular organization and interaction motifs for each dye under identical thermodynamic conditions. MD trajectories revealed persistent intermolecular contacts in **9a**, with a mean encounter lifetime of approximately 5 ps (Figure 2 and Video S1 in the Supporting Information). Conversely, dye **7a** showed fewer and more transient interactions, with average contact durations of only ≈1.25 ps (Figure 2 and Video S2 in the Supporting Information), indicating a pronounced preference for remaining solvated (Figure S4 of the Supporting Information). Crystallographic data further corroborated these findings: **9a** adopted an extended “slipped-stack” geometry aligned along the transition dipole axis of the BODIPY core, forming a canonical “head-to-tail” arrangement characteristic of J-aggregates (Figure 2 and Figure S5 of the Supporting Information). In contrast, **7a** exhibited a “face-to-face” stacking motif, more reminiscent of H-type aggregates, with lateral offset along the transverse molecular axis (Figure 2 and Figure S5 of the Supporting Information). This geometry precludes the coherent excitonic coupling necessary for J-aggregation-induced laser emission. The differing self-assembly behaviors of these isomers may stem from the coordination behavior of triazole nitrogen. In **7a**, one of the triazole nitrogen atoms engages intramolecularly with the 2,6-methyl groups of the BODIPY core, reducing its availability for intermolecular interactions. In **9a**, in contrast, the corresponding lone pair remains uncoordinated, enabling favorable triazole–triazole interactions that facilitate ordered aggregation. These findings underscore the importance of triazole connectivity in dictating the self-assembly pathways and emissive properties of BODIPY derivatives.

All-BODIPY-based trimer **3f** displayed red-shifted strong absorption bands (Table 1), arising from the additive electronic contribution of each chromophore to the overall transition (Figure S6 of the Supporting Information). Upon excitation, **3f** emitted bright fluorescence (Figure S6 of the Supporting Information), with a quantum yield similar to that of monomeric derivatives (Table 1). However, this emission became significantly quenched in a highly polar solvent (Table S3 of the Supporting Information), owing to the activation of photoinduced electron transfer pathways, a well-established process in BODIPY-based oligomers.^32^ The limited solubility of **3f** precluded achieving the optical density required to evaluate its laser activity under the selected experimental conditions. In contrast, substitution of the peripheral BODIPY units with BOPHY chromophores in **3g** resulted in a fully soluble system, allowing for comprehensive photophysical and laser characterization. DFT calculations (Figure S7 of the Supporting Information) indicated that the methylene bridges electronically decoupled the chromophoric units, preserving their individual absorption profiles. This modular arrangement yielded a characteristic broadband absorption spectrum with contributions from both BOPHY (ca. 460 nm) and BODIPY (ca. 530 nm) components (Figure 3).

**Figure 3:**
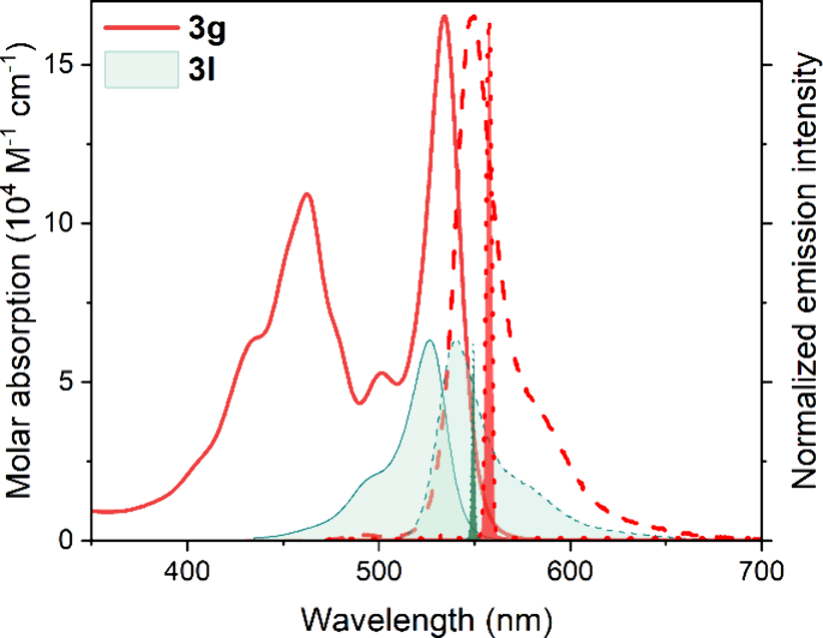
Absorption and normalized fluorescence (dashed) and laser spectra (dotted) of BODIPY–BOPHY triad **3g** in diluted solution of toluene. For comparison, the corresponding spectra of single BODIPY **3l** (green shaded) are also included.

Regardless of the excitation wavelength, **3g** displayed strong fluorescence (ϕ ∼ 80%; Table 1) and laser (% eff ∼ 53%; Table 1) signals, with emission centered at ∼545 and ∼557 nm, respectively, indicating efficient intramolecular excitation energy transfer (EET) from the BOPHY donors to the central BODIPY unit acting as an energy acceptor and emissive center (Figure 3). Evidence for efficient Förster-type EET included the residual BOPHY fluorescence under its selective excitation, short donor–acceptor distances enforced by the methylene linkers, and substantial spectral overlap between BOPHY emission and BODIPY absorption. As anticipated, polar solvents, such as acetonitrile, again reduced the fluorescence quantum yield due to photoinduced electron transfer (Table S3 of the Supporting Information).

Finally, compound **10**, which was sparingly soluble in most solvents, could be only solubilized in DMSO. Its absorption spectrum featured a broad, flattened profile with a prominent high-energy shoulder adjacent to the main peak (Figure S8 of the Supporting Information) and a notably reduced molar absorptivity (<20 000 M^–1^ cm^–1^; Table 1), despite comprising two covalently linked BODIPY units. Fluorescence was weak (ϕ ≈ 3%; Table 1), with a maximum centered at ca. 532 nm. These characteristics pointed to the formation of non-emissive H-type aggregates. Ground-state geometry optimization (CAM-B3LYP/6-311G*) confirmed a face-to-face, parallel orientation of the BODIPY units upon cyclization (Figure S8 of the Supporting Information), a geometry conducive to excitonic coupling and photoluminescence quenching typical of H-aggregates.

In summary, we report the preparation and synthetic utility of the novel 2,6-di­(hydroxymethyl)-1,3,5,7-tetramethyl-8-phenyl BODIPY. This diol, conveniently accessed through a sequential Vilsmeier–Haack formylation and reduction protocol, undergoes dehydrative nucleophilic substitution reactions, which provide an efficient approach for C–C, C–S, C–O, and C–N bond formation. C–C bond-forming reactions include Friedel–Crafts alkylations, enabling access to trimeric BODIPY species and a bis­(BOPHY)–BODIPY derivative. C–S bond formation yields BODIPY thioethers, which upon activation under neutral conditions can be coupled to hydroxyl compounds providing BODIPY ether derivatives. Finally, the use of azide as a nucleophile leads to clickable bis-azidomethyl BODIPY that undergoes efficient CuAAC coupling reactions. This broadly applicable synthetic strategy provides tailored BODIPY-based fluorophores suitable for tunable lasers, aggregation-induced emission (AIE), and light-harvesting applications.

## Supplementary Material



## Data Availability

The data underlying this study are available in the published article and its Supporting Information.

## Notes

Experimental and computational procedures, copies of NMR spectra, photophysical data, absorption and fluorescence spectra, simulated absorption spectra, and molecular orbitals (PDF)
