# Supplementary material for: Acid-Catalyzed Dehydrative Nucleophilic Substitutions of 2,6-Di(hydroxymethyl) BODIPYs: A Platform for BODIPY Functionalization
Source: Org Lett. 2025 Dec 8;27(50):14052–7. doi: 10.1021/acs.orglett.5c04625 (PMC12723669; doi:10.1021/acs.orglett.5c04625)
Supplement: Supplementary file 1 [file ol5c04625_si_001.pdf]

Supporting Information

Acid-Catalyzed Dehydrative Nucleophilic Substitutions of 2,6-Di-(hydroxymethyl) BODIPYs: a Platform for BODIPY Functionalization

Clara Uriel,[$] Alberto Hernández,[$] Natalia Casado,[§] Jorge Bañuelos,[§]* Eduardo Duque-Redondo,[§] Lourdes Infantes,[¶] Inmaculada García-Moreno,[¶] Ana M. Gómez,[$]* J. Cristóbal López,[$]*

[$] *Instituto de Química Orgánica General,* IQOG-CSIC*, Juan de la Cierva 3, 28006, Madrid, Spain.*
[§] *Departamento de Química Física. Universidad del País Vasco-EHU, Apartado 644, 48080, Bilbao, Spain.*
[¶] *Instituto de Química-Física "Blas Cabrera",* IQF-CSIC*, Serrano 119, 28006, Madrid, Spain*

Table of contents

1. General information 2
2. General procedures 5
3. Synthetic route to diol 2 6
4. Synthetic procedures and compound characterization 6
5. Click CuAAC reactions of bis-azidomethyl BODIPY 3l. 15
6. Plausible mechanism for the formation of compounds 3a and 3l 16
7. Tables S1-S3. Photophysical data 17
8. Figures S1-S3. Absorption, fluorescence and laser spectra 18
9. Figure S4 and Videos S1-S2. Molecular dynamics 20
10. Figure S5. X-ray crystalline molecular packing 21
11. Figures S6-S8. Quantum mechanics simulations and absorption and fluorescence spectra of trimers 21
12. Table S4 and Figure S9. X-ray diffraction data 23
13. Cartesian coordinates of optimized geometries 24
14. Copies of IR spectra of compounds 3m, 8 and 10 28
15. HRMS spectrum of compound 10 30
16. Copies of ^{1}H, ^{13}C {^{1}H}, ^{19}F, ^{11}B NMR spectra 31

1. General information

Unless otherwise stated, all reagents and solvents were of commercial quality and used without further purification. Reactions were monitored by thin-layer chromatography (TLC) on Merck Kieselgel 60 F_{254} plates, with visualization under UV light where applicable. Flash column chromatography was performed using silica gel (230–400 mesh). Optical rotations were measured on a Jasco P-2000 polarimeter. NMR spectra (^{1}H, ^{13}C, ^{11}B, and ^{19}F) were recorded on BRUKER AVANCE III HD-400, JEOL JNM-ECZ400R or VARIAN SYSTEM-500 spectrometers. Chemical shifts (δ) are reported in parts per million (ppm) relative to the residual solvent signal as internal standard. Signal multiplicities are abbreviated as follows: s = singlet, d = doublet, t = triplet, q = quartet, m = multiplet, br = broad. Coupling constants (J) are given in hertz (Hz). All ^{13}C NMR spectra were recorded with proton decoupling. High-resolution mass spectrometry (HRMS) analyses were performed using an Agilent 6500 Accurate Mass Q-TOF LC-MS system, with mass accuracy within 5 ppm. Infrared spectra were recorded in a FT-IR PerkinElmer UATR Two Spectrometer.

2-Formyl-BODIPY **S2**[1] and 2,6-diformyl-BODIPY **S3**[2] were synthesized from tetramethyl-BODIPY **S1**[3] via the Vilsmeier–Haack reaction. 8-Phenyl-1,3,5,7-tetramethyl-2,6-di(prop-2-in-1-yl)-4,4-difluoro-4-bora-3a,4a-diaza-*s*-indacene **8**, and compounds **9a,b** were prepared according to previously reported methods.[4]

Photophysical properties

The ultraviolet-visible (UV-vis) absorption and fluorescence spectra were recorded on a CARY 7000 spectrophotometer (Agilent) and an Edinburgh Instruments spectrofluorometer (model FLSP920), respectively. Dye solutions were prepared by diluting a concentrated stock solution in ethyl acetate (ca. 10^{-3} M). The optical density was adjusted to be similar regardless of the dye concentration, choosing the more adequate path length of the quartz cuvettes (1 cm for diluted solution in the micromolar scale, 0.1 cm for 10^{-5} M, 0.01 cm for 10^{-4}, and 0.001 cm for millimolar) to avoid inner filter effects. Furthermore, the fluorescence signatures of the concentrated solutions (>10^{-5} M) were recorded in the front-face

[1] (a) Jiao, L.; Yu, C.; Li, J.; Wang, Z.; Wu, M.; Hao, E. β-formyl-BODIPYs from the Vilsmeier-Haack reaction. *J. Org. Chem.* **2009**, *74*, 7525–7528. (b) Li, Y.; Wang, Y.; Li, Y.; Shi, W.; Yan, J. Construction and evaluation of near-infrared fluorescent probes for imaging lipid droplet and lysosomal viscosity. *Spectrochim. Acta A Mol. Biomol. Spectrosc.* **2024**, *316*, 124356. (c) Ventura, J.; Uriel, C.; Gómez, A. M.; Avellanal-Zaballa, E.; Bañuelos, J.; Rebollar, E.; Garcia-Moreno, I.; López, J. C. 4,4′-Dicyano- versus 4,4′-Difluoro-BODIPYs in Chemoselective Postfunctionalization Reactions: Synthetic Advantages and Applications. *Org. Lett.* **2023**, *25*, 2588−2593.

[2] Kang, J.; Huo, F.; Yue, Y.; Wen, Y.; Chao, J.; Zhang, Y.; Yin, C. A solvent depend on ratiometric fluorescent probe for hypochlorous acid and its application in living cells. *Dyes Pigm.* **2017**, *136*, 852–858.

[3] Hoogendoorn, S.; Blom, A. E. M.; Willems, L. I.; Van der Marel, G. A.; Overkleeft, H. S. Synthesis of pH-Activatable Red Fluorescent BODIPY Dyes with Distinct Functionalities. *Org. Lett.* **2011**, *13*, 5656–5659.

[4] Uriel, C.; Gómez, A. M.; García Martínez de la Hidalga, E.; Bañuelos, J.; Garcia-Moreno, I.; López, J. C. Access to 2,6-Dipropargylated BODIPYs as "Clickable" Congeners of Pyrromethene-567 Dye: Photostability and Synthetic Versatility. *Org. Lett.* **2021**, *23*, 6801−6806.

configuration, instead of the right-angle configuration for the diluted solutions, to reduce the effects of the reabsorption and reemission phenomena.

Fluorescence quantum yields (ϕ) were determined from corrected spectra (detector sensitivity to the wavelength) of diluted solutions (ca. 10^{-6} M) by the optically dilute relative method, using as reference PM567 (ϕ = 0.84 in ethanol). The radiative decay curves were registered with the time correlated single-photon counting technique, as implemented in the aforementioned spectrofluorometer. Fluorescence emission was monitored at the maximum emission wavelength, by means of a microchannel plate detector (Hamamatsu C4878) of picosecond time-resolution (20 ps), after excitation with a Fianium pulsed laser (time resolution of around 150 picoseconds). The fluorescence lifetimes (τ) were obtained after the deconvolution of the instrumental response signal from the recorded decay curves by means of an iterative method. The goodness of the exponential fit was controlled by statistical parameters (chi-square) and the analysis of the residuals.

Laser spectroscopy

Liquid solutions of dyes were contained in 1 cm optical-path rectangular quartz cells, carefully sealed to avoid solvent evaporation during experiments. The liquid solutions were transversely pumped at 532 nm and 355 nm, with 5 mJ, 20 ns FWHM pulses from the second and third harmonic of the *Q*-switched Nd:YAG laser (Lotis TII SI-2132), at a repetition rate of up to 10 Hz. Perpendicularly arranged cylindrical quartz lenses focused the exciting pulses into a narrow horizontal line onto the cell. The oscillation cavity (2 cm length) consisted of a 90% reflectivity aluminum mirror, with the lateral face of the cell as output coupler. The emission from the edge of the sample was monitored perpendicular to the exciting beam, collected by an optical fiber, and imaged onto a spectrograph/monochromator (SpectraPro 300i Acton Research) equipped with a thermoelectrically cooled CCD detector (SpectruMM:GS 128B). The emission was recorded by feeding the signal to the boxcar (Stanford Research, model 250) to be integrated before being digitized and processed by a computer. Pump and output energies were measured with a calibrated Laser Energy Meter (QE 12LP-S-MB-DO, Gentec).

Computational chemistry

The atomic structures of the dye molecules were optimized using Density Functional Theory (DFT) with the Gaussian software package,[5] employing the B3LYP exchange–correlation functional[6] and the 6-311G* basis set. The optimized structures were then packed into a 3 × 3 × 3 nm^3 simulation box using Packmol,[7] arranging four dye molecules at random positions. The simulation box was subsequently saturated with ethanol molecules. Molecular Dynamics (MD) simulations were performed to equilibrate the models using LAMMPS[8] (version June 23, 2022) in combination with the ReaxFF reactive force field[9] An initial energy minimization was followed by equilibration in the isobaric–isothermal (NPT) ensemble at 1 atm and 300 K. The Verlet integration method[10] was used with a time step of 1 fs, while temperature and pressure were controlled with a Nosé-Hoover thermostat and barostat, applying coupling constants of 0.1 ps and 1 ps, respectively. Subsequently, trajectories were recorded in the canonical (NVT) ensemble at 300 K for 5 ns. All DFT and MD simulations were carried out at the DIPC Supercomputing Center.

The resulting trajectories were analyzed to evaluate the stability of intermolecular interactions using the TRAVIS software.[11] In particular, the dimer existence autocorrelation function (DACF) was employed to quantify these interactions. The DACF assesses the probability that a specific criterion (in this case, the existence of a dimer) is continuously fulfilled at a given time (τ), assuming it holds at time 0. This function is defined as:

$$ACF(\tau) = N \langle \sum_{t=0}^{T-\tau} \beta_{ij}(t+\tau) \cdot \beta_{ij}(t) \rangle_{i,j}$$

where β_{ij} is a binary function defined for a given pair of particles *i,j*, which takes the value 1 as long as the criterion (dimer existence) is fulfilled, and switches to 0 as soon as the criterion fails for the first time (when the dimer dissociates). Consequently, the stability of a given dimer is inversely related to the speed at which the DACF decays to zero: less stable dimers dissociate more rapidly, leading to a faster decay of the autocorrelation function.

---

[5] Frisch, M. J.; Trucks, G. W.; Schlegel, H. B.; Scuseria, G. E.; Robb, M. A.; Cheeseman, J. R.; Scalmani, G.; Barone, V.; Petersson, G. A.; Nakatsuji, H. Gaussian 16. Gaussian, Inc. Wallingford, CT 2016.

[6] Kim, K.; Jordan, K. D. Comparison of Density-Functional and Mp2 Calculations on the Water Monomer and Dimer. *J. Phys. Chem.* **1994**, *98* (40), 10089–10094.

[7] Martínez, L.; Andrade, R.; Birgin, E. G.; Martínez, J. M. *PACKMOL: A Package for Building Initial Configurations for Molecular Dynamics Simulations*; Wiley Subscription Services, Inc., A Wiley Company, 2009; Vol. 30, pp 2157–2164.

[8] Thompson, A. P.; Aktulga, H. M.; Berger, R.; Bolintineanu, D. S.; Brown, W. M.; Crozier, P. S.; in't Veld, P. J.; Kohlmeyer, A.; Moore, S. G.; Nguyen, T. D. LAMMPS-a Flexible Simulation Tool for Particle-Based Materials Modeling at the Atomic, Meso, and Continuum Scales. *Comput. Phys. Commun.* **2022**, *271*, 108171.

[9] Liu, Y.; Sun, Q.; Yu, P.; Wu, Y.; Xu, L.; Yang, H.; Xie, M.; Cheng, T.; Goddard III, W. A. Effects of High and Low Salt Concentrations in Electrolytes at Lithium–Metal Anode Surfaces Using DFT-ReaxFF Hybrid Molecular Dynamics Method. *J. Phys. Chem. Lett.* **2021**, *12*, 2922–2929.

[10] Grubmüller, H.; Heller, H.; Windemuth, A.; Schulten, K. Generalized Verlet Algorithm for Efficient Molecular Dynamics Simulations with Long-Range Interactions. *Mol. Simul.* **1991**, *6* (1–3), 121–142.

[11] Brehm, M.; Thomas, M.; Gehrke, S.; Kirchner, B. TRAVIS—A Free Analyzer for Trajectories from Molecular Simulation. *J. Chem. Phys.* **2020**, *152*, 164105.

X-ray diffraction

Single crystals of compound **7a**, exhibiting a ribbon-like habit, were obtained from a methanol solution at 277 K after three months. Diffraction data were collected at 100 K using synchrotron radiation on the BL13-XALOC beamline at ALBA. Data were processed automatically with the DIALS package[12] (Diffraction Integration for Advanced Light Sources) implemented at BL13-XALOC. The structure was solved by the dual-space method using SHELXT, and refined by full-matrix least-squares on F^2 with SHELXL[13] as implemented in Olex2-1.5.[14]

X-ray diffraction analysis showed that this compound crystallizes in the monoclinic space group $P2_1/c$, with one independent molecule in the asymmetric unit. One of the benzyl groups exhibits disorder, which was modelled over two orientations.

CCDC 2479181 contains the supplementary crystallographic data for compound **7a**. These data can be obtained free of charge from The Cambridge Crystallographic Data Centre via www.ccdc.cam.ac.uk/structures

2. General procedures

General Procedure A. Acid Catalyzed Dehydrative reaction of 2,6-di-(hydroxymethyl)-BODIPY

A solution of 2,6-di(hydroxymethyl)-BODIPY **2**, and the corresponding nucleophile (1.5 – 2.0 equivalents per hydroxyl group) in anhydrous CH_2Cl_2 was cooled to –78 °C under an inert atmosphere. $BF_3{\cdot}OEt_2$ (0.5 equiv per hydroxyl group) was added dropwise, and the reaction mixture was stirred at the specified temperature for 0.5–3 h. Upon completion, the reaction mixture was diluted with CH_2Cl_2 and washed sequentially with 1 M NaOH, water, and brine. The organic phase was dried over anhydrous $MgSO_4$, filtered, and concentrated under reduced pressure. The crude product was purified by flash column chromatography on silica gel to afford the corresponding BODIPY derivative.

General procedure B. Copper(I)-catalyzed azide-alkyne cycloaddition reaction (CuAAC)

A mixture of the corresponding azide (0.9–1.5 equiv) and the appropriate alkyne (1.0 equiv) in CH_2Cl_2 was added to a solution of sodium ascorbate (3.0 equiv) and $CuSO_4{\cdot}5H_2O$ (1.5 equiv) in H_2O (CH_2Cl_2/H_2O, 3:1 v/v). The biphasic reaction mixture was transferred to a sealed glass tube and stirred at 65 °C until complete consumption of the starting materials, as monitored by TLC. Upon completion, the mixture was diluted with CH_2Cl_2 and washed with brine. The organic phase was dried over anhydrous $MgSO_4$, filtered, and concentrated under reduced pressure. The crude product was purified by flash column chromatography on silica gel to afford the desired triazole derivative.

[12] Winter, G. DIALS. *Acta Crystallogr. D Biol. Crystallogr.* **2018**, *74*, 85–97.

[13] Sheldrick, G. M. *Acta Crystallogr. C Struct. Chem.* **2015**, *71*, 3–8.

[14] Dolomanov, O. V.; Bourhis, L. J.; Gildea, R. J.; Howard, J. A. K.; Puschmann, H., OLEX2: A complete structure solution, refinement and analysis program **2009**

3. Synthetic route to diol 2

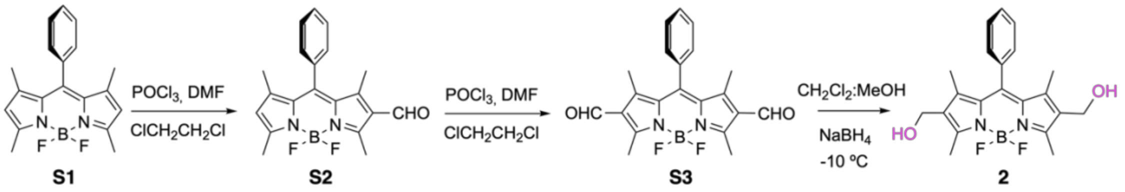

Scheme S1. Detailed synthetic route for 2,6-di-(hydroxymethyl)-1,3,5,7-tetramethyl-8-phenyl BODIPY **2**

4. Synthetic procedures and compound characterization

4.1. Synthesis of 2,6-di-(hydroxymethyl)-1,3,5,7-tetramethyl-8-phenyl BODIPY 2.

To a stirred suspension of $NaBH_4$ (120 mg, 1.05 mmol) in CH_2Cl_2: MeOH (2:1, 30 mL/mmol) under an inert atmosphere and at −10 °C, a solution of dialdehyde **S3** (300 mg, 0.78 mmol) in CH_2Cl_2 (3 mL) was added. The reaction mixture was stirred at 0 °C and monitored by thin-layer chromatography (TLC). After 2 h, Et_3N was added, the solvent was evaporated and the residue purified by flash chromatography (hexane:ethyl acetate, 6:4) to afford compound **2** as an amorphous red solid (177 mg, 59%). ^1H NMR (CD_3OD, 400 MHz) δ 7.56-7.54 (m, 3H), 7.31-7.29 (m, 2H), 4.34 (s, 4H), 2.55 (s, 6H), 1.40 (s, 6H). ^{13}C {^1H} NMR (CD_3OD, 125 MHz) δ 156.6, 143.9, 142.5, 136.5, 132.1, 131.4, 130.5, 130.4, 129.3, 54.1, 12.6, 12.1. ^{11}B NMR (CD_3OD, 128 MHz) δ -0.20 (t, J = 32.2 Hz, 1B).^{19}F NMR (CD_3OD, 376 MHz) δ -146.15 (q, J = 32.2 Hz, 2F). HRMS (ESI/Q-TOF) m/z Calc. for $[M+Na]^+$ $C_{21}H_{23}BF_2N_2NaO_2$: 407.1718; Found: 407.1715; Calc. for $[2M+Na]^+$ $C_{42}H_{46}B_2F_4N_4NaO_4$: 791.3539; Found: 791.3545.

Gram-scale preparation of 2 from S3. A stirred suspension of $NaBH_4$ (403 mg, 3.53 mmol) in CH_2Cl_2/MeOH (2:1, 30 mL per mmol) was prepared under an inert atmosphere at −10 °C. To this mixture, a solution of dialdehyde **S3** (1g, 2.62 mmol) in CH_2Cl_2 (10 mL) was added. The reaction was allowed to stir at 0 °C and its progress was monitored by thin-layer chromatography (TLC). After 2 h, Et_3N was added, and the solvent was removed under reduced pressure. The resulting residue was purified by flash chromatography (hexane/ethyl acetate, 6:4) to yield compound **2** as an amorphous red solid (546 mg, 54%).

4.2. Reaction with C-nucleophiles

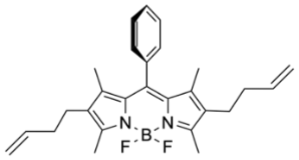

3a

Compound 3a. This compound was prepared according to the general procedure **A** from BODIPY **2** (30 mg, 0.08 mmol), allyltrimethylsilane (37 µL, 0.24 mmol) and $BF_3{\cdot}OEt_2$ (10 µL, 0.08 mmol) in CH_2Cl_2 (4 mL) at –78 °C for 3 h. The reaction crude was diluted with CH_2Cl_2 and washed sequentially with 1 M NaOH, water, and brine. The reaction mixture was dried, filtered, concentrated, and the residue was purified by flash column chromatography on silica gel (hexane:ethyl acetate 95:5) to afford derivative **3a** as an amorphous red solid (28 mg, 81%). ^{1}H NMR ($CDCl_3$, 400 MHz) δ 7.28 – 7.26 (m, 3H), 7.17 – 7.00 (m, 2H), 5.57 (ddt, J = 16.9, 10.1, 6.7 Hz, 2H), 4.92 – 4.65 (m, 4H), 2.32 (s, 6H), 2.22 – 2.07 (m, 4H), 1.99 – 1.72 (m, 4H), 1.07 (s, 6H). ^{13}C {^{1}H} NMR ($CDCl_3$, 125 MHz) δ 154.1, 140.5, 139.1, 138.0, 135.8, 131.0, 130.6, 129.2, 128.9, 128.3, 115.3, 34.4, 23.7, 12.8, 12.03. ^{19}F NMR ($CDCl_3$, 376 MHz) δ -145.70 (q, J = 32.4 Hz, 2F). HRMS (ESI/Q-TOF) m/z Calc. for $[M+H]^+$ $C_{27}H_{32}BF_2N_2$: 433.2626; Found: 433.2642.

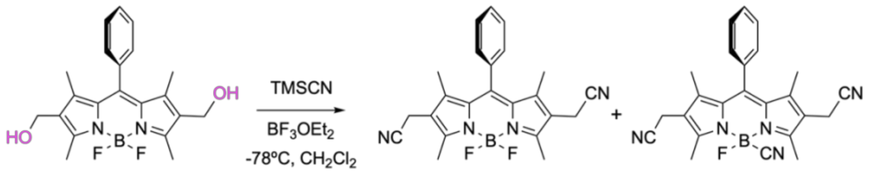

Scheme S2. Reaction of 2,6-di-(hydroxymethyl) BODIPY **2** with TMSCN

Compound 3b. BODIPY **2** (30 mg, 0.08 mmol) was reacted with trimethylsilyl cyanide (62 µL, 0.48 mmol) and $BF_3{\cdot}OEt_2$ (10 µL, 0.08 mmol) in CH_2Cl_2 (4 mL) at –78 °C following general procedure **A**. After 1 h, the reaction mixture was diluted with CH_2Cl_2 and washed sequentially with 1 M NaOH, water, and brine. The solution was dried, filtered, concentrated under reduced pressure, and the crude was purified by flash chromatography on silica gel (hexane:ethyl acetate 6:4) to yield derivative **3b** (10 mg, 32%) followed by **3b-CN** (19 mg, 54%) (Scheme S2). For **3b**: ^{1}H NMR ($CDCl_3$, 400 MHz) δ 7.56 – 7.52 (m, 3H), 7.29 – 7.26 (m, 2H), 3.38 (s, 4H), 2.61 (s, 6H), 1.38 (s, 6H). ^{13}C {^{1}H} NMR ($CDCl_3$, 125 MHz) δ 154.1, 143.3, 141.0, 134.3, 131.0, 129.6, 129.5, 127.7, 119.5, 116.6, 12.7, 12.1. ^{11}B NMR ($CDCl_3$, 128 MHz) δ 0.57 (t, J = 32.5 Hz, 1B). ^{19}F NMR ($CDCl_3$, 376 MHz) δ -146.26 (q, J = 32.3 Hz, 2F). HRMS (ESI/Q-TOF) m/z Calc. for $[M-H]^-$ $C_{23}H_{20}BF_2N_4$: 401.1759; Found: 401.1756. **3b-CN**: ^{1}H NMR ($CDCl_3$, 400 MHz) δ 7.58 – 7.55 (m, 3H), 7.31 – 7.26 (m, 2H), 3.41 (s, 1H), 2.69 (d, J = 1.4 Hz, 1H), 1.41 (s, 2H). ^{11}B NMR ($CDCl_3$, 128 MHz) δ -5.42 (d, J = 48.0 Hz, 1B). ^{19}F NMR ($CDCl_3$, 376 MHz) δ -160.44 (q, J = 47.6 Hz, 1F). HRMS (ESI/Q-TOF) m/z Calc. for $[M-H]^-$ $C_{24}H_{20}BFN_5$: 408.1806; Found: 408.1805. HRMS (ESI/Q-TOF) m/z Calc. for $[M-Na]^+$ $C_{24}H_{21}BFN_5Na$: 432.1771; Found: 432.1771**.**

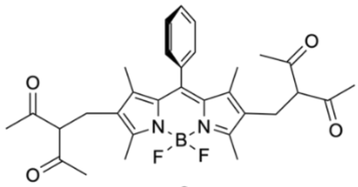

3c

Compound 3c. Following general procedure **A**, BODIPY **2** (30 mg, 0.08 mmol) in CH_2Cl_2 (4 mL) at –78 °C was reacted with pentane-2,4-dione (34 µL, 0.32 mmol) and $BF_3{\cdot}OEt_2$ (10 µL, 0.08 mmol). After 1 h, the reaction mixture was diluted with CH_2Cl_2 and washed sequentially with 1 M NaOH, water, and brine. The organic layer was dried, filtered, concentrated under reduced pressure, and the crude product was purified by flash chromatography on silica gel (hexane:ethyl acetate 7:3) to yield derivative **3c** as an amorphous red solid (30 mg, 71%). ^{1}H NMR ($CDCl_3$, 400 MHz) δ 7.52 – 7.45 (m, 3H), 7.25 – 7.14 (m, 2H), 3.74 (t, *J* = 7.3 Hz, 2H), 2.82 (d, *J* = 7.3 Hz, 4H), 2.53 (s, 6H), 2.11 (s, 12H), 1.27 (s, 6H). $^{13}C\{^{1}H\}$ NMR ($CDCl_3$, 125 MHz) δ 203.3, 154.4, 141.6, 140.2, 135.2, 131.2, 129.5, 129.4, 129.3, 128.1, 128.0, 127.2, 67.8, 30.1, 22.8, 12.9, 12.2. ^{11}B NMR ($CDCl_3$, 128 MHz) δ 0.62 (t, *J* = 33.0 Hz, 1B). ^{19}F NMR ($CDCl_3$, 376 MHz) δ -146.76 (q, *J* = 32.4 Hz, 2F). HRMS (ESI/Q-TOF) m/z Calc. for $[M+Na]^+$ $C_{31}H_{35}BF_2N_2NaO_4$: 571.2556; Found: 571.2548.

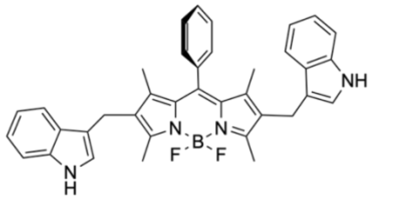

Compound 3d. According to general procedure **A**, a solution of BODIPY **2** (30 mg, 0.08 mmol), in CH_2Cl_2 (4 mL) was cooled to –50 °C and then treated with indole (27 mg, 0.24 mmol) and $BF_3{\cdot}OEt_2$ (10 µL, 0.08 mmol). The reaction mixture was stirred for 30 min, diluted with CH_2Cl_2 and washed sequentially with 1 M NaOH, water, and brine. The organic layer was dried, filtered, and then concentrated. The crude material was purified by flash column chromatography on silica gel (hexane:ethyl acetate 7:3), to afford derivative **3d** as an amorphous red solid (33 mg, 73%). ^{1}H NMR ($CDCl_3$, 400 MHz) δ 7.91 (s, 2H), 7.60 (d, *J* = 7.8 Hz, 2H), 7.50 – 7.41 (m, 3H), 7.35 – 7.30 (m, 4H), 7.25 – 7.15 (m, 2H), 7.15 – 7.08 (m, 2H), 6.63 (s, 2H), 3.77 (s, 4H), 2.54 (s, 6H), 1.32 (s, 6H). $^{13}C\{^{1}H\}$ NMR ($CDCl_3$, 125 MHz) δ 154.8, 140.8, 139.8, 136.6, 135.7, 131.1, 129.4, 129.2, 129.0, 128.4, 127.2, 122.2, 122.0, 119.5, 118.8, 114.8, 111.3, 19.98, 12.9, 12.2. ^{11}B NMR ($CDCl_3$, 128 MHz) δ 0.88 (t, *J* = 33.9 Hz, 1B). ^{19}F NMR ($CDCl_3$, 376 MHz) δ -146.33 (q, *J* = 30.7 Hz, 2F). HRMS (ESI/Q-TOF) m/z Calc. for $[M+Na]^+$ $C_{37}H_{33}BF_2N_4Na$: 605.2665; Found: 605.2662.

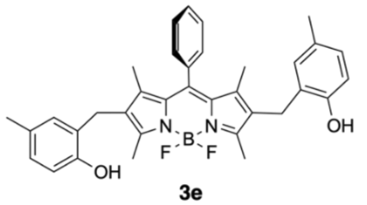

3e

***Compound 3e*.** According to general procedure **A**, a solution of BODIPY **2** (30 mg, 0.08 mmol), *p*-cresol (34 mg, 0.32 mmol) and $BF_3 \cdot OEt_2$ (10 μL, 0.08 mmol) in CH_2Cl_2 (4 mL) was stirred at –78 °C for 2 h 30 min. The reaction mixture was then diluted with CH_2Cl_2 and washed sequentially with 1 M NaOH, water, and brine. The organic layer was dried, filtered, concentrated, and the crude material was purified by flash column chromatography on silica gel (hexane:ethyl acetate 7:3), affording derivative **3e** as an amorphous red solid (31 mg, 69%). ^1H NMR ($CDCl_3$, 400 MHz) δ 7.48 – 7.43 (m, 3H), 7.34 – 7.29 (m, 2H), 6.84 – 6.82 (m, 2H), 6.62 – 6.60 (s, 4H), 4.79 (s, 2H), 3.62 (s, 4H), 2.50 (s, 6H), 2.19 (s, 6H), 1.27 (s, 6H).^{13}C {^1H} NMR ($CDCl_3$, 125 MHz) δ 155.2, 151.2, 140.9, 140.4, 135.6, 131.2, 130.1, 129.9, 129.2, 129.0, 128.5, 128.4, 127.7, 125.6, 115.1, 23.9, 20.8, 13.0, 12.2.^{11}B NMR ($CDCl_3$, 128 MHz) δ: 0.84 (t, *J* = 32.9 Hz, 1B).^{19}F NMR ($CDCl_3$, 376 MHz) δ -145.09 (q, *J* = 31.1 Hz, 2F). HRMS (ESI/Q-TOF) m/z Calc. for $[M+Na]^+$ $C_{35}H_{35}BF_2N_2NaO_2$: 587.2658; Found: 587.2654.

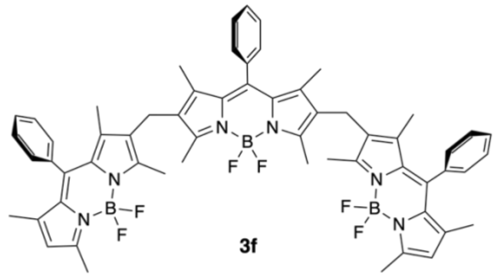

3f

Compound 3f. Compound **3f** was prepared according to the general procedure **A** from BODIPY **2** (50 mg, 0.13 mmol), BODIPY **S1** (168 mg, 0.52 mmol) and $BF_3 \cdot OEt_2$ (8 μL, 0.064 mmol) in CH_2Cl_2 (8 mL) at –78 °C for 30 min. The reaction crude was diluted with CH_2Cl_2 and washed sequentially with 1 M NaOH, water, and brine. The organic layer was dried, filtered, concentrated, and the residue was purified by flash column chromatography on silica gel (hexane:ethyl acetate 95:5), to afford derivative **3f** as an amorphous red solid (102 mg, 75%). ^1H NMR ($CDCl_3$, 400 MHz) δ 7.54 – 7.39 (m, 9H), 7.25 – 7.18 (m, 6H), 6.27 – 5.62 (m, 2H), 3.31 (s, 4H), 2.53 (s, 6H), 2.43 (s, 6H), 2.41 (s, 6H), 1.33 (s, 6H), 1.19 (s, 6H), 1.16 (s, 6H). ^{13}C {^1H} NMR ($CDCl_3$, 125 MHz) δ 155.2, 154.9, 154.4, 143.1, 141.5, 141.0, 139.8, 139.6, 135.6, 135.4, 131.5, 131.11, 131.0, 129.4,129.3, 129.1, 128.4, 128.3, 128.1, 121.3, 19.7, 14.8, 14.6, 13.3, 13.2, 13.1, 12.3. ^{11}B NMR (128 MHz, $CDCl_3$) δ 0.67 (t, *J* = 33.0 Hz, 1B).^{19}F NMR (376 MHz, $CDCl_3$) δ -146.14 (q, *J* = 29.4 Hz, 2F), -146.73 (q, *J* = 31.1 Hz, 4F). HRMS (ESI/Q-TOF) m/z Calc. for $[M+Na]^+$ $C_{59}H_{57}B_3F_6N_6Na$: 1019.4746; Found: 1019.4744.

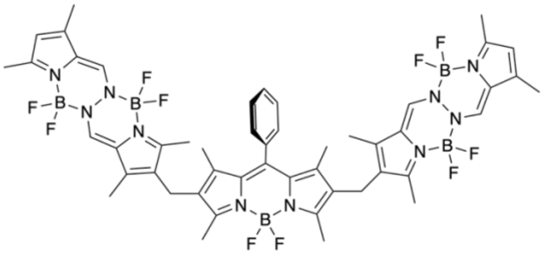

3g

Compound 3g. According to general procedure **A**, a solution of BODIPY **2** (20 mg, 0.05 mmol), 1,3,6,8-tetramethyl BOPHY[15] (70 mg, 0.2 mmol) and $BF_3{\cdot}OEt_2$ (5 μL, 0.04mmol) in CH_2Cl_2 (4 mL) was stirred at –50 °C for 1 h 30 min. The reaction crude was diluted with CH_2Cl_2 and washed sequentially with 1 M NaOH, water, and brine. The organic layer was dried, filtered, concentrated and the crude material was purified by flash column chromatography on silica gel (hexane:ethyl acetate 7:3), affording derivative **3g** as an amorphous red solid (43 mg, 85%). ^{1}H NMR ($CDCl_3$, 400 MHz) δ 7.91 (s, 2H), 7.86 (s, 2H), 7.49– 7.45 (m, 3H), 7.26 – 7.25 (m, 2H), 6.17 (s, 2H), 3.47 (s, 4H), 2.48 (s, 6H), 2.47 (s, 6H), 2.34 (s, 6H), 2.32 (s, 6H), 2.11 (s, 6H), 1.21 (s, 6H). ^{13}C {^{1}H} NMR ($CDCl_3$, 125 MHz) δ 154.2, 151.2, 149.7, 141.3, 141.0, 139.6, 137.5, 135.2, 134.4, 133.9, 130.9, 129.3, 129.1, 128.1, 127.6, 126.5, 123.4, 122.6, 118.5, 19.6, 14.1, 12.9, 12.6, 12.2, 11.1, 9.7. ^{11}B NMR (128 MHz, $CDCl_3$) δ 0.65 (*bs*, 5B). ^{19}F NMR (376 MHz, $CDCl_3$) δ -142.70 (m, 4F), -143.29 (m, 4F), -146.25 (q, *J* = 30.3 Hz, 2F). HRMS (ESI/Q-TOF) m/z Calc. for $[M+Na]^+$ $C_{49}H_{51}B_5F_{10}N_{10}Na$: 1046.4530; Found: 1046.4518.

4.3. Reaction with S-nucleophiles

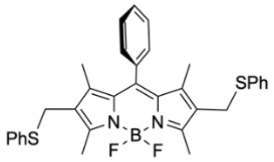

3h

Compound 3h. According to general procedure **A**, a solution of BODIPY **2** (200 mg, 0.52 mmol) and thiophenol (212 μl, 2.05 mmol) in CH_2Cl_2 (10 mL) was stirred at –50 °C; $BF_3{\cdot}OEt_2$ (65 μl, 0.52 mmol) was then added dropwise, and the resulting mixture was stirred for 2h. Then, it was diluted with CH_2Cl_2 and washed sequentially with 1 M NaOH, water, and brine. The reaction mixture was dried, filtered, concentrated and the crude material was purified by flash column chromatography on silica gel (hexane:ethyl acetate 8:2) affording derivative **3h** as an amorphous red solid (279.6 mg, 95%). ^{1}H NMR ($CDCl_3$, 400 MHz) δ 7.49-7.24 (m, 15H), 3.82 (s, 4H), 2.47 (s, 6H) 1.22 (s, 6H). ^{13}C {^{1}H} NMR ($CDCl_3$, 125 MHz) δ 155.1, 141.6, 140.6, 135.8, 135.1, 131.6, 130.9, 129.1, 129.1, 128.9, 128.0, 127.0, 125.9, 29.0, 12.5, 11.8. ^{11}B NMR ($CDCl_3$, 128 MHz) δ: 0.64 (t, *J* = 32.9 Hz, 1B).^{19}F NMR ($CDCl_3$, 376 MHz) δ -146.5 (q, *J* = 32.9 Hz, 2F). HRMS (ESI/Q-TOF) m/z Calc. for $[M+Na]^+$ $C_{33}H_{31}BF_2N_2S_2$: 591.1888; Found: 591.1899.

[15] Tamgho, I.-S.; Hasheminasab, A.; Engle, J. T.; Nemykin, V. N.; Ziegler, C. J. A New Highly Fluorescent and Symmetric Pyrrole–BF_2 Chromophore: BOPHY. *J. Am. Chem. Soc.* **2014**, *136*, 5623–5626.

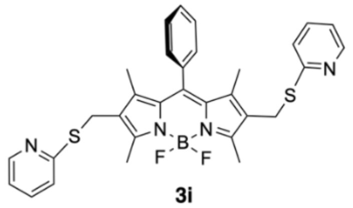

3i

Compound 3i. Compound **3i** was prepared according to the general procedure **A** from BODIPY **2** (30 mg, 0.08 mmol), 2-mercaptopyridine (26 mg, 0.24 mmol) and $BF_3{\cdot}OEt_2$ (10 μL, 0.08 mmol) in CH_2Cl_2 (4 mL) at –78 °C for 2 h. The reaction crude was diluted with CH_2Cl_2 and washed sequentially with 1 M NaOH, water, and brine. The reaction mixture was dried, filtered, concentrated and the residue was purified by flash column chromatography on silica gel (hexane:ethyl acetate 7:3) to afford derivative **3i** as an amorphous red solid (36 mg, 82%).^{1}H NMR ($CDCl_3$, 400 MHz) δ 8.43 (ddd, *J* = 4.9, 1.9, 1.0 Hz, 2H), 7.49 – 7.41 (m, 5H), 7.28 – 7.25 (m, 2H), 7.13 (dt, *J* = 8.1, 1.0 Hz, 2H), 6.98 (ddd, *J* = 7.4, 5.0, 1.1 Hz, 2H), 4.19 (s, 4H), 2.62 (s, 6H), 1.37 (s, 6H). ^{13}C {^{1}H} NMR ($CDCl_3$, 125 MHz) δ 159.1, 155.3, 149.4, 141.6, 141.0, 136.1, 135.3, 131.2, 129.3, 129.2, 128.2, 125.8, 122.4, 119.7, 24.3, 12.9, 12.2. ^{11}B NMR ($CDCl_3$, 128 MHz) δ 0.74 (t, *J* = 33.0 Hz, 1B).^{19}F NMR ($CDCl_3$, 376 MHz) δ -146.45 (q, *J* = 32.3 Hz, 2F). HRMS (ESI/Q-TOF) m/z Calc. for $[M+Na]^+$ $C_{31}H_{29}B\,F_2N_4NaS_2$: 593.1793; Found: 593.1793.

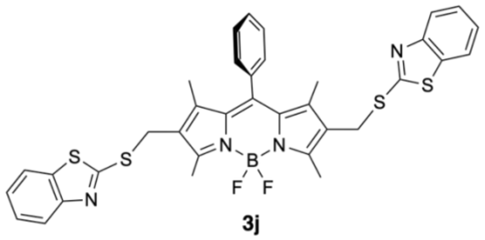

3j

Compound 3j. BODIPY **2** (38 mg, 0.1 mmol) was reacted with 2-mercaptobenzothiazole (50 mg, 0.3 mmol) and $BF_3{\cdot}OEt_2$ (10 μL, 0.08 mmol) in CH_2Cl_2 (4 mL) at –78 °C for 1 h, following general procedure **A**. After completion, the reaction crude was diluted with CH_2Cl_2 and washed sequentially with 1 M NaOH, water, and brine. The organic layer was dried, filtered, concentrated under reduced pressure, and the crude product was purified by flash chromatography on silica gel (hexane:ethyl acetate 9:1) to yield derivative **3j** as an amorphous red solid (61 mg, 92%).^{1}H NMR ($CDCl_3$, 400 MHz) δ 7.87 (d, *J* = 8.0 Hz, 2H), 7.75 (d, *J* = 8.1 Hz, 2H), 7.52 – 7.46 (m, 3H), 7.42 (ddd, *J* = 8.3, 7.2, 1.2 Hz, 2H), 7.30 (ddd, *J* = 8.3, 7.3, 1.2 Hz, 2H), 7.28 – 7.23 (m, 2H), 4.41 (s, 4H), 2.67 (s, 6H), 1.40 (s, 6H). ^{13}C {^{1}H} NMR ($CDCl_3$, 125 MHz) δ 166.1, 155.5, 153.2, 142.3, 141.6, 135.4, 134.9, 131.3, 129.4, 129.4, 128.0, 126.2, 124.5, 121.6, 121.2, 27.6, 12.9, 12.2. ^{11}B NMR ($CDCl_3$, 128 MHz) δ -4.09 (t, *J* = 32.7 Hz, 1B).^{19}F NMR ($CDCl_3$, 376 MHz) δ -151.02 (q, *J* = 31.9 Hz, 2F). HRMS (ESI/Q-TOF) m/z Calc. for $[M+Na]^+$ $C_{35}\,H_{29}\,B\,F_2\,N_4\,NaS_4$: 705.1235; Found: 705.1225.

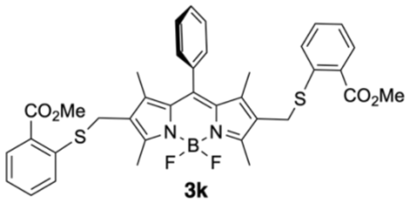

3k

Compound 3k. BODIPY **2** (30 mg, 0.08 mmol) was reacted with methyl 2-mercaptobenzoate[16] (52.5 mg, 0.32 mmol) and $BF_3·OEt_2$ (10 µL, 0.08 mmol) in CH_2Cl_2 (4 mL) at –78 °C for 2 h, following general procedure **A**. After completion, it was diluted with CH_2Cl_2 and washed sequentially with 1 M NaOH, water, and brine. The reaction mixture was dried, filtered, and concentrated under reduced pressure, and the crude product was purified by flash chromatography on silica gel (hexane:ethyl acetate 7:3 to 1:1) to yield derivative **3c** as an amorphous red solid (26.2 mg, 49%).^{1}H NMR ($CDCl_3$, 400 MHz) δ 7.96-7.94 (m, 2H), 7.48-7.45 (m, 5H), 7.38-7.36 (m, 2H), 7.29-7.27 (m, 2H), 7.22-7.18 (m, 2H), 3.89 (s, 6H), 3.86 (s, 4H), 2.58 (s, 6H), 1.33 (s, 6H).^{13}C {^{1}H} NMR ($CDCl_3$, 125 MHz) δ 167.1, 155.4, 142.0, 141.9, 141.4, 135.2, 132.5, 131.3, 131.2, 129.3, 129.2, 128.3, 128.2, 126.8, 124.5, 124.3, 52.3, 27.0, 12.8, 12.7, 12.1.^{11}B NMR ($CDCl_3$, 128 MHz) δ 0.65 (t, *J* = 32.8 Hz, 1B).^{19}F NMR ($CDCl_3$, 376 MHz) δ -146.38 (q, *J* = 31.9 Hz). HRMS (ESI/Q-TOF) m/z Calc. for $[M+Na]^+$ $C_{37}H_{35}BF_2N_2NaO_4S_2$: 707.1997; Found: 707.1988.

4.4. Reaction with a hydride nucleophile

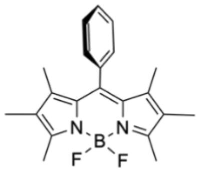

3l

Compound 3l. BODIPY **2** (40 mg, 0.1 mmol) was reacted with triethylsilane (63 µL, 0.4 mmol) and $BF_3·OEt_2$ (12.5 µL, 0.1 mmol) in CH_2Cl_2 (5 mL) at –78 °C for 1 h, following general procedure **A**. After completion, it was diluted with CH_2Cl_2 and washed sequentially with 1 M NaOH, water, and brine. The reaction mixture was dried, filtered, concentrated under reduced pressure, and the crude product was purified by flash chromatography on silica gel (hexane:ethyl acetate 95:5) to yield derivative **3l** as an amorphous red solid (40 mg, 88%).^{1}H NMR ($CDCl_3$, 400 MHz) δ 7.50 – 7.32 (m, 3H), 7.27 – 7.06 (m, 2H), 2.44 (s, 6H), 1.77 (s, 6H), 1.19 (s, 6H). ^{13}C {^{1}H} NMR ($CDCl_3$, 125 MHz) δ 154.1, 140.2, 139.0, 135.9, 130.8, 129.2, 129.1, 128.9, 128.9, 128.4, 128.37, 126.5, 12.8, 12.0, 9.1.^{11}B NMR ($CDCl_3$, 128 MHz) δ: 0.75 (t, *J* = 33.6 Hz, 1B). ^{19}F NMR ($CDCl_3$, 376 MHz) δ: -146.82 (q, *J* = 33.3 Hz, 2F). HRMS (ESI/Q-TOF) m/z Calc. for $[M+H]^+$ $C_{21}H_{24}BF_2N_2$: 353.1999; Found: 353.2014. Calc. for $[M+Na]^+$ $C_{21}H_{23}BF_2N_2Na$: 375.1818; Found: 375.1830.

---

[16] Viani, F.; Rossi, B.; Panzeri, W.; Merlini, L.; Martorana, A.M.; Polissi, A.; Galante, Y. M. Synthesis and anti-bacterial activity of a library of 1,2-benzisothiazol-3(2H)-one (BIT) derivatives amenable of crosslinking to polysaccharides. *Tetrahedron* **2017**, *73*, 1745–1761.

4.5. Reaction with an N-*Nucleophile*

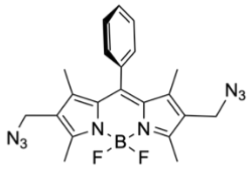

3m

***Compound 3m*.** According to general procedure **A**, a solution of BODIPY **2** (223 mg, 0.58 mmol) trimethylsilyl azide (460 μL, 3.48 mmol) and $BF_3 \cdot OEt_2$ (70μL, 0.58 mmol) in CH_2Cl_2 (20 mL) was stirred at 0 °C for 2 h. The reaction crude was diluted with CH_2Cl_2 and washed sequentially with 1 M NaOH, water, and brine. The reaction mixture was dried, filtered, concentrated and the crude material was purified by flash column chromatography on silica gel (hexane: ethyl acetate 95:5) affording derivative **3m** as an amorphous red solid (240 mg, 94%). ^1H NMR ($CDCl_3$, 400 MHz) δ 7.53 – 7.52 (m, 3H), 7.31 – 7.29 (m, 2H), 4.10 (s, 4H), 2.62 (s, 6H), 1.39 (s, 6H). ^{13}C {^1H} NMR ($CDCl_3$, 125 MHz) δ 155.5, 143.1, 142.1, 134.8, 131.3, 129.5, 129.5, 127.9, 124.9, 44.0, 12.7, 12.1. ^{11}B NMR ($CDCl_3$, 128 MHz) δ: -0.22 (t, *J* = 32.7 Hz, 1B). ^{19}F NMR ($CDCl_3$, 376 MHz) δ -145.25 (q, *J* = 33.6 Hz, 2F). HRMS (ESI/Q-TOF) m/z Calc. for $[M-H]^-$ $C_{21}H_{21}BF_2N_8$ 433.1881; Found: 433.1876.

Gram-scale preparation of 3m from BODIPY 2. According to general procedure **A**, a solution of 2,6-di(hydroxymethyl)-BODIPY **2** (1g, 2.6 mmol) and trimethylsilyl azide (2.06 ml, 15.6 mmol) in CH_2Cl_2 (60 ml) was cooled to 0 °C under an inert atmosphere and treated with $BF_3 \cdot OEt_2$ (314 μL, 2.6 mmol). The reaction mixture was stirred at 0 °C for 2 h 30 min. After work-up the crude product was purified by flash column chromatography on silica gel (hexane: ethyl acetate 95:5) to afford derivative **3m** as an amorphous red solid (1.02 g, 90%).

4.6. Reaction with an O- Nucleophile

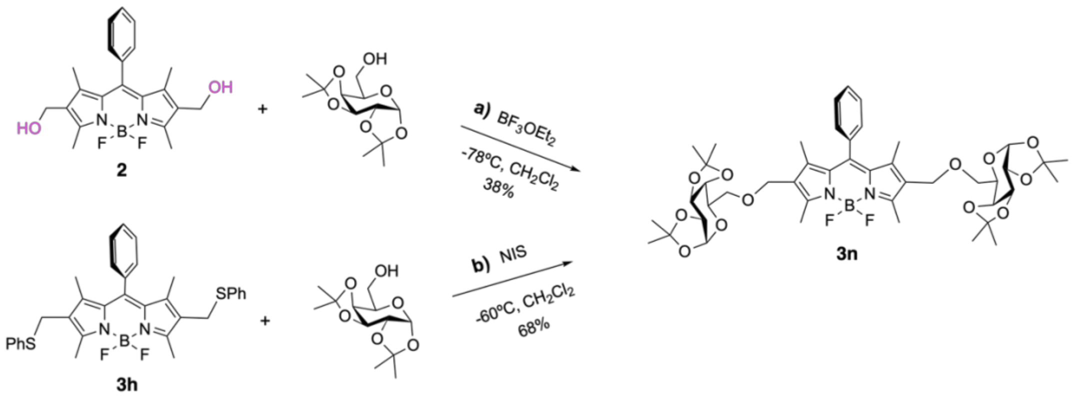

Scheme S3. Synthesis of BODIPY **3n** from diol **2,** and from dithioether **3h.**

Compound 3n from diol 2. This compound was prepared according to general procedure **A** (Scheme **S3a**) from BODIPY **2** (25 mg, 0.065 mmol), 1,2:3,4-di-*O*-isopropylidene-α-D-galactopyranose (51 mg, 0.195 mmol) and $BF_3{\cdot}OEt_2$ (8 μL, 0.064 mmol) in CH_2Cl_2 (8 mL) was stirred at -78 °C for 30 min and then at -50 °C for 4 h. Then, the reaction mixture was diluted with CH_2Cl_2 and washed sequentially with 1 M NaOH, water, and brine. The reaction mixture was dried, filtered, concentrated and the crude material was purified by flash column chromatography The residue was purified by flash chromatography (ethyl acetate: methanol 95:5) to give **3n** as an amorphous red solid (15 mg, 38%). ^1H NMR ($CDCl_3$, 400 MHz) δ 7.48-7.46 (m, 3H), 7.27-7.24 (m, 2H), 5.51 (d, *J* = 5.0 Hz, 2H), 4.56 (dd, *J* = 8.0, 2.4 Hz, 2H), 4.37 (d, *J* = 11.6 Hz, 2H), 4.28 (dd, *J* = 5.1, 2.4 Hz, 2H), 4.26-4.20 (m, 4H), 3.94 (td, *J* = 6.4, 1.9 Hz, 2H), 3.60 (dd, *J* = 10.0, 6.4 Hz, 2H), 3.55 (dd, *J* = 10.0, 6.6 Hz, 2H), 2.60 (s, 6H), 1.49 (s, 6H), 1.42 (s, 6H), 1.36 (s, 6H), 1.31 (s, 12H).^{13}C {^1H} NMR ($CDCl_3$, 125 MHz) δ 156.1, 142.2, 141.8, 135.4, 131.1, 129.3, 129.1, 128.2, 127.3, 109.2, 108.2, 96.4, 71.1, 70.7, 68.4, 67.0, 62.9, 26.2, 26.1, 25.0, 24.4, 12.7, 11.9.^{11}B NMR ($CDCl_3$, 128 MHz) δ 0.71 (t, *J* = 32.8 Hz, 1B).^{19}F NMR ($CDCl_3$, 376 MHz) δ -146.38 (q, *J* = 30.7 Hz, 2F). HRMS (ESI/Q-TOF) m/z Calc. for $[M+Na]^+$ $C_{45}H_{59}BF_2N_2NaO_{12}$: 891.4027; Found: 891.4034. $[\alpha]_D^{20}$ -865.554 (*c* 0.54, $CHCl_3$).

Compound 3n from dithioether 3h. This compound was also prepared, in a preferred manner (Scheme **S3b**) from BODIPY **3h** (30 mg, 0.053 mmol), 1,2:3,4-di-*O*-isopropylidene-α-D-galactopyranose (41 mg, 0.158 mmol) and *N*-iodosuccinimide (35 mg, 0.158 mmol) at -60 °C. Upon completion (1 h), the reaction mixture was diluted with CH_2Cl_2 and washed with a 10% $Na_2S_2O_3$ aqueous solution. The organic phase was dried over anhydrous $MgSO_4$, filtered, and concentrated under reduced pressure The residue was purified by flash chromatography (toluene: ethyl acetate 8:2) to give **3n** as an amorphous red solid (31.3 mg, 68%).

5. *Click CuAAC reactions of bis-azidomethyl BODIPY 3l.*

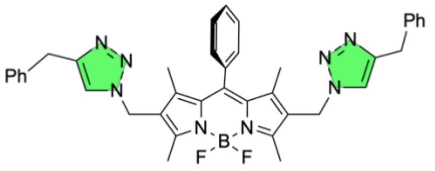

7a

Compound 7a. Following the general procedure **B**, bis-azidomethyl BODIPY **3l** (43.4 mg, 0.1 mmol) and 3-phenyl-1-propine (37 μl, 0.3 mmol) dissolved in CH_2Cl_2 (4 mL) were added to a solution of sodium ascorbate (60 mg, 0.3 mmol) and $CuSO_4$ (37mg, 0.15 mmol) in H_2O (3 mL). The solution was placed in a glass seal tube and heated (65 °C) for 24h. After cooling and work up , the residue was purified by silica column chromatography (hexane: ethyl acetate 1:1 to ethyl acetate) to afford BODIPY **7a** (60.1 mg, 90%).^{1}H NMR ($CDCl_3$, 400 MHz) δ 7.64 – 7.46 (m, 3H), 7.39 – 7.18 (m, 12H), 7.02 (s, 2H), 5.24 (s, 4H), 4.06 (s, 4H), 2.60 (s, 6H), 1.39 (s, 6H). ^{13}C {^{1}H} NMR ($CDCl_3$, 125 MHz) δ 155.4, 147.9, 143.7, 142.5, 139.1, 134.3, 131.3, 129.7, 129.6, 128.7, 128.7, 127.7, 126.6, 124.1, 120.6, 43.5, 32.3, 12.8, 12.2. ^{11}B NMR ($CDCl_3$, 128 MHz) δ -0.30 (t, *J* = 33.0 Hz, 1B).^{19}F NMR ($CDCl_3$, 376 MHz) δ -144.99 (q, *J* = 31.7 Hz, 2F). HRMS (ESI/Q-TOF) m/z Calc. for $[M+Na]^+$ $C_{39}H_{37}BF_2N_8Na$: 689.3101; Found: 689.3100.

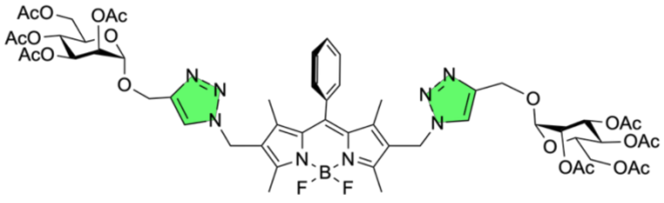

7b

Compound 7b. Following the general procedure **B**, a solution of bis-azidomethyl BODIPY **3l** (43.4 mg, 0.1 mmol) and tetra-*O*-acetyl propargyl-α-D-mannopyranoside (116 mg, 0.3 mmol) in CH_2Cl_2 (4 mL) was added to a solution of sodium ascorbate (60 mg, 0.3 mmol) and $CuSO_4$ (37mg, 0.15 mmol) in H_2O (3 mL). The biphasic mixture was placed in a glass seal tube and heated (65 °C) for 24h. The residue was purified by silica column chromatography (hexane: ethyl acetate 3:7 to ethyl acetate) to afford BODIPY **7b** (100.1 mg, 83%).^{1}H NMR ($CDCl_3$, 400 MHz) δ 7.52-7.50 (m, 3H), 7.38 (s, 2H), 7.28-7.26 (m, 2H), 5.27 (m, 8H), 5.19 (s, 2H), 4.90 (d, *J* = 1.8 Hz, 2H), 4.77 (d, *J* = 12.3 Hz, 2H), 4.60 (d, *J* = 12.3 Hz, 2H), 4.26 (dd, *J* = 12.1, 5.0 Hz, 2H), 4.14 – 3.93 (m, 4H), 2.59 (s, 6H), 2.11 (s, 6H), 2.08 (s, 6H), 2.00 (s, 6H), 1.95 (s, 6H), 1.41 (s, 6H). ^{13}C {^{1}H} NMR ($CDCl_3$, 125 MHz) δ 170.8, 170.1, 170.0, 169.8, 155.5, 144.0, 143.8, 142.6, 134.3, 131.4, 129.7, 127.7, 123.8, 122.2, 96.9, 69.5, 69.0, 68.7, 66.1, 62.4, 61.0, 43.6, 20.9, 20.8, 20.7, 12.8, 12.2. ^{11}B NMR ($CDCl_3$, 128 MHz) δ -0.29 (t, *J* = 32.1 Hz, 1B). ^{19}F NMR ($CDCl_3$, 376 MHz) δ -144.93 (q, *J* = 30.5 Hz, 2F). HRMS (ESI/Q-TOF) m/z: Calc. for $[M+Na]^+$ $C_{55}H_{67}BF_2N_8NaO_{20}$: 1229.4278 Found: 1229.4276. $[\alpha]_D^{20}$ + 25.1 (*c* 0.56, $CHCl_3$).

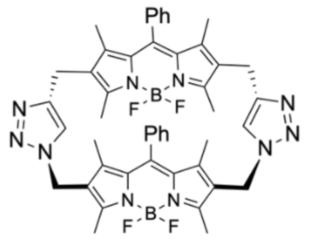

10

Compound 10. To a stirred solution of 2,6-bis-propargyl BODIPY **8** 25 mg (0.06 mmol) and 2,6-bis-azido derivative **3m**, 27 mg (0.06 mmol) in THF: H_2O/ 3:1 (25 mL), $CuSO_4{\cdot}5H_2O$ (23.2 mg, 0.09 mmol) and sodium ascorbate (36.8 mg, 0.186 mmol) were added and the mixture stirred at 80 °C in a sealed tube. After 1 h, TLC indicated complete consumption of the starting material. The reaction was diluted with EtOAc (25 mL) and washed with brine (10 mL). The organic layer was dried ($MgSO_4$), filtered and concentrated. The crude residue was purified by silica gel chromatography (hexane: ethyl acetate 3:7 to ethyl acetate) to afford the desired 1,4-disubstituted 1,2,3-triazole **10** as an amorphous red solid (9 mg, 18%). ^{1}H NMR (DMSO-d6, 400 MHz) δ (selected signals): 7.52-7.50 (m, 10H), 6.10 (m, 2H), 5.24-5.21 (m, 4H), 3.73 (m, 4H), 2.44 (s, 6H), 2.37 (s, 6H), 1.25 (s, 6H), 1.21 (s, 6H). HRMS (ESI/Q-TOF) m/z: Calc. for $[M+Na]^+$ $C_{46}H_{44}B_2F_4N_{10}Na$: 857.3770 Found: 857.3761; Calc. for $[M+H]^+$ $C_{46}H_{45}B_2F_4N_{10}$: 835.3951; Found: 835.3943.

6. *Plausible mechanism for the formation of compounds 3a and 3l*

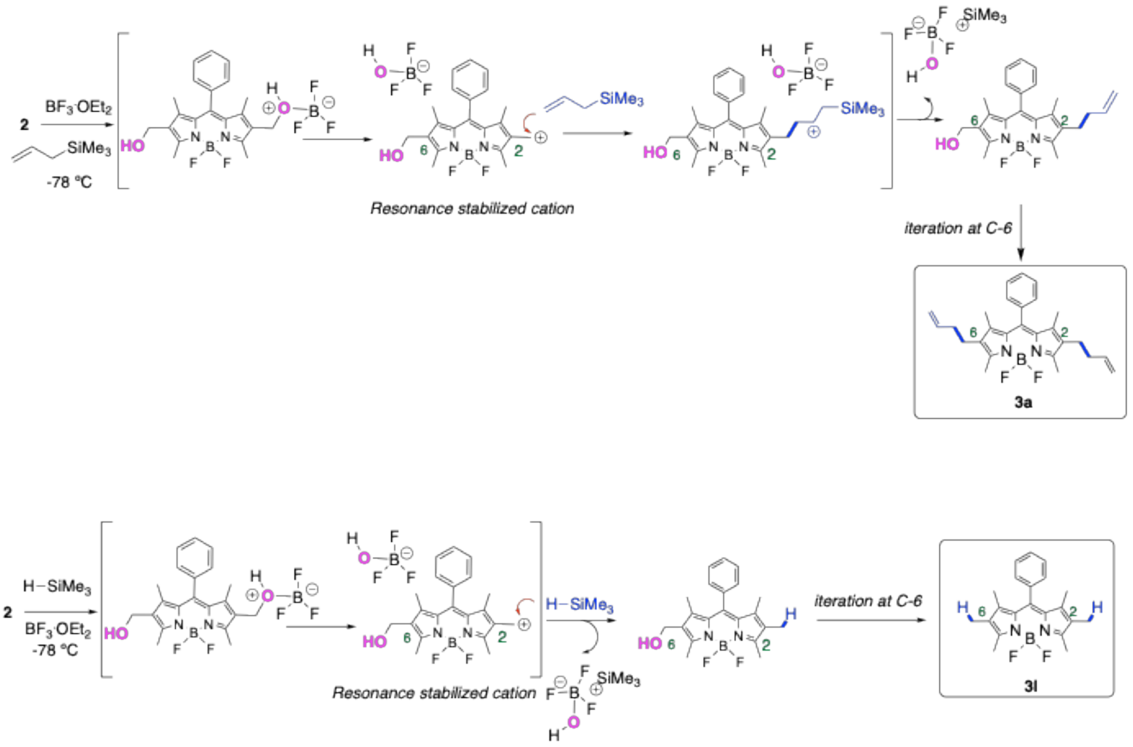

Scheme S4. Plausible mechanism for the formation of compounds **3a** and **3l**.

7. Tables S1-S3. Photophysical data

Table S1. Photophysical properties of 2,6-functionalized BODIPYs in diluted solutions (2 μM).

	λ_{ab} (nm)	ε_{max} (10^4 $M^{-1}\cdot cm^{-1}$)	λ_{fl} (nm)	ϕ	τ (ns)	k_{fl} (10^8 s^{-1})	k_{nr} (10^8 s^{-1})
**3c** (C-Nu)							
Tol	522.0	10.0	537.5	0.74	4.41	1.68	0.59
$CHCl_3$	522.0	8.8	535.0	0.72	4.85	1.48	0.57
MeCN	518.5	8.6	532.0	0.64	4.87	1.31	0.74
**3h** (S-Nu)							
Tol	526.5	8.3	547.0	0.71	3.98	1.78	0.73
$CHCl_3$	525.0	8.6	543.0	0.68	4.28	1.59	0.75
MeCN	520.5	7.8	539.5	0.52	3.69	1.41	1.30
**3l** (H-Nu)							
Tol	527.0	6.8	542.0	0.75	5.05	1.48	0.49
$CHCl_3$	526.5	6.3	541.5	0.73	5.65	1.29	0.44
MeCN	521.0	6.1	535.0	0.68	5.70	1.19	0.56
**3m** (N-Nu)							
Tol	512.5	10.2	525.0	0.68	4.03	1.69	0.79
$CHCl_3$	511.0	10.0	523.0	0.70	4.19	1.67	0.71
MeCN	506.0	9.6	519.5	0.59	3.97	1.48	1.03
**3n** (O-Nu)							
Tol	512.5	6.2	526.5	0.77	3.60	2.13	0.64
$CHCl_3$	511.0	5.6	522.0	0.76	3.96	1.92	0.61
MeCN	506.5	5.9	519.0	0.67	4.06	1.65	0.81

Absorption (λ_{ab}) and fluorescence (λ_{fl}) wavelength, molar absorption at the maximum (ε_{max}), fluorescence quantum yield (ϕ) and fluorescence lifetime (τ), radiative (k_{fl}) and non-radiative (k_{nr}) rate constants.
Tol: toluene; $CHCl_3$: chloroform; MeCN: acetonitrile

Table S2. Photophysical properties of isomeric and triazole-bearing BODIPYs in diluted solutions (2 μM) of ethyl acetate. Previously reported data are in grey.

	λ_{ab} (nm)	$\varepsilon_{max}\cdot 10^{-4}$ (M^{-1} cm^{-1})	λ_{fl} (nm)	ϕ	τ (ns)
**7a**	506.5	7.6	518.0	0.67	3.66
**7b**	506.0	8.0	516.0	0.74	3.76
**9a**	519.0	6.5	532.0	0.79	4.74
**9b**	519.0	9.3	532.5	0.81	5.03

Table S3. Photophysical properties of all-BODIPY based trimer **3f** and BOPHY-BODIPY trimer **3g** in diluted solutions (2 μM)

		λ_{ab} (nm)	ε_{max} (10^4 $M^{-1}\cdot cm^{-1}$)	λ_{fl} (nm)	ϕ	τ (ns)
**3f**	Tol	548.5	18.2	561.0	0.85	3.66
	$CHCl_3$	547.5	15.9	560.0	0.77	3.93
	MeCN	544.5	15.7	558.5	0.02	0.06
**3g**	Tol	534.0	16.5	549.5	0.79	3.71
		462.5	10.9			
	$CHCl_3$	533.0	17.2	546.0	0.76	4.06
		461.0	11.7			
	MeCN	529.5	16.1	544.5	0.06	0.42
		453.5	11.4			

8. Figures S1-S3. Absorption, fluorescence and laser spectra

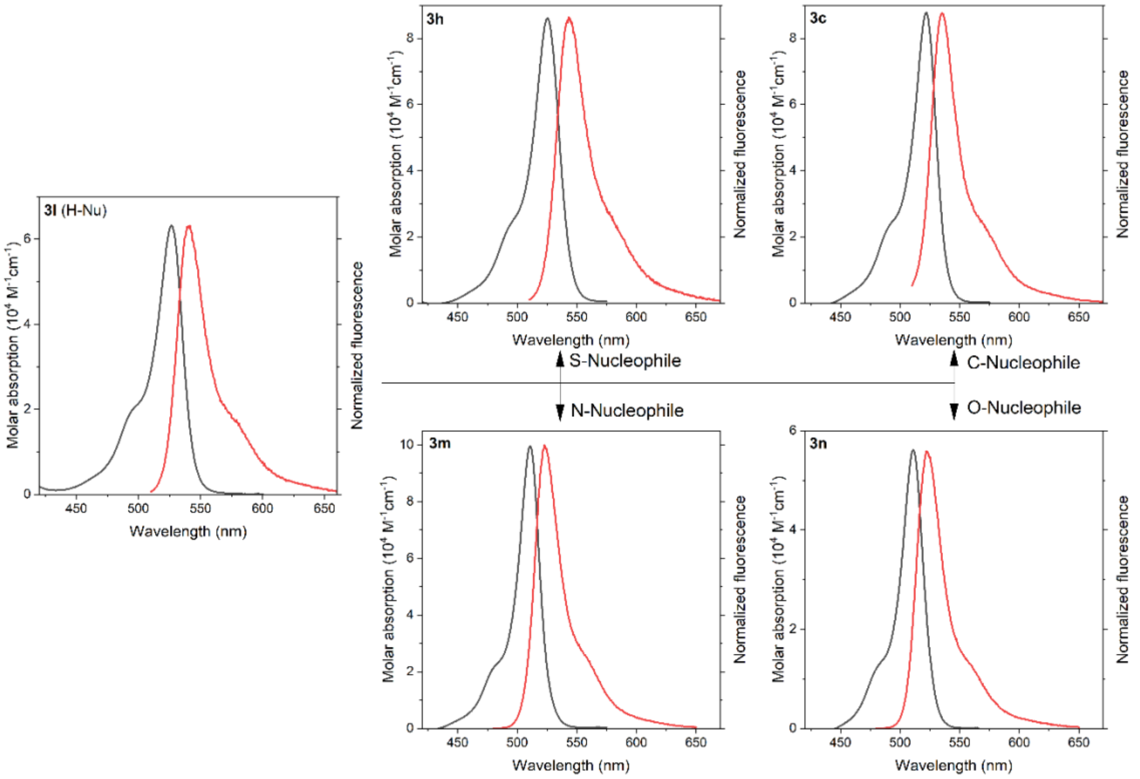

Figure S1. Absorption and normalized fluorescence spectra of the parent alkylated 8-phenyl BODIPY and its corresponding derivatives after addition of different nucleophiles at the 2,6-methyls in diluted solutions (2 μM) of chloroform.

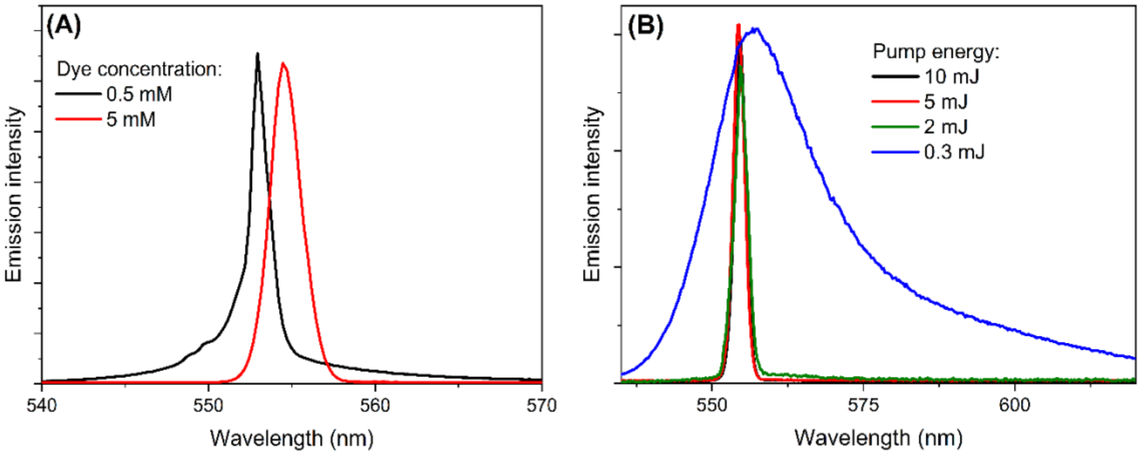

Figure S2. Evolution of the laser induced fluorescence and laser emission of **7a** as a function of the dye concentration (A, pump energy 5 mJ) and pump energy (B, dye concentration 2 mM).

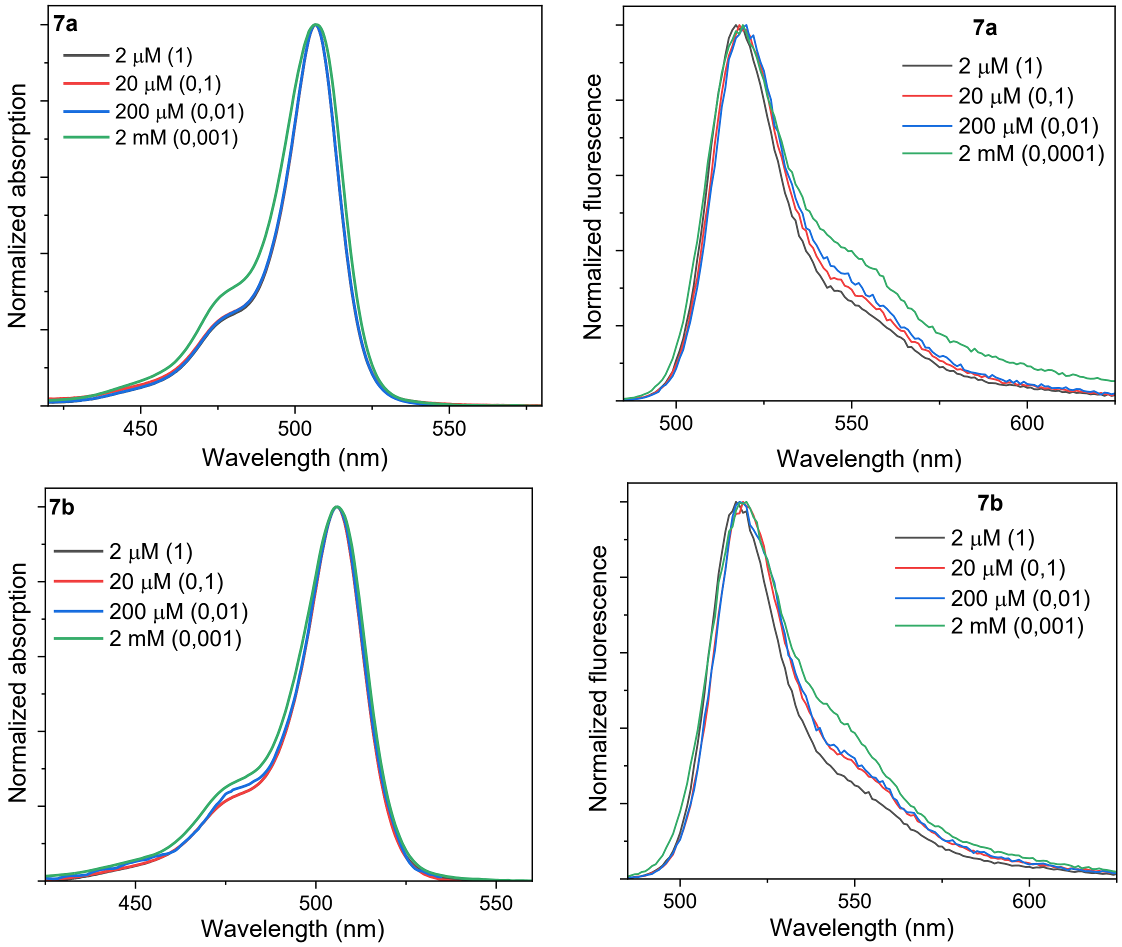

Figure S3. Normalized absorption and fluorescence spectra at different dye concentrations in ethyl acetate. Optical path length is indicated between brackets in cm. It was adjusted to match the optical density in each concentration.

9. *Figure S4 and Videos S1-S2. Molecular dynamics*

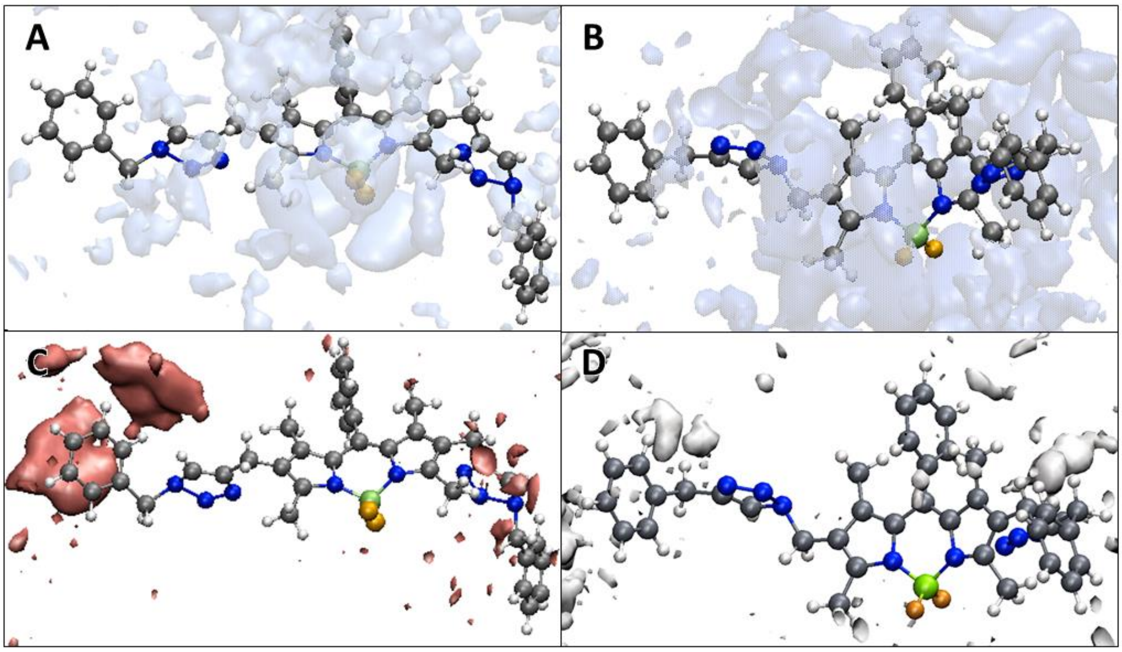

Figure S4. Spatial distribution functions (SDFs) of ethanol molecules around **9a** (A) and **7a** (B), represented as transparent blue isosurfaces. Panels (C) and (D) show the SDFs of **9a** and **7a** molecules around themselves, represented as red and white isosurfaces, respectively.

https://ehubox.ehu.eus/s/QcddyGfgYeJQNC9

Video S1. Last nanosecond trajectory for the conducted molecular dynamics simulation of **9a** in ethanol. The video focuses on two selected **9a** molecules, and ethanol molecules have been omitted for clarity.

https://ehubox.ehu.eus/s/XsfA2ZCHDAoMwbc

Video S2. Last nanosecond trajectory for the conducted molecular dynamics simulation of **7a** in ethanol. The video focuses on two selected **7a** molecules, and ethanol molecules have been omitted for clarity.

10. Figure S5. X-ray crystalline molecular packing

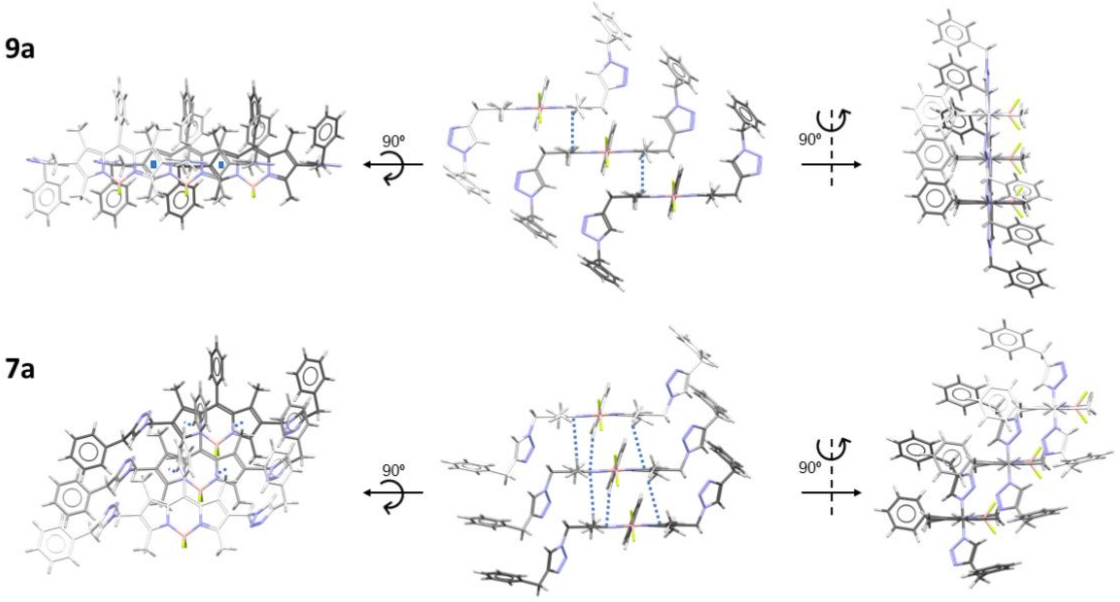

Figure S5. Crystalline molecular packing provided by X-ray diffraction in different views for isomeric BODIPYs **7a** and **9a**.

11. Figures S6-S8. Quantum mechanics simulations and absorption and fluorescence spectra of trimers

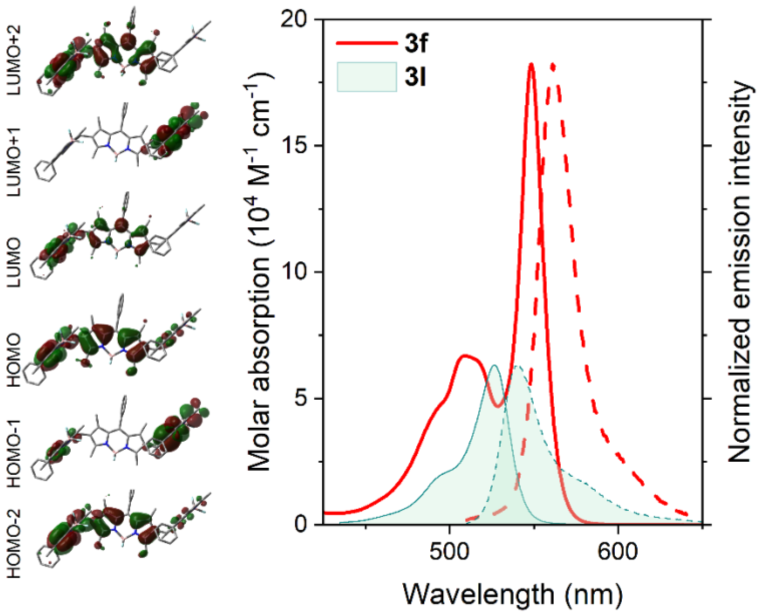

Figure S6. Absorption and normalized fluorescence (dashed) spectra of trimer **3f** in diluted solutions of toluene. For comparison, the corresponding spectra of the single BODIPY precursor **3l** (green shaded) are also included. The molecular orbital (CAM-B3LYP/6-311G*) involved in the absorption are also enclosed

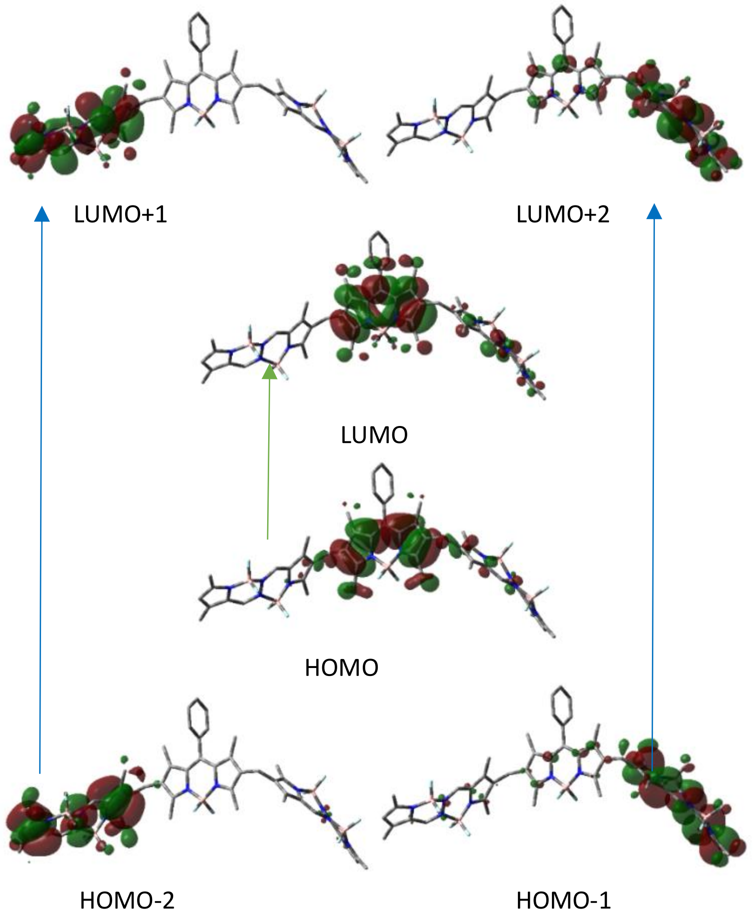

Figure S7. Computed molecular orbitals (CAM-B3LYP/6-311G*) involved in the absorption transition of trimer **3g**.

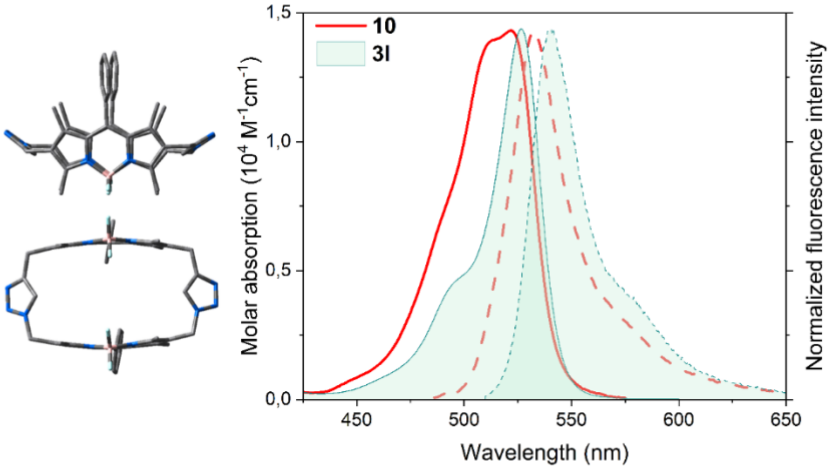

Figure S8. Absorption and normalized fluorescence (dashed) spectra of cyclized dimer **10** in diluted solutions of DMSO. For comparison, the corresponding spectra of the single BODIPY precursor **3l** (green shaded) are also included. The ground state optimized geometry (CAM-B3LYP/6-311G*) in different views, for the cyclized dimer **10** are also enclosed.

12. Table S4 and Figure S9. X-ray diffraction data

Table S4. c1, c2 and c3 are BODIPY centroids of contiguous stacked rings in the crystal structures. pl1, pl2 and pl3 are average planes of the atoms that make up the BODIPY whose centroids are c1, c2 and c3 respectively. c1' and c2' are normal projections of c1 onto the pl2 plane and c2 onto the pl3 plane respectively. cpy are the centroids of the pyrrole rings and cpy' are their normal projections onto the plane of the contiguous BODIPY. All the distances are in Å.

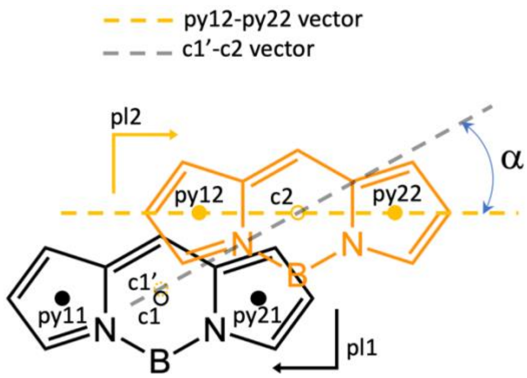

	7a	9a[17]
d(c1…c2)	5.565	6.299
d(c2…c3)	5.565	6.299
d(c1…cpy12)	5.515	4.660
d(c2…cpy13)	5.515	4.660
d(cpy21…cpy12)	5.565	3.656
d(cpy22…cpy13)	5.565	3.656
d(c1…pl2) = d(pl1…pl2) when parallel	4.155	3.551
d(c2…pl3)	4.155	3.551
d(c21…pl2)	4.161	3.556
d(c22…pl3)	4.161	3.556
d(c2…c1')	3.702	5.203
d(c3…c2')	3.702	5.203
d(c1'…cpy12)	3.626	3.018
d(c2'…cpy13)	3.626	3.018
d(cpy21'…cpy12)	3.679	0.850
d(cpy22'…cpy13)	3.679	0.850
α angle (º)	72	1

[17] Gómez, A. M.; Infantes, L.; Ticona-Chambi, J.; Duque-Redondo, E.; Gartzia-Rivero, L.; Uriel, C.; Bañuelos, J.; García-Moreno, I.; López, J. C. Naturally J-aggregated F-BODIPYs: Self-assembly organization driven by substitution pattern. *J. Mol. Liq.* **2024**, *394*, 123773.

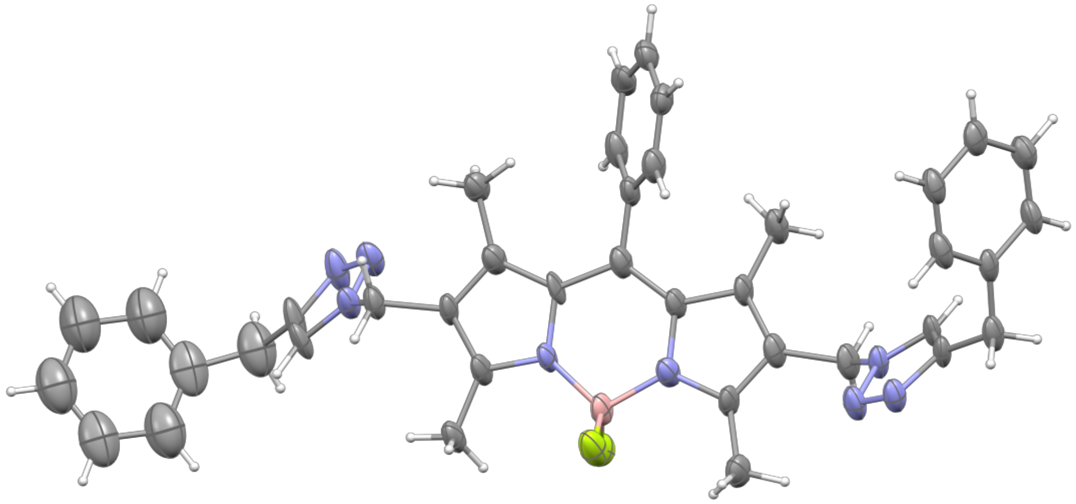

Figure S9. The molecular structure of the compound **7a** showing displacement ellipsoids at the 30% probability level for non-H atoms and fixed-size spheres of radius 0.1 angstrom for hydrogen atoms. Compound **7a** crystallizes in the monoclinic space group $P2_1/c$, with unit-cell parameters a = 24.5613(2) Å, b = 5.56480(10) Å, c = 25.9876(2) Å, β = 108.158(6)°. Structure refinement converged to R_1 = 0.1135, wR_2 = 0.3440, with 5947 reflections (GOF = 1.071).

13. Cartesian coordinates of optimized geometries

Table S5. Atom coordinates and total energy (in hartrees) of ground state optimized geometries (CAM-B3LYP/6-311G*) for methylene-bridged multichromophoric architectures; the all-BODIPY trimer (**3f**), the hybrid bis-(BOPHY)-BODIPY trimer (**3g**) and the cyclic BODIPY dimer (**10**). No negative frequencies were attained for these optimized geometries.

**3f**	C	-1.47208300	-2.70239700	-0.57110400	**3g**	C	-0.96296800	2.14714800	-0.93249600
	N	-0.97079700	-2.28083100	-1.79851100		N	-0.89337200	0.76398800	-0.78926800
	B	0.52239700	-2.16150500	-2.17155400		B	0.17762800	-0.00847800	0.01963500
	N	1.32632000	-2.49828600	-0.89091200		N	1.14156300	1.05390100	0.60256900
	C	0.78304500	-2.89314600	0.32882700		C	1.04783000	2.42949800	0.41275000
	C	-0.59882600	-3.00630700	0.47854500		C	0.00230500	2.96514400	-0.34204800
	C	-2.89207900	-2.70798900	-0.66628200		C	-2.11852400	2.43979300	-1.71858000
	C	-3.20926500	-2.26477500	-1.94670200		C	-2.71273900	1.22133800	-2.01984900
	C	-1.99452000	-2.02544400	-2.62023300		C	-1.92119200	0.20917900	-1.43075600
	C	2.65136000	-2.43587600	-0.77524700		C	2.21827000	0.79064100	1.34225700
	C	3.02952300	-2.78002400	0.54380400		C	2.87862600	1.99811700	1.66348100
	C	1.86678800	-3.07685100	1.24165800		C	2.14969500	3.03275200	1.09064800
	C	3.53012700	-2.04483600	-1.91123700		C	2.60533200	-0.59669300	1.71697200
	C	1.78948500	-3.50487800	2.67326400		C	2.48024400	4.48947100	1.18180400
	C	-1.76516700	-1.56415000	-4.01832800		C	-2.14367400	-1.26207000	-1.46029600
	C	-3.90215700	-3.10683700	0.36206100		C	-2.61562300	3.78441900	-2.14719000
	F	0.81599100	-0.86430500	-2.58788200		F	0.88441700	-0.87689400	-0.81036100
	F	0.84076600	-3.06531900	-3.18904500		F	-0.43622100	-0.72468200	1.04524900
	C	-1.15939600	-3.44634700	1.78590300		C	-0.08425300	4.44145100	-0.52203800
	C	-1.24679300	-4.80131700	2.09004800		C	0.50406300	5.04935900	-1.62648200
	C	-1.78062900	-5.21318700	3.30253800		C	0.42630000	6.42469100	-1.79303500
	C	-2.22468900	-4.27381900	4.22329500		C	-0.24292100	7.20291900	-0.85838900
	C	-2.13601300	-2.92116600	3.92532400		C	-0.83268700	6.60054200	0.24432200
	C	-1.60870600	-2.50893200	2.70990900		C	-0.75238400	5.22545700	0.41275000
	C	4.45107800	-2.81015900	1.04257000		C	4.12998100	2.08985700	2.49610100
	C	5.17832000	-1.49296000	0.95836200		C	5.33260600	1.37095300	1.93996900
	C	-4.59379400	-2.07342300	-2.51197800		C	-3.95759900	0.96293600	-2.82675400
	C	-5.43264600	-1.04475400	-1.79666800		C	-5.08509600	0.31831400	-2.06251100
	C	6.61563300	0.18665700	0.49173900		C	7.12578600	0.04159100	1.71492800
	N	5.62832800	0.67290500	1.34601200		N	6.94017800	0.71618800	0.52041100
	B	5.53221000	2.12290200	1.88792600		B	7.97256300	0.64021300	-0.60945000
	N	6.70397900	2.90643700	1.25672900		N	8.76224400	-0.72394800	-0.36860700
	C	7.67172000	2.37732000	0.40440500		N	8.99064500	-1.22609000	0.90588800

	C	7.62407100	1.03127600	0.03160300		C	8.16978600	-0.85633600	1.86308500
	C	6.33463400	-1.19335100	0.25194200		C	6.12490600	0.44719600	2.61875200
	C	4.77829600	-0.31223300	1.62860400		C	5.88359400	1.52777400	0.65079100
	C	6.96201900	4.20264300	1.45375800		C	5.46325100	2.43811900	-0.45077200
	C	8.11015000	4.55365700	0.72915100		C	5.95748800	-0.03066300	4.02442100
	C	8.57035000	3.42971900	0.06594300		F	8.85877100	1.69628000	-0.55140700
	C	6.11459600	5.06755100	2.31844900		F	7.37966500	0.54749300	-1.85722000
	C	9.77828200	3.40546900	-0.81538900		N	-8.74867600	-1.94110200	-0.33144600
	C	3.60154100	-0.11284400	2.51798000		N	-8.58675400	-0.96221400	0.63988000
	C	7.12307200	-2.14782400	-0.58823900		C	-7.62033400	-0.08924200	0.47207800
	F	5.65104700	2.12473700	3.27954300		C	-6.71085500	-0.09645600	-0.57396200
	F	4.31373900	2.68839900	1.51928300		N	-6.70697200	-1.12633700	-1.49818500
	C	8.67366600	0.50022400	-0.88227300		B	-7.55801400	-2.38326200	-1.29858000
	C	8.47911100	0.51659200	-2.25957100		C	-5.70140000	0.82108800	-0.91497600
	C	9.45842300	0.02481300	-3.11082200		C	-5.72920400	-0.89239000	-2.38582400
	C	10.63956400	-0.48726500	-2.59182200		C	-5.37632800	2.08888100	-0.19722000
	C	10.83850800	-0.50427700	-1.21814900		C	-5.44492300	-1.85742600	-3.48521400
	C	9.86007300	-0.01144000	-0.36655600		C	-9.92122600	-2.52705600	-0.41655300
	C	-9.17617200	3.38951600	1.59523200		C	-10.99675400	-2.28442200	0.42177200
	C	-7.55703900	2.48281500	0.33334600		N	-10.86586700	-1.44195900	1.51594300
	N	-8.49722200	1.49706600	0.63146900		B	-9.49655300	-0.91148100	1.95150900
	B	-8.42307600	0.01738900	0.19039500		C	-12.32063500	-2.76430200	0.37570500
	N	-7.10356000	-0.14066400	-0.60103200		C	-12.96601000	-2.19027100	1.46222200
	C	-6.20221800	0.87863300	-0.89664300		C	-12.04233300	-1.39189700	2.15354800
	C	-6.42976500	2.17503700	-0.43012400		F	-8.14356700	-2.81567800	-2.47876200
	C	-7.98903500	3.69198400	0.95380400		F	-6.83578200	-3.39288500	-0.69861000
	C	-9.46446200	2.03302500	1.37985200		F	-9.55202400	0.41383900	2.35144700
	C	-6.65584000	-1.28117600	-1.13419900		F	-8.93267400	-1.70863800	2.92415300
	C	-5.14932100	0.31039800	-1.66929000		C	-12.90947700	-3.66639300	-0.65854000
	C	-7.41835400	-2.55282300	-0.98821100		C	-12.23858500	-0.61310000	3.40678500
	C	-3.95599100	0.98482400	-2.26689700		C	9.24861900	-1.39881200	-1.38516300
	C	-10.62687000	1.24667000	1.87390000		C	10.02253200	-2.54313800	-1.28401900
	C	-7.34478300	5.04135000	0.96255500		N	10.43783700	-3.00987500	-0.04539100
	F	-9.51614700	-0.29685600	-0.61941900		B	10.22794600	-2.18094300	1.22598500
	F	-8.41348900	-0.81716500	1.30937900		C	10.51239200	-3.40910100	-2.28160100
	C	-5.45032100	3.25236500	-0.74016700		C	11.22365200	-4.39277400	-1.60860900
	C	-4.29379700	3.39081500	0.02018500		C	11.17169000	-4.11375800	-0.23450600
	C	-3.37986000	4.39305200	-0.27191500		C	10.27002300	-3.30743100	-3.75164300
	C	-3.61192300	5.26004200	-1.33064000		C	11.81723700	-4.84587400	0.88933400
	C	-4.76387800	5.12438100	-2.09343300		F	9.86318900	-2.96529700	2.30814100
	C	-5.68195300	4.12736800	-1.79700500		F	11.33403700	-1.40909700	1.51245300
	H	3.12611100	-2.41443700	-2.85187400		H	3.14500000	-1.07918600	0.89866900
	H	3.59866500	-0.95696800	-1.98613600		H	1.72219300	-1.19826100	1.92480200
	H	4.53685800	-2.43629700	-1.77540900		H	3.25311000	-0.59552300	2.59196700
	H	2.77074500	-3.47029400	3.14471000		H	2.57069900	4.95342700	0.19914600
	H	1.11890500	-2.87247300	3.25592400		H	3.42203600	4.64349300	1.70689000
	H	1.41874500	-4.52605300	2.77207600		H	1.71290000	5.04792000	1.71985600
	H	-2.68678300	-1.56460900	-4.59735800		H	-2.74828600	-1.54675700	-2.32027700
	H	-1.34421900	-0.55673800	-4.02726800		H	-2.66722100	-1.59029000	-0.55907100
	H	-1.04052600	-2.21049600	-4.51374500		H	-1.19485800	-1.79372900	-1.49968800
	H	-4.12354900	-2.28814700	1.05141400		H	-1.89654500	4.30126700	-2.78380700
	H	-4.84089700	-3.37986200	-0.11848200		H	-2.80755200	4.44098400	-1.29759000
	H	-3.57666800	-3.95356200	0.96223500		H	-3.54328800	3.69422000	-2.71042100
	H	-0.90109900	-5.53332700	1.36833300		H	1.02592400	4.43968700	-2.35611200
	H	-1.85050000	-6.27140500	3.52833900		H	0.88996700	6.88944800	-2.65596200
	H	-2.64168000	-4.59570300	5.17083300		H	-0.30413000	8.27741000	-0.98899600
	H	-2.48350200	-2.18306600	4.63943000		H	-1.35643300	7.20310000	0.97802100
	H	-1.54427500	-1.45266400	2.47299300		H	-1.21038100	4.75353500	1.27528600
	H	4.46276900	-3.16263100	2.07665700		H	3.92553300	1.69258900	3.49500600
	H	5.01046000	-3.56634200	0.48666300		H	4.37844900	3.14113800	2.66130600
	H	-4.51501800	-1.80323700	-3.56818200		H	-3.70324900	0.33825200	-3.68702200
	H	-5.12095100	-3.03088900	-2.50821400		H	-4.30885200	1.90266500	-3.25996700
	H	8.55181500	5.53946700	0.69961700		H	8.36935100	-1.32729800	2.81615400
	H	6.05997400	4.66195000	3.32990900		H	4.72736200	3.15852300	-0.10229500
	H	6.51629600	6.07922400	2.36050700		H	5.02503300	1.87464000	-1.27684400
	H	5.09196800	5.10456800	1.93958600		H	6.32384700	2.97733800	-0.84996400
	H	10.22001100	4.40153600	-0.86189000		H	6.74888900	-0.71988900	4.31914500
	H	10.54163500	2.71730500	-0.44952100		H	5.00764800	-0.55368200	4.16300000
	H	9.53994700	3.09627500	-1.83399900		H	5.97246400	0.80209700	4.73098300
	H	2.77072200	0.32501500	1.95955500		H	-7.58279300	0.67488400	1.23662500
	H	3.26333600	-1.05937600	2.93527900		H	-4.36957000	2.05391500	0.22354600
	H	3.84742800	0.56985500	3.32931300		H	-6.07329700	2.29052700	0.61654900
	H	8.16430200	-2.20567700	-0.27054000		H	-5.40872100	2.94542300	-0.87490000
	H	6.70859100	-3.15331800	-0.53036900		H	-5.09546400	-2.80939000	-3.07723900
	H	7.13452800	-1.85490400	-1.63943700		H	-4.68572900	-1.47773400	-4.16621300

	H 7.55570900 0.91725300 -2.66343500<br>H 9.29744500 0.04187200 -4.18294500<br>H 11.40431200 -0.87200800 -3.25707100<br>H 11.75948700 -0.90211800 -0.80675000<br>H 10.01409300 -0.02288900 0.70697500<br>H -9.78424400 4.07212000 2.17148700<br>H -8.46321900 -2.39760500 -1.25686000<br>H -7.00847800 -3.34161700 -1.61620100<br>H -7.40880100 -2.88892700 0.05064900<br>H -3.13485900 1.06488300 -1.55005300<br>H -3.58324500 0.40999800 -3.11385000<br>H -4.17632800 1.99054600 -2.61748800<br>H -10.29235400 0.43303400 2.51944600<br>H -11.31349500 1.88408700 2.42961600<br>H -11.15998400 0.78643400 1.04063200<br>H -7.35456700 5.50614100 -0.02474100<br>H -7.88077800 5.70073300 1.64627800<br>H -6.30211700 5.00099800 1.27929300<br>H -4.11107600 2.70844600 0.84291000<br>H -2.48074700 4.49283500 0.32562900<br>H -2.89449100 6.03907900 -1.56233600<br>H -4.94915800 5.79721600 -2.92328300<br>H -6.58193400 4.01867700 -2.39228800<br><br>E = -3284.444633		H -6.34833000 -2.07188800 -4.05573500<br>H -10.00041600 -3.23919400 -1.22728500<br>H -13.99974500 -2.32982800 1.74416800<br>H -13.78820200 -4.18078900 -0.26879200<br>H -12.20129400 -4.42800500 -0.99026700<br>H -13.22367400 -3.10524800 -1.54258700<br>H -13.27078900 -0.68833700 3.74631300<br>H -11.98960700 0.43821700 3.25900200<br>H -11.58512300 -0.98638300 4.19873900<br>H 8.98754800 -0.98925700 -2.35204700<br>H 11.73883400 -5.23233800 -2.05279800<br>H 11.04223200 -3.83801500 -4.30919600<br>H 10.26643100 -2.27166600 -4.09645700<br>H 9.30729000 -3.74638500 -4.02647000<br>H 12.34534300 -5.72415200 0.52036100<br>H 11.07968900 -5.16027600 1.62838900<br>H 12.53294100 -4.20343900 1.40760100<br><br>E = -3568.301649
**10**	C 0.40776000 3.26723000 0.24752200<br>C 1.57770000 2.94987600 -0.46216000<br>N 1.50308900 2.59200900 -1.81062500<br>B 0.22834400 2.66982900 -2.69565700<br>N -0.96304100 2.90684400 -1.72589400<br>C -0.85524000 3.19677600 -0.36426100<br>C -2.26523400 2.81914600 -2.05087300<br>C -3.05450200 3.02234800 -0.89268700<br>C -2.18357200 3.27177900 0.16981900<br>C 2.94112800 2.79750400 -0.04776500<br>C 3.63999900 2.30803800 -1.15338800<br>C 2.72535700 2.21790800 -2.23010300<br>C 3.56141800 3.07759300 1.28830200<br>C 2.98720100 1.79765600 -3.63899800<br>C -2.71968700 2.55293500 -3.44833300<br>C -2.61038600 3.52638700 1.58481900<br>F 0.33127400 3.71587400 -3.61688900<br>F 0.04864800 1.45325400 -3.36995200<br>C 0.50099600 3.66500000 1.68417600<br>C 0.65903000 2.70704800 2.68976200<br>C 0.74698600 3.09170900 4.02549100<br>C 0.68278600 4.44023900 4.37119400<br>C 0.52876400 5.40137000 3.37408200<br>C 0.43456400 5.01621200 2.03841800<br>C -4.55243800 2.87416900 -0.82678800<br>C 5.06716800 1.82570100 -1.17097700<br>C 5.19468800 0.39831400 -0.70339000<br>C 4.39315500 -0.68817200 -0.98213300<br>N 4.94238700 -1.70710800 -0.27862300<br>N 6.02635600 -1.28461900 0.40109000<br>N 6.17756200 -0.01371500 0.14616000<br>C -4.40165500 0.27014100 -0.93197500<br>C -4.99117900 1.45794900 -0.55704800<br>N -6.11134000 1.16096600 0.16160500<br>N -6.24381800 -0.13420200 0.24339800<br>N -5.20967900 -0.68902300 -0.41830100<br>C -0.36446600 -2.91742000 1.15776700<br>C -1.56930000 -2.92055200 0.43617300<br>N -1.56757600 -3.19406600 -0.93533300<br>B -0.34398200 -3.71798500 -1.74384600<br>N 0.90197900 -3.48795100 -0.83866000<br>C 0.86400200 -3.15183400 0.51759800<br>C 2.18255800 -3.56328700 -1.23613600<br>C 3.02614700 -3.25348200 -0.14080800<br>C 2.21380700 -3.00484400 0.96867100<br>C -2.90461900 -2.56733300 0.80853600<br>C -3.65864400 -2.59714100 -0.36743000<br>C -2.80602200 -3.00895800 -1.42096500<br>C -3.45593000 -2.20767300 2.15477100		

C	-3.14014300	-3.23445700	-2.85877100
C	2.56092000	-3.92573500	-2.63453600
C	2.71948400	-2.62038500	2.32651500
F	-0.49612800	-5.07614300	-2.02964700
F	-0.20895200	-2.98506100	-2.92546100
C	-0.38197800	-2.62150000	2.62069700
C	-0.47712000	-1.30554100	3.08266400
C	-0.49503900	-1.03818200	4.44925300
C	-0.42339200	-2.08372800	5.36815900
C	-0.33127500	-3.39789900	4.91401900
C	-0.30639900	-3.66629300	3.54694100
C	4.51731400	-3.11379700	-0.19866500
C	-5.08212300	-2.15236700	-0.50911600
H	3.42284900	2.24868900	1.98916700
H	4.63796500	3.22190500	1.18255700
H	3.14941900	3.96863400	1.76067700
H	4.05045900	1.62931500	-3.80899000
H	2.44107700	0.88397100	-3.88465300
H	2.63787800	2.56204900	-4.33704500
H	-2.30207500	3.29490900	-4.13362700
H	-2.36625100	1.58024100	-3.79673800
H	-3.80652800	2.58128400	-3.52202400
H	-2.38717800	4.54798400	1.90347700
H	-3.68415100	3.37196700	1.69550400
H	-2.10965800	2.86393500	2.29331600
H	0.72183100	1.65795700	2.42065200
H	0.87737500	2.33812900	4.79565700
H	0.75606800	4.74057700	5.41122800
H	0.48221800	6.45370700	3.63473900
H	0.31527800	5.76534200	1.26264400
H	-4.97819800	3.50782700	-0.04592400
H	-5.00467200	3.22750900	-1.76021800
H	5.70041200	2.43611700	-0.52310500
H	5.49006200	1.92894700	-2.17583900
H	3.49731600	-0.80266400	-1.56816100
H	-3.49813700	0.04221100	-1.47092800
H	-4.53959900	-2.33559000	2.16961100
H	-3.03332300	-2.81680200	2.95304600
H	-3.26025900	-1.16199700	2.40871700
H	-2.63498600	-2.50531900	-3.49607300
H	-2.79806500	-4.22015500	-3.18102100
H	-4.21389000	-3.16710200	-3.03279100
H	2.22600800	-3.16210300	-3.33955200
H	3.63868900	-4.04975600	-2.73699900
H	2.07394700	-4.85714100	-2.93247500
H	2.51733200	-3.39463800	3.07107700
H	3.79627100	-2.45014600	2.30466300
H	2.25206700	-1.70552900	2.69441900
H	-0.54341600	-0.49287200	2.36689400
H	-0.57497600	-0.01278900	4.79513000
H	-0.44228500	-1.87553900	6.43275500
H	-0.27907200	-4.21701000	5.62371800
H	-0.23591100	-4.68997900	3.19404700
H	5.00776700	-3.50965400	0.69053700
H	4.93049500	-3.65139100	-1.05481400
H	-5.50691300	-2.48084100	-1.45994600
H	-5.72562300	-2.54337000	0.27978100

E = -2776.942960

14. Copies of IR spectra of compounds 3m, 8 and 10

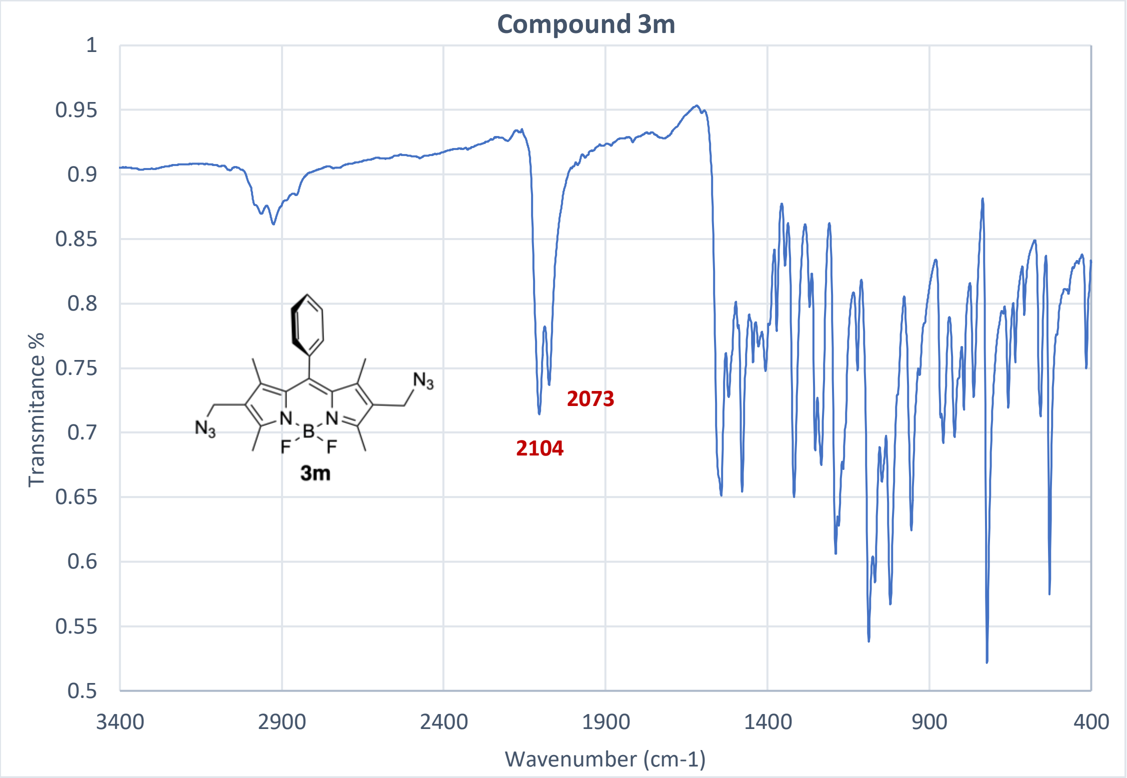

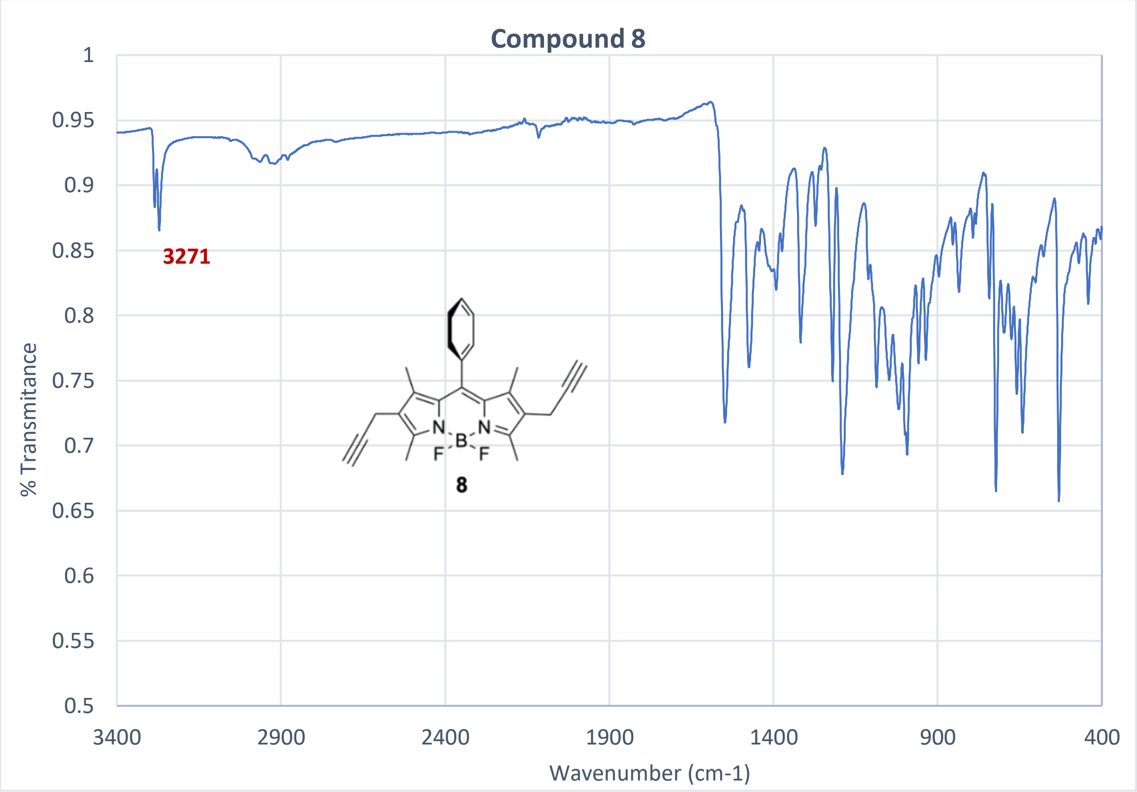

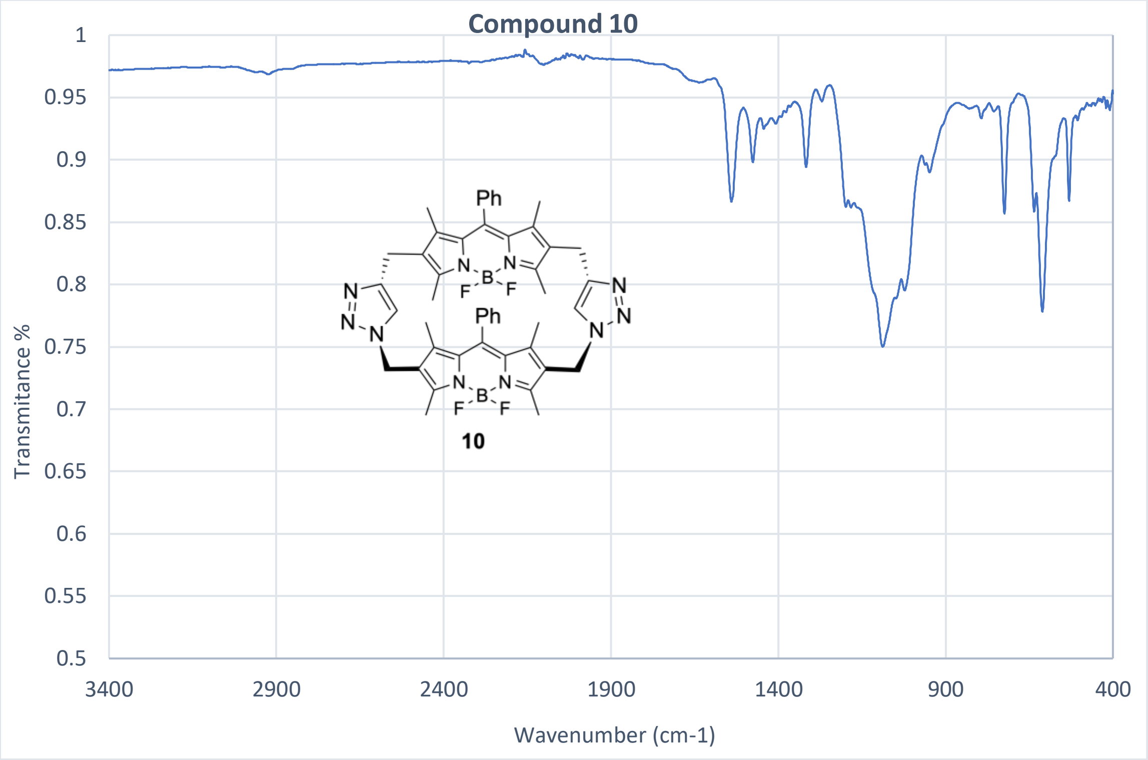

Compound 10
Transmitance %
Wavenumber (cm-1)
1
0.95
0.9
0.85
0.8
0.75
0.7
0.65
0.6
0.55
0.5
3400
2900
2400
1900
1400
900
400
Ph
N
B
F
10

15. HRMS spectrum of compound 10

Qualitative Compound Report

**Data File**	1753_CAGE_2A_01.d	**Sample Name**	CAGE_2A
**Sample Type**	Sample	**Position**	Vial 7
**Instrument Name**	Instrument 1	**User Name**	
**Acq Method**	ESI_ACN_75_pos_new.m	**Acquired Time**	2:30:31 PM (UTC+02:00)
**IRM Calibration Status**	Success	**DA Method**	Defecto_modificado.m
**Comment**			

**Sample Group**			
**User**		**Stream Name**	LC 1
**Acquisition Time (Local)**	2:30:31 PM (UTC+02:00)	**Acquisition SW Version**	6200 series TOF/6500 series Q-TOF B.08.00 (B8058.3 SP1)
**QTOF Driver Version**	8.00.00	**QTOF Firmware Version**	2.712
**Tune Mass Range Max.**	1700		

Compound Table

Compound Label	RT	Mass	Abund	Formula	Tgt Mass	Diff (ppm)	Hits (DB)
Cpd 1: C46 H44 B2 F4 N10; 1.171	1.171	832.3928	69480	C46 H44 B2 F4 N10	832.3945	-2.13	1

Compound Label	*m/z*	RT	Algorithm	Mass
Cpd 1: C46 H44 B2 F4 N10; 1.171	835.3943	1.171	Find by Formula	832.3928

MS Zoomed Spectrum

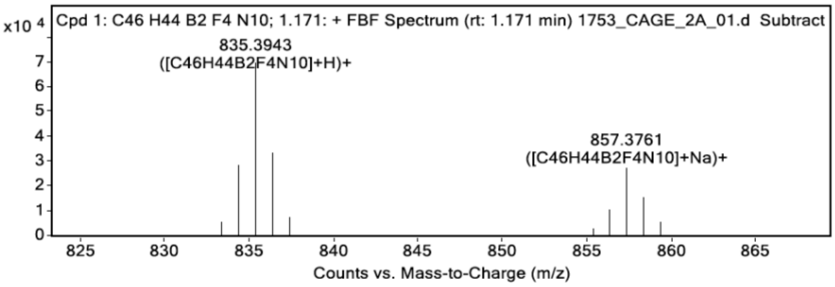

MS Spectrum Peak List

m/z	z	Abund	Formula	Ion
833.399	1	5258.5	C46H44B2F4N10	(M+H)+
834.3982	1	28318.1	C46H44B2F4N10	(M+H)+
835.3943	1	69480.48	C46H44B2F4N10	(M+H)+
836.3972	1	33275.54	C46H44B2F4N10	(M+H)+
837.3987	1	7180.49	C46H44B2F4N10	(M+H)+
855.38	1	2411.87	C46H44B2F4N10	(M+Na)+
856.3767	1	10146.7	C46H44B2F4N10	(M+Na)+
857.3761	1	26963.09	C46H44B2F4N10	(M+Na)+
858.3741	1	15315.92	C46H44B2F4N10	(M+Na)+
859.3889	1	5219.32	C46H44B2F4N10	(M+Na)+

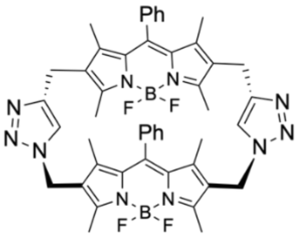

10

MS Zoomed Spectrum

16. Copies of ^{1}H, $^{13}C\ \{^{1}H\}$, ^{19}F, ^{11}B NMR spectra

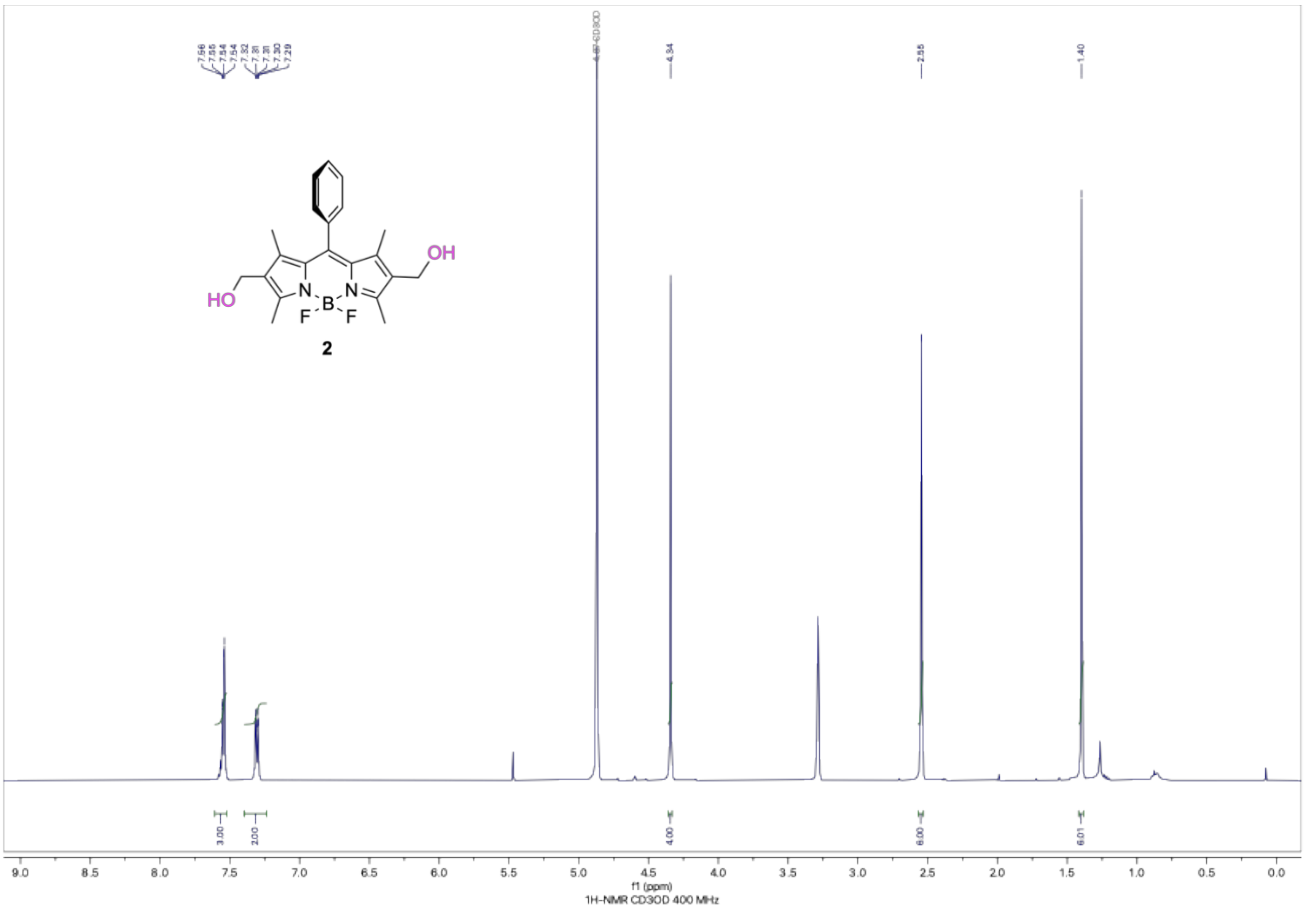

^{1}H-NMR (CD_3OD, 400 MHz) of Compound **2**

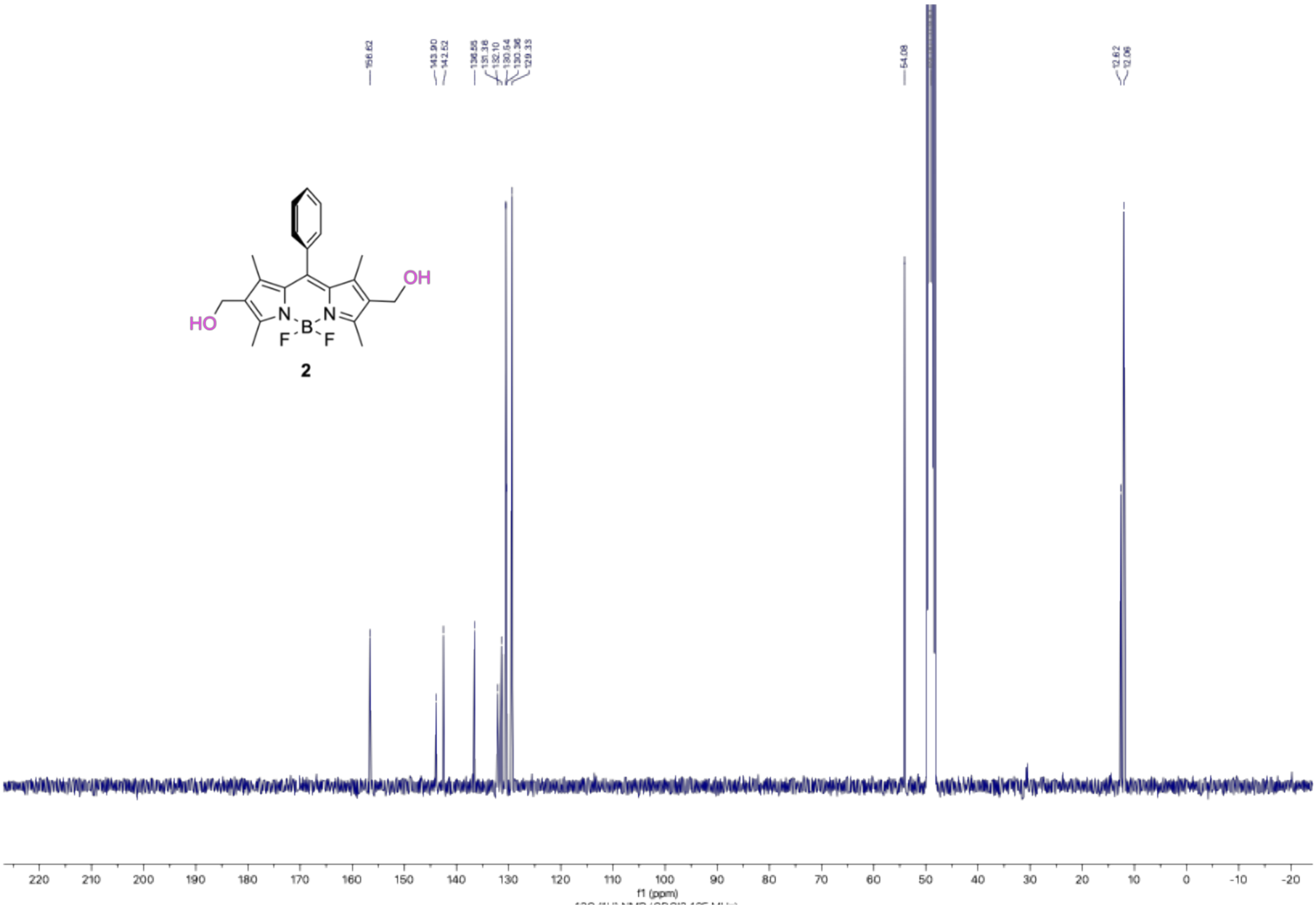

$^{13}C\ \{^{1}H\}$-NMR (CD_3OD, 125 MHz) of Compound **2**

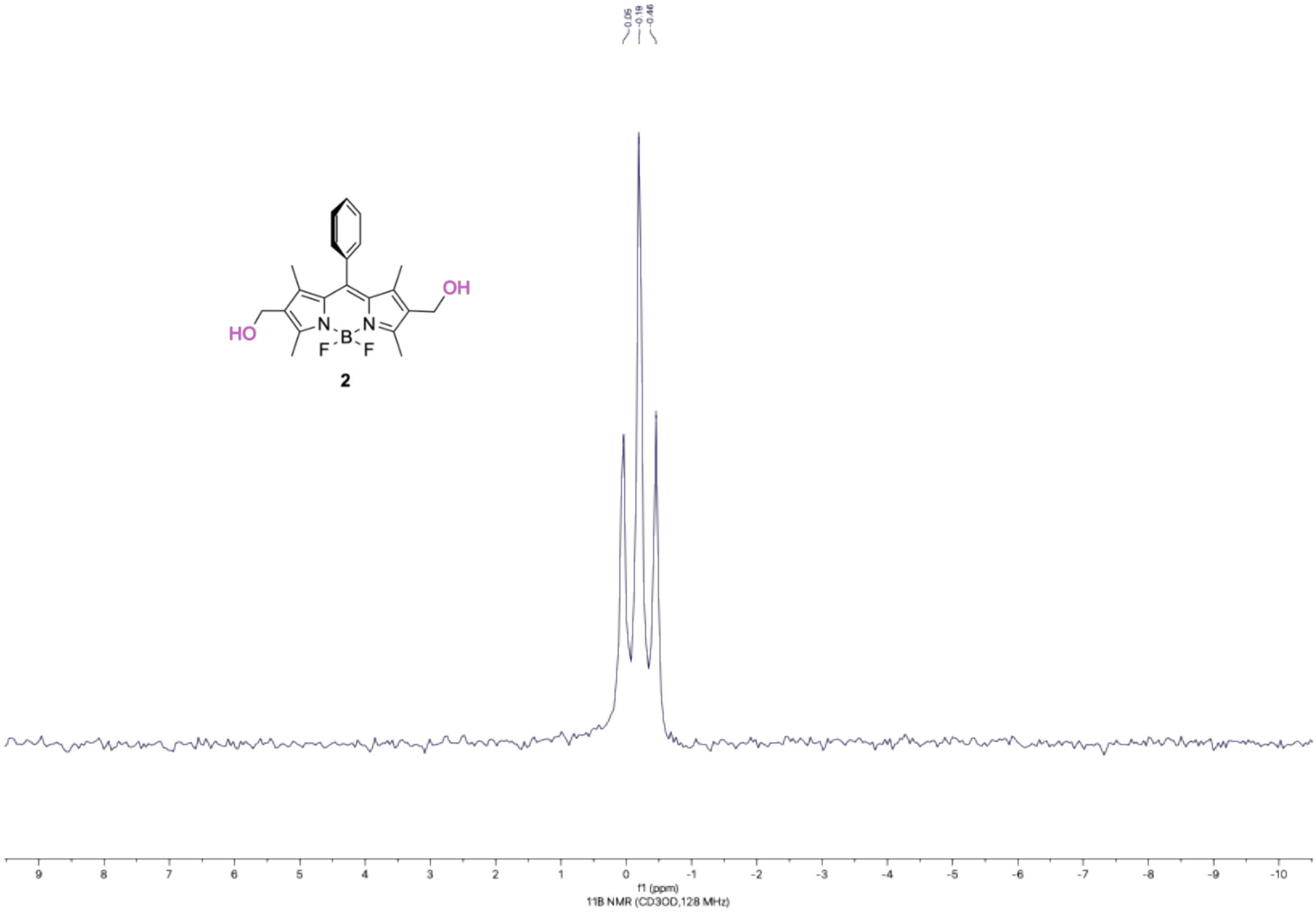

^{11}B-NMR (CD_3OD, 128 MHz) of Compound **2**

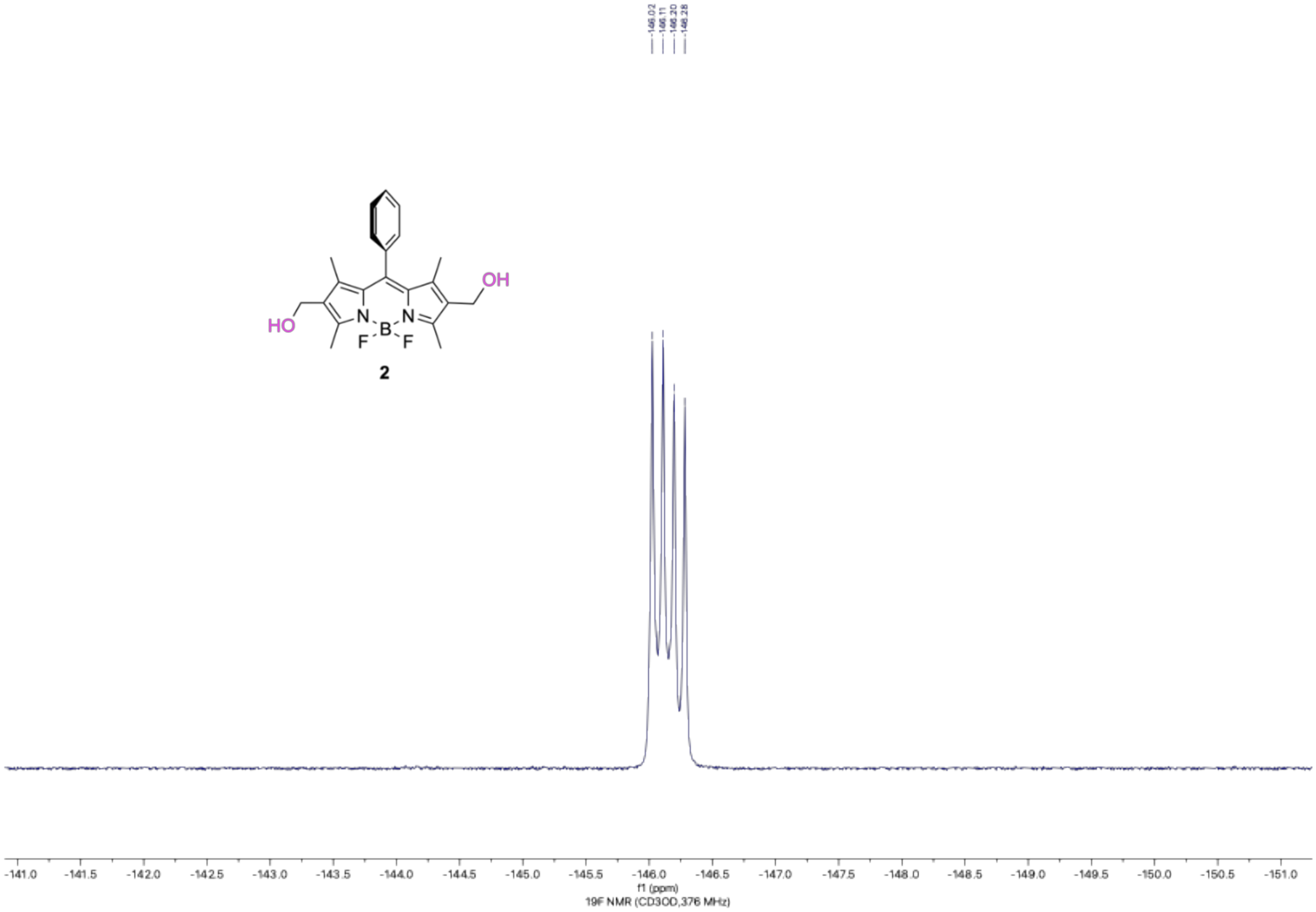

^{19}F-NMR (CD_3OD, 376 MHz) of Compound **2**

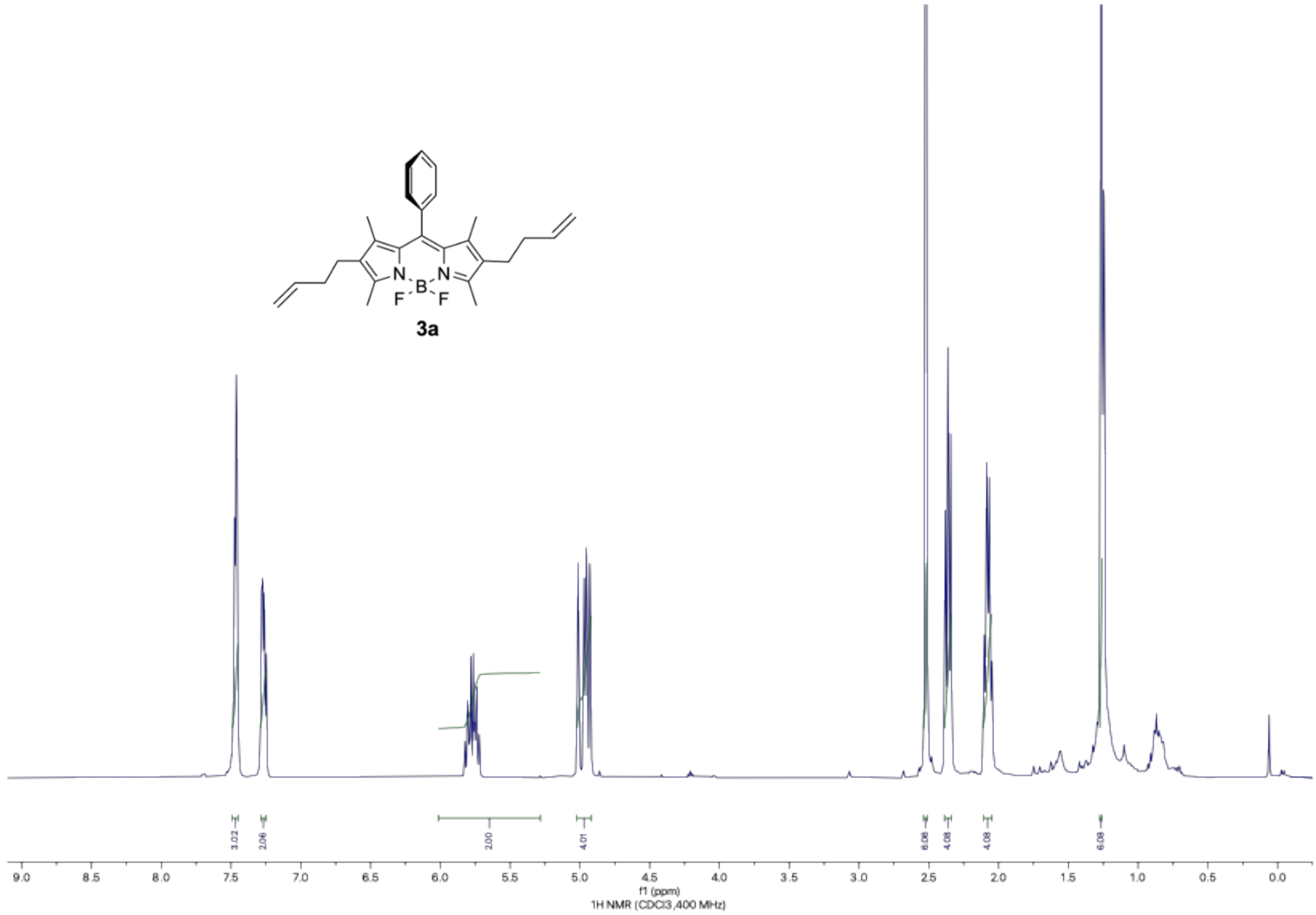

^{1}H-NMR ($CDCl_3$, 400 MHz) of Compound **3a**

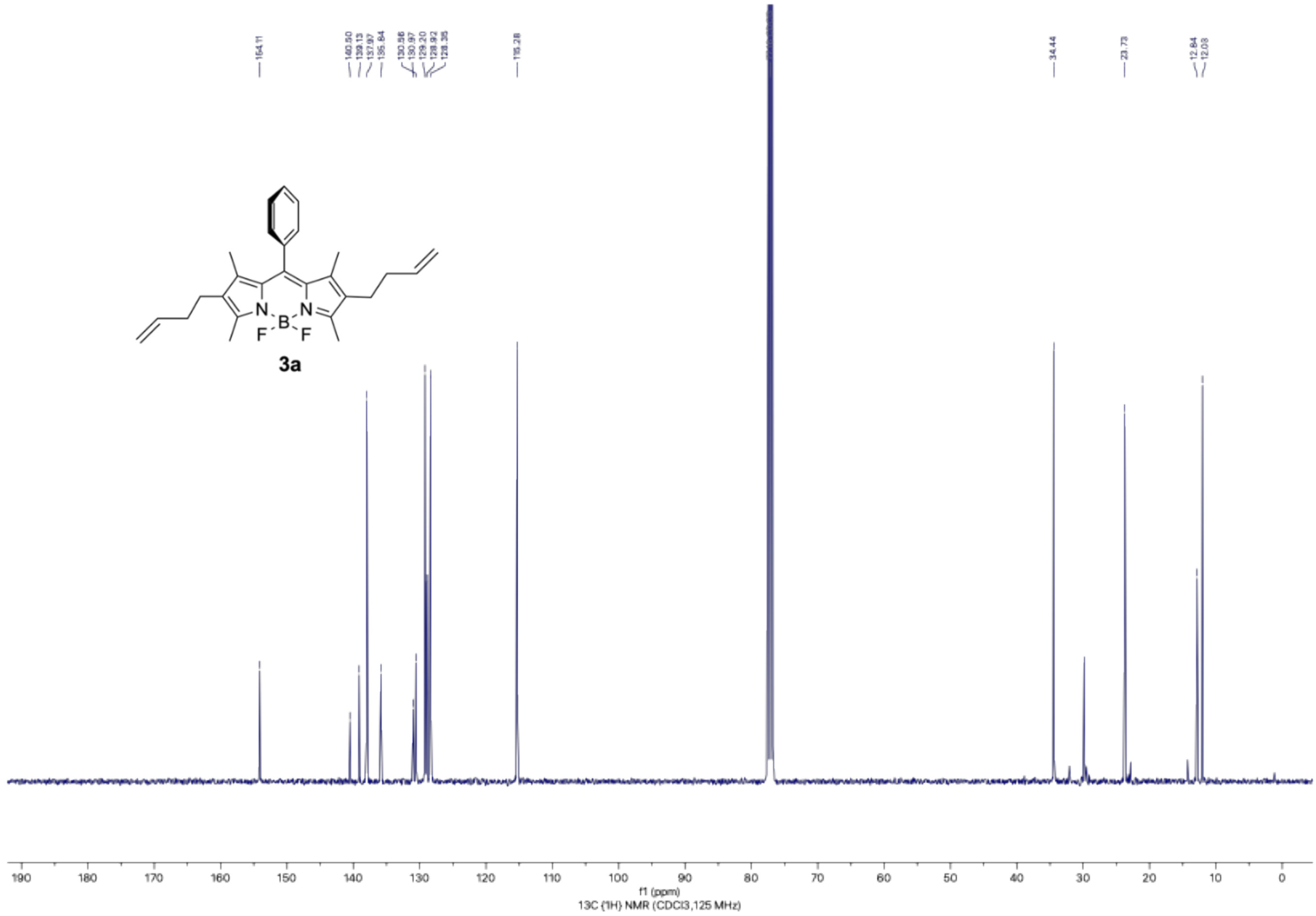

^{13}C {^{1}H}-NMR ($CDCl_3$, 125 MHz) of Compound **3a**

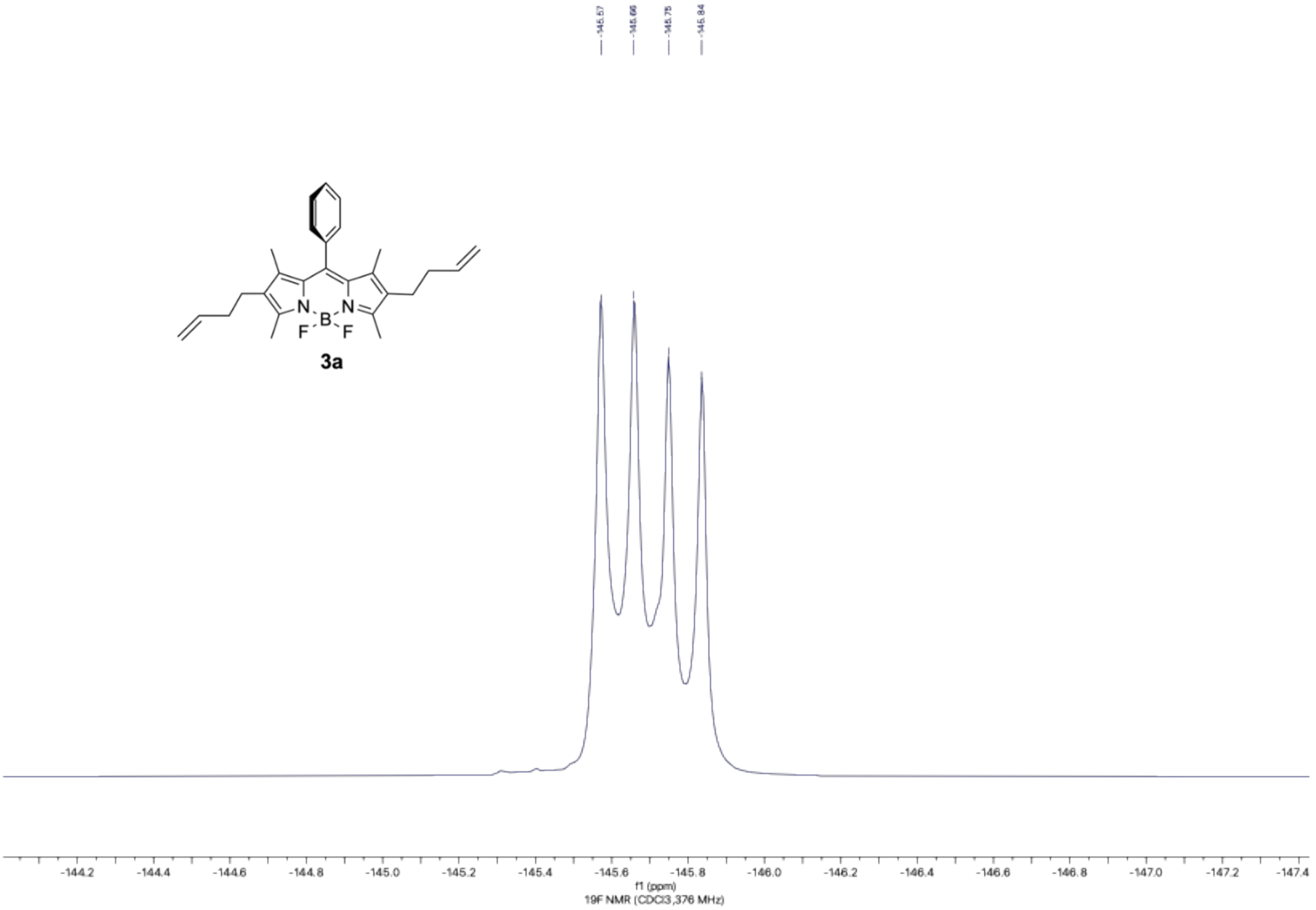

^{19}F-NMR ($CDCl_3$, 376 MHz) of Compound **3a**

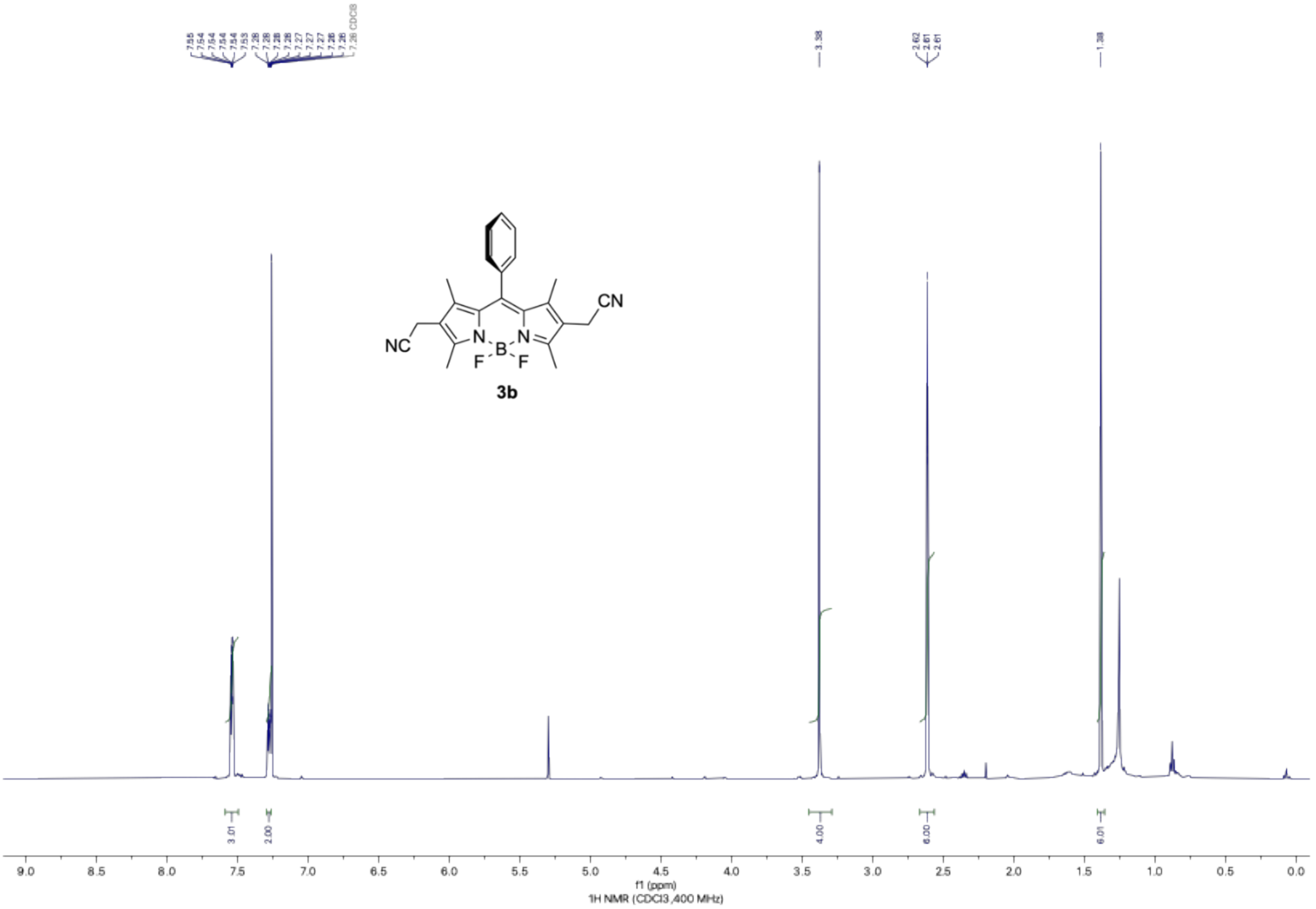

^{1}H-NMR ($CDCl_3$, 400 MHz) of Compound **3b**

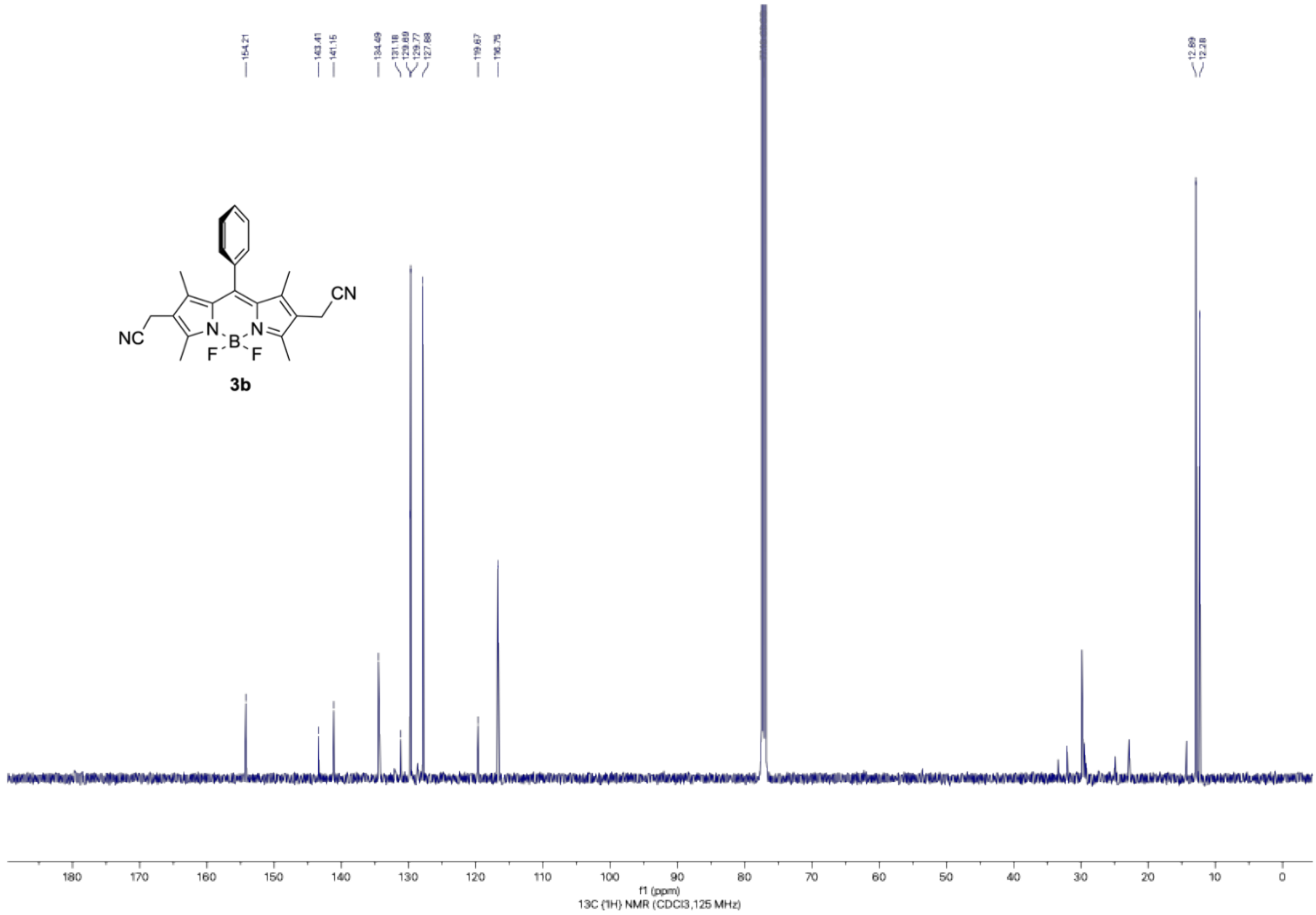

^{13}C {^{1}H}-NMR ($CDCl_3$, 125 MHz) of Compound **3b**

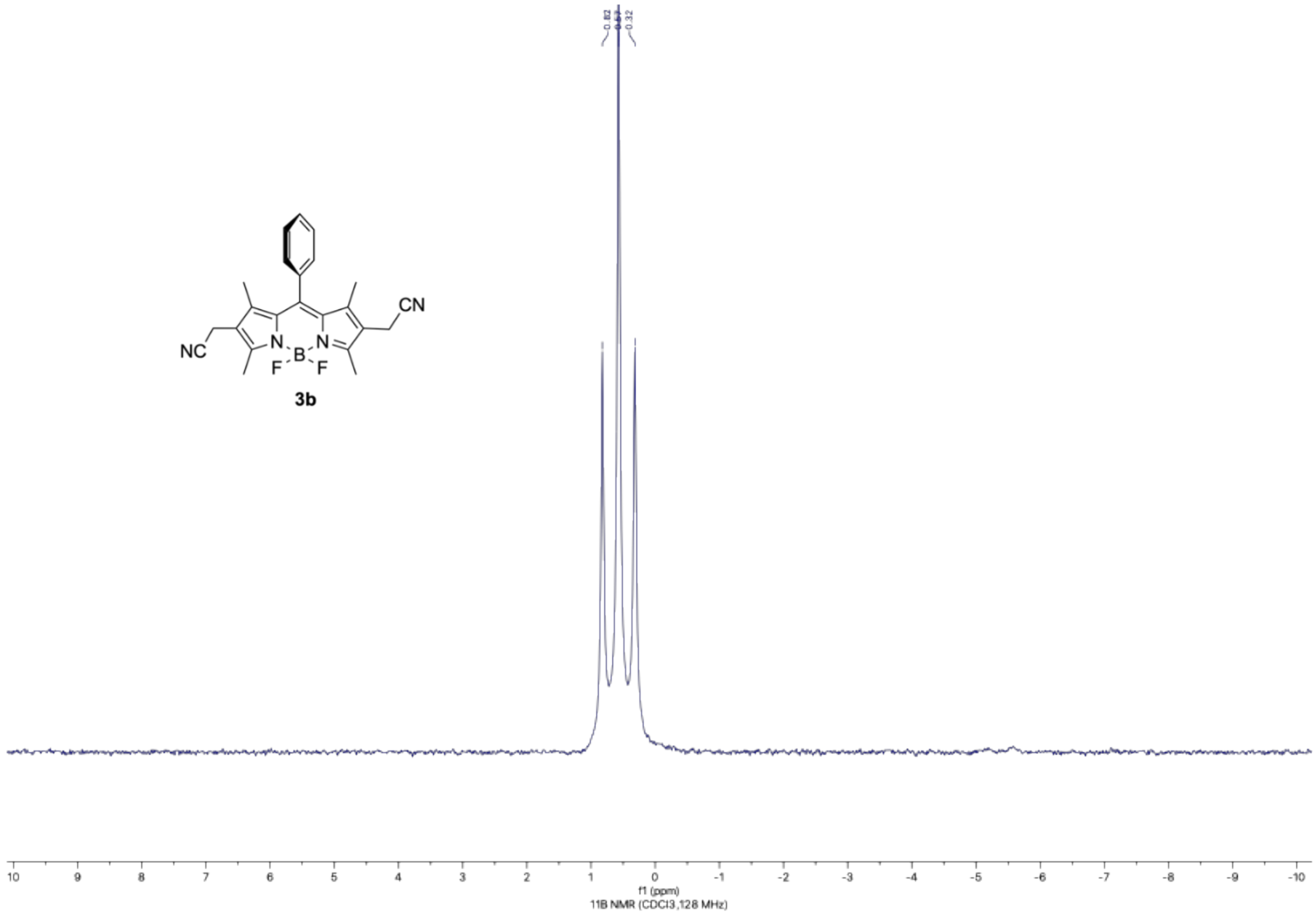

^{11}B-NMR ($CDCl_3$, 128 MHz) of Compound **3b**

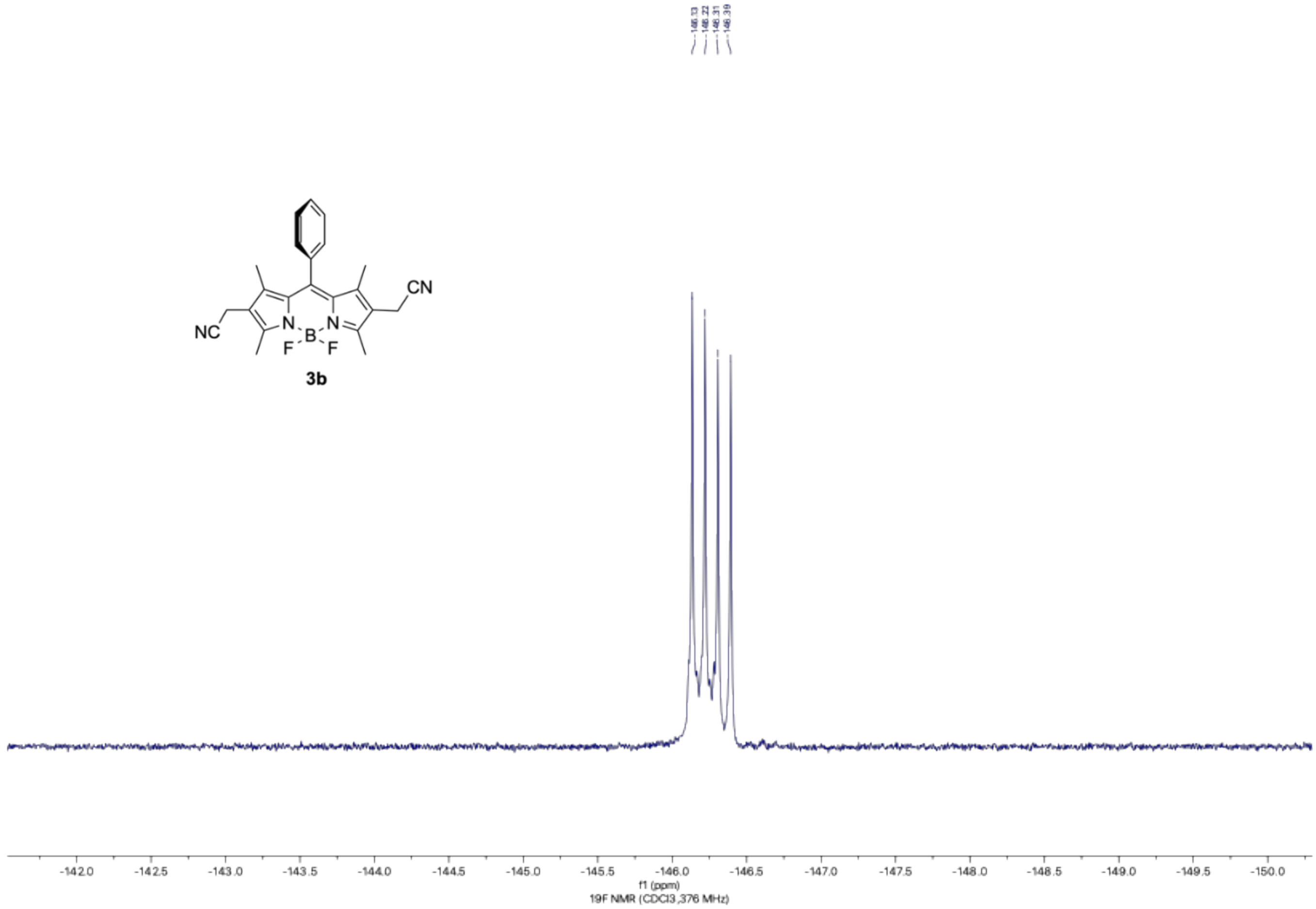

^{19}F-NMR ($CDCl_3$, 376 MHz) of Compound **3b**

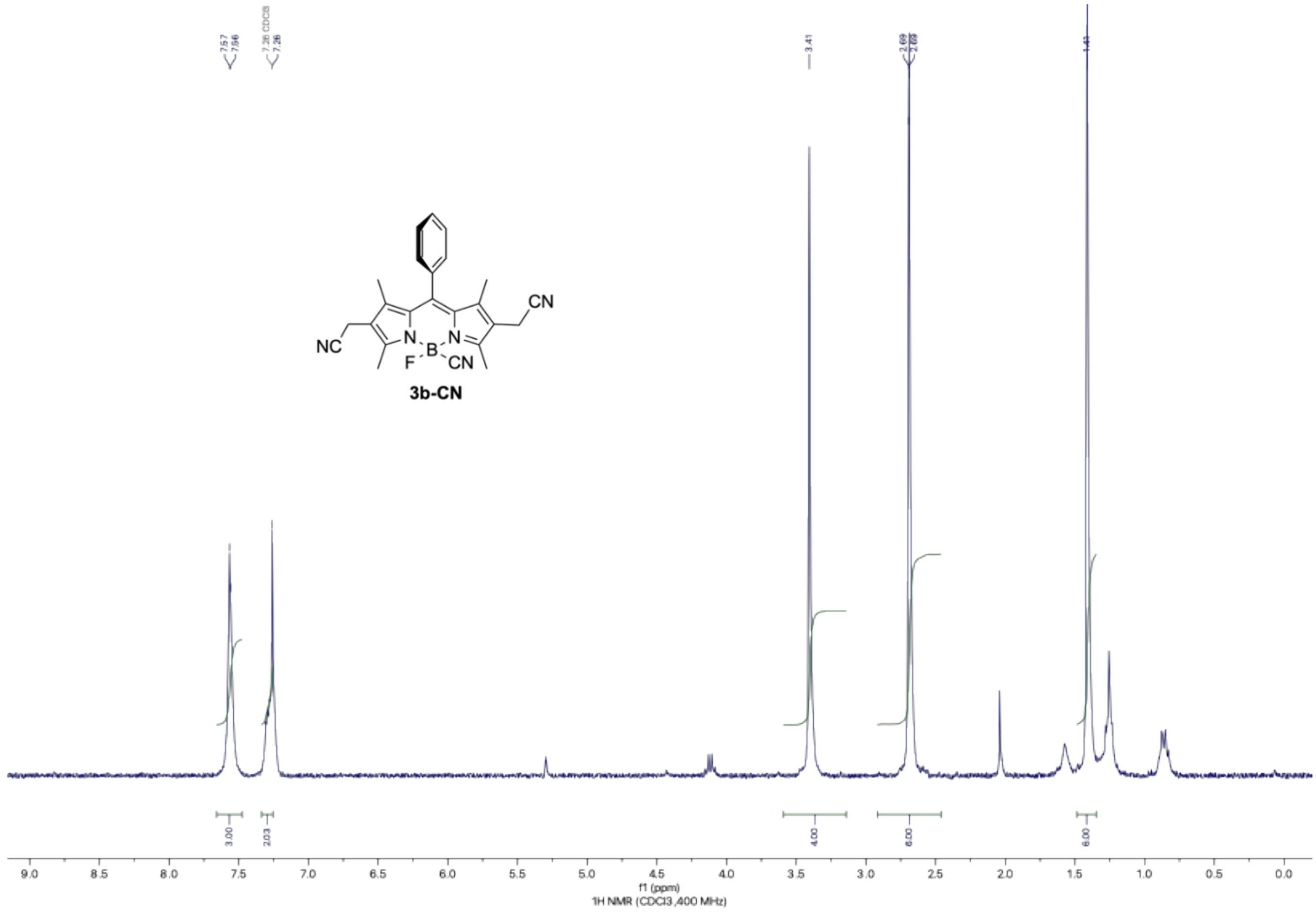

^{1}H-NMR ($CDCl_3$, 400 MHz) of Compound **3b-CN**

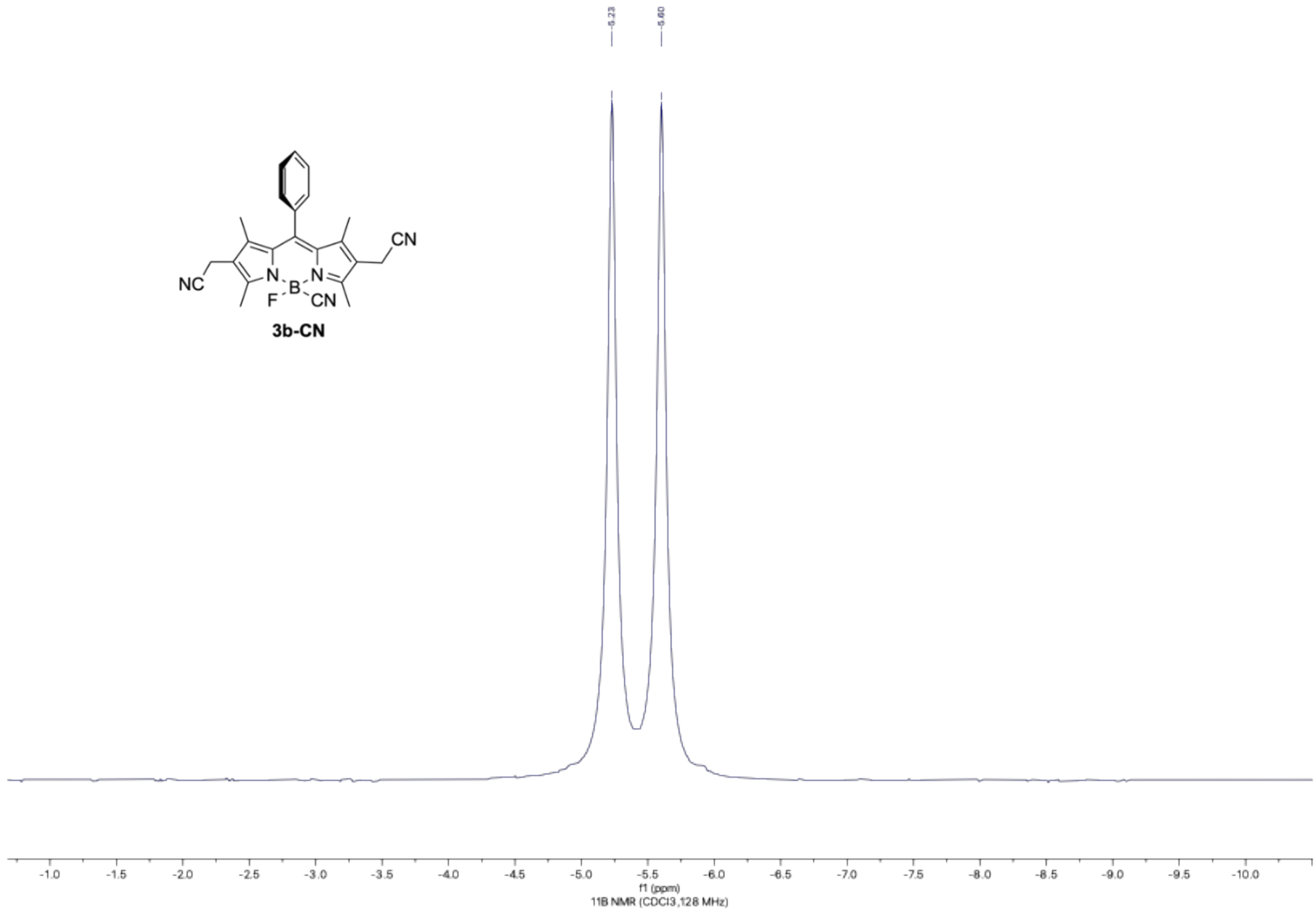

^{11}B-NMR ($CDCl_3$, 128 MHz) of Compound **3b-CN**

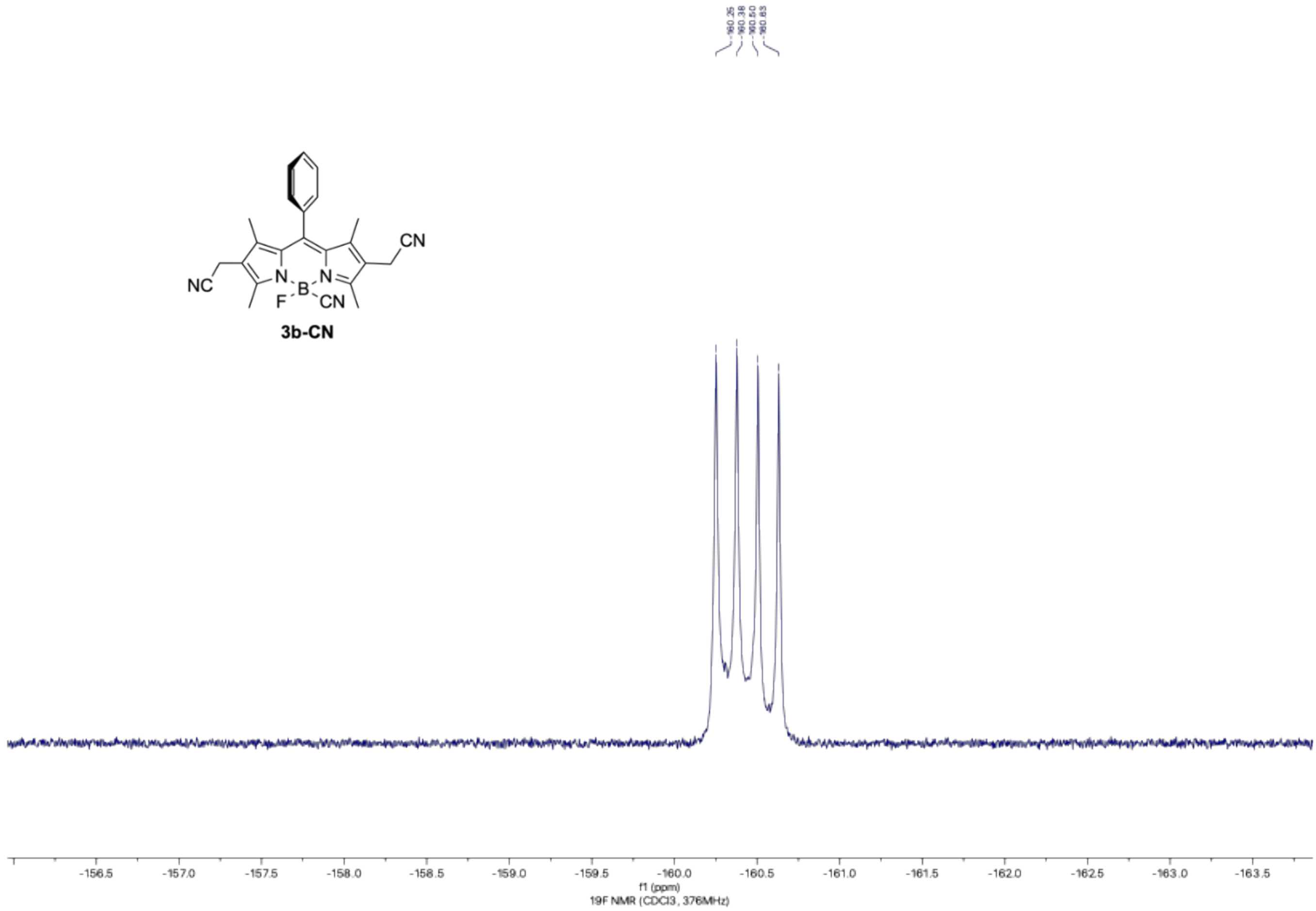

^{19}F-NMR ($CDCl_3$, 376 MHz) of Compound **3b-CN**

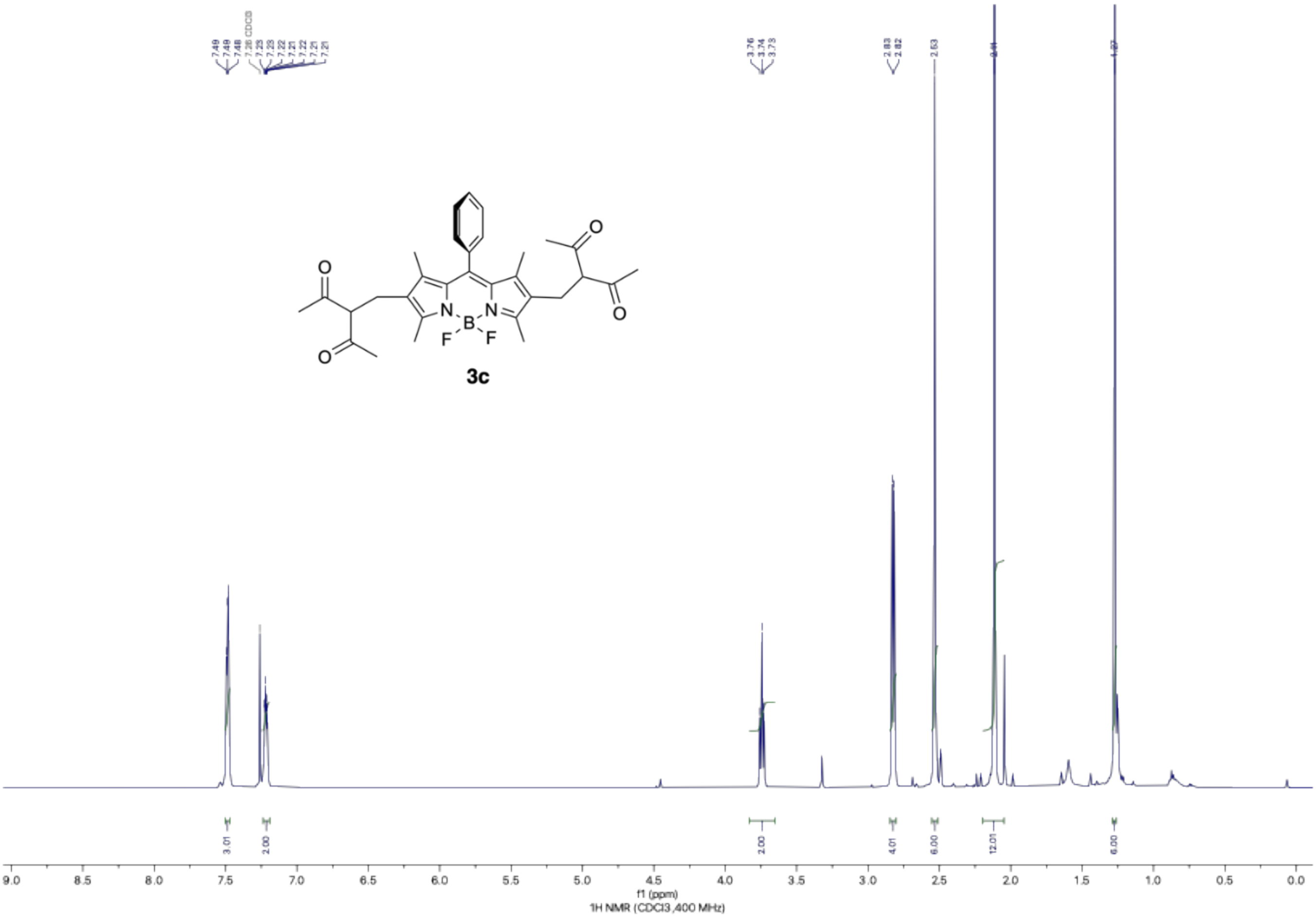

^{1}H-NMR ($CDCl_3$, 400 MHz) of Compound **3c**

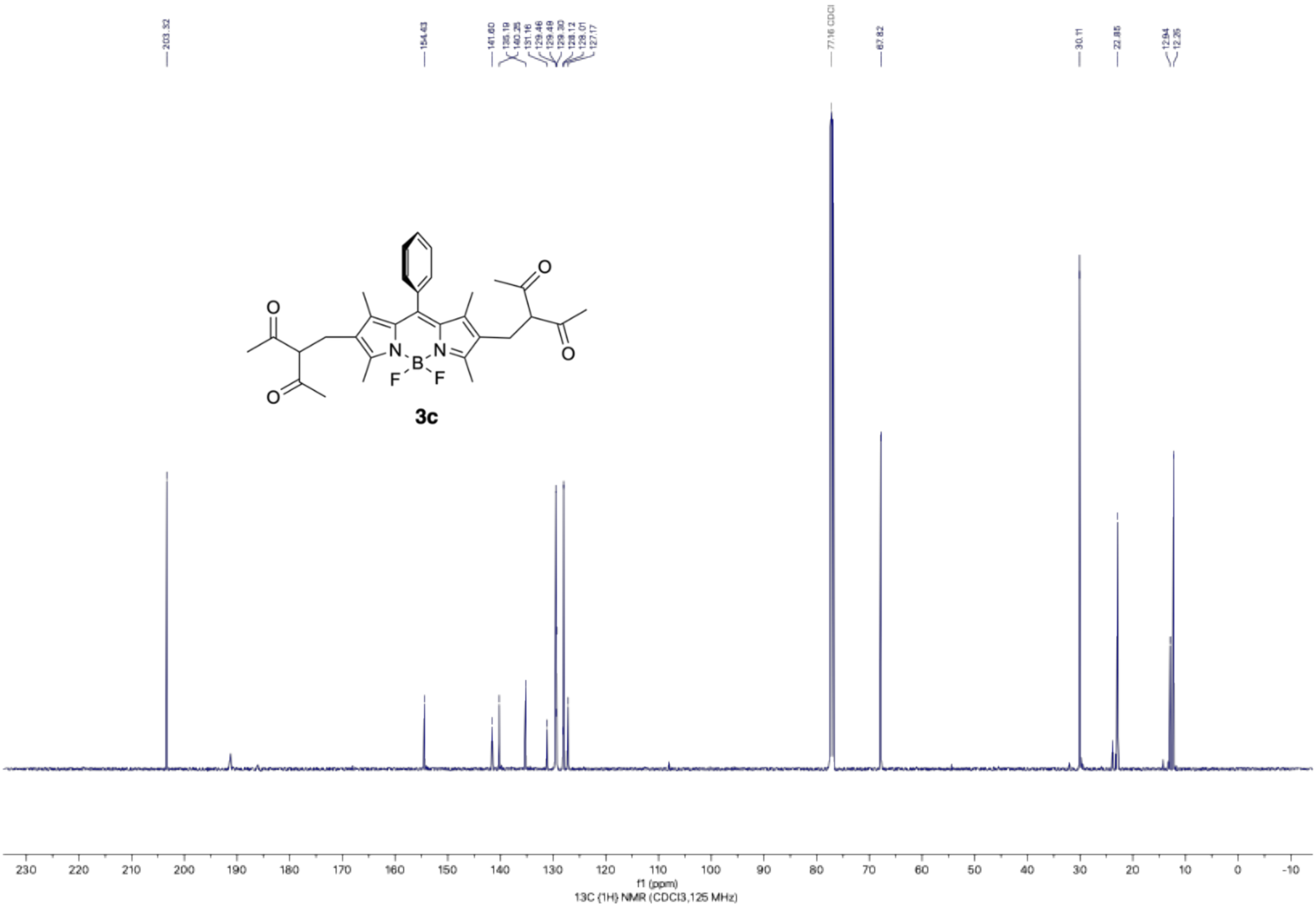

^{13}C {^{1}H}-NMR ($CDCl_3$, 125 MHz) of Compound **3c**

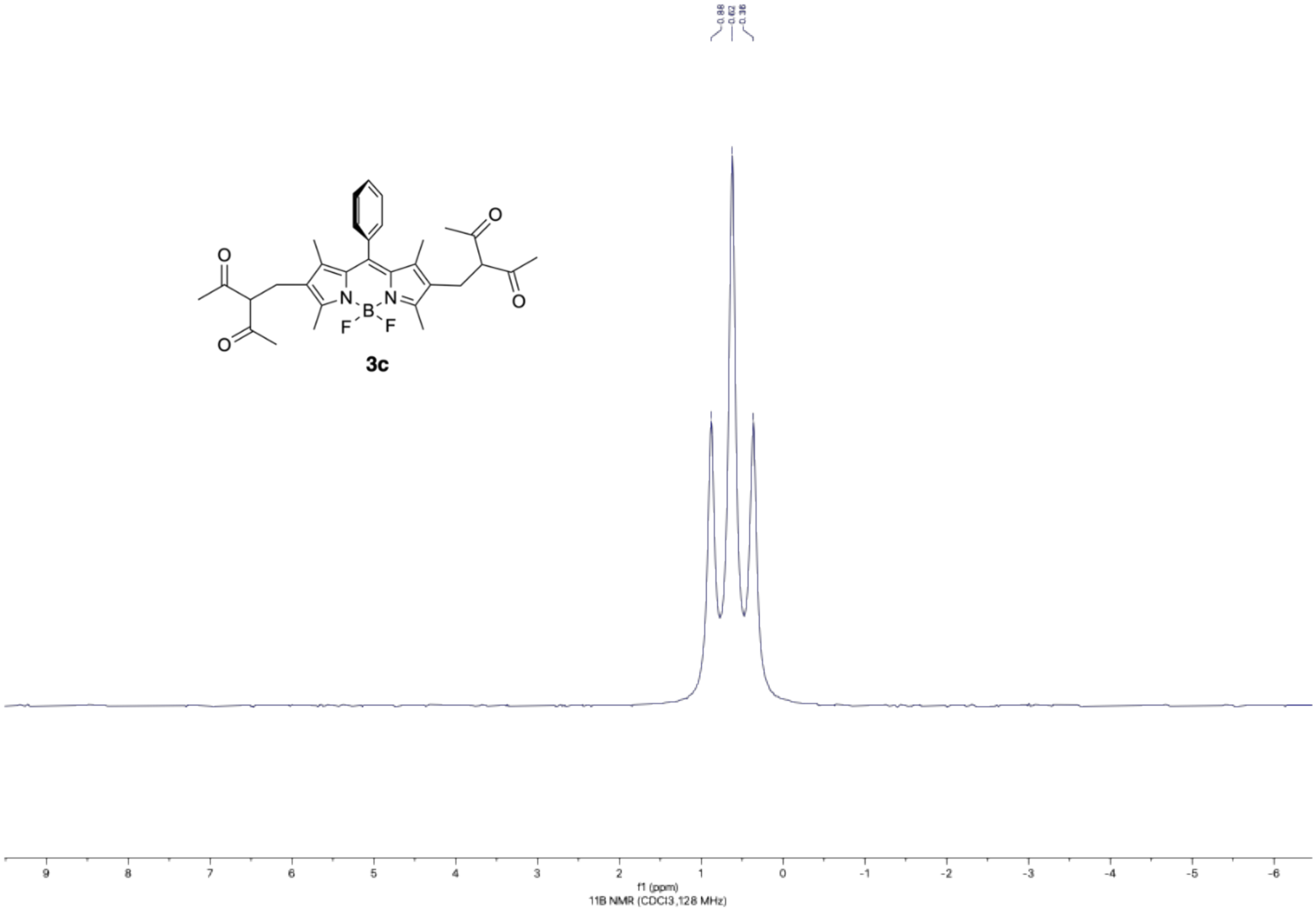

^{11}B-NMR ($CDCl_3$, 128 MHz) of Compound **3c**

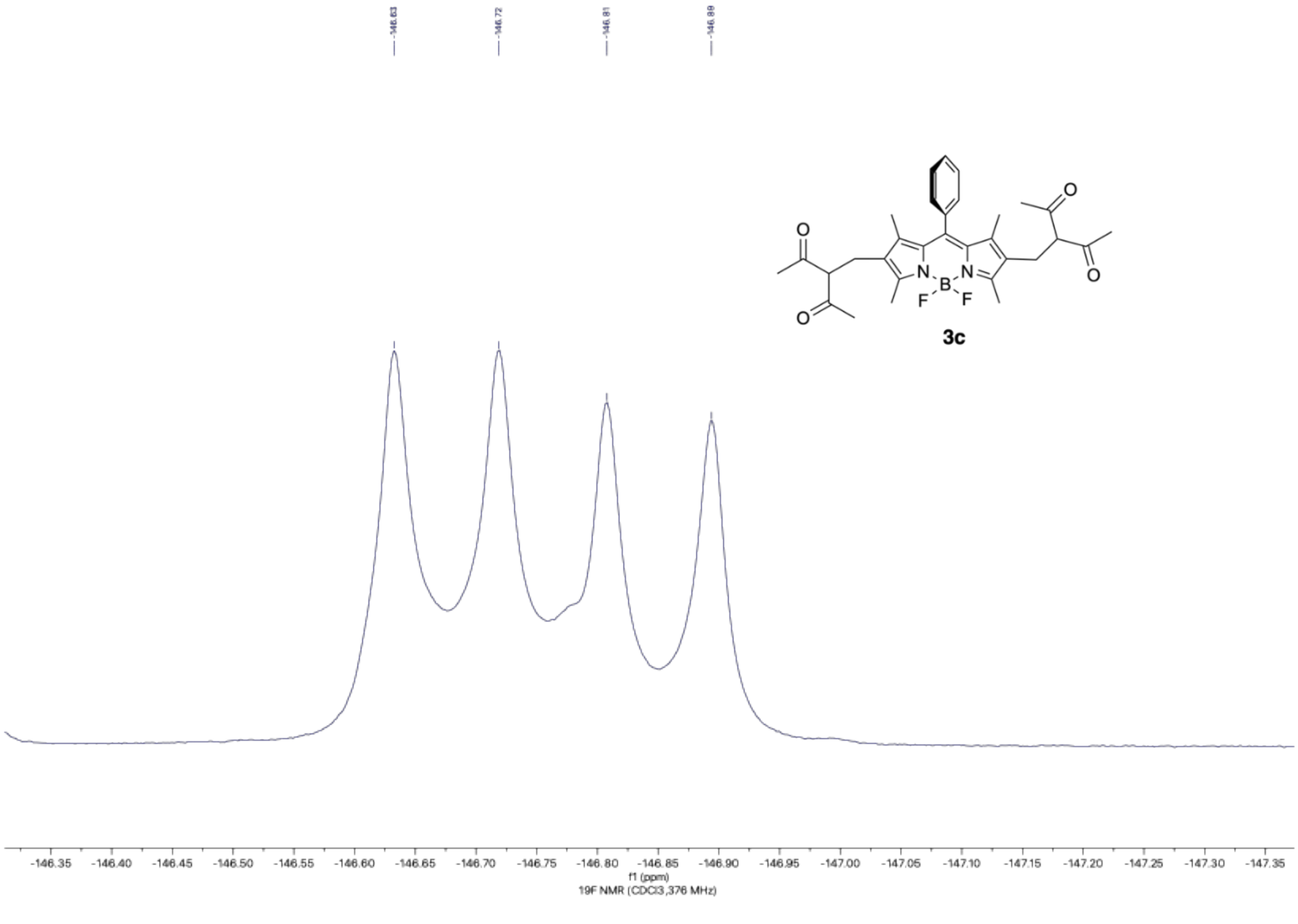

^{19}F-NMR ($CDCl_3$, 376 MHz) of Compound **3c**

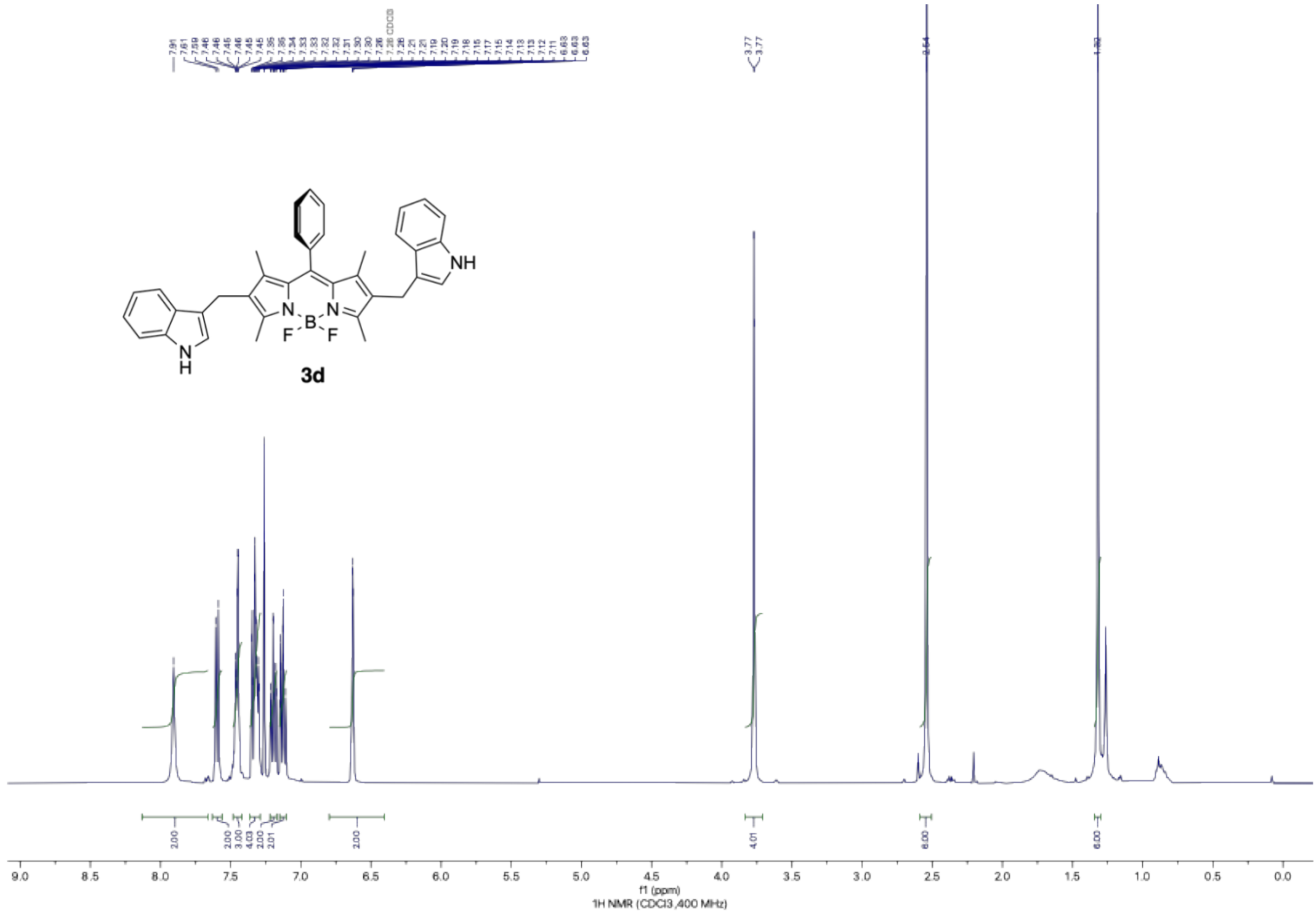

^{1}H-NMR ($CDCl_3$, 400 MHz) of Compound **3d**

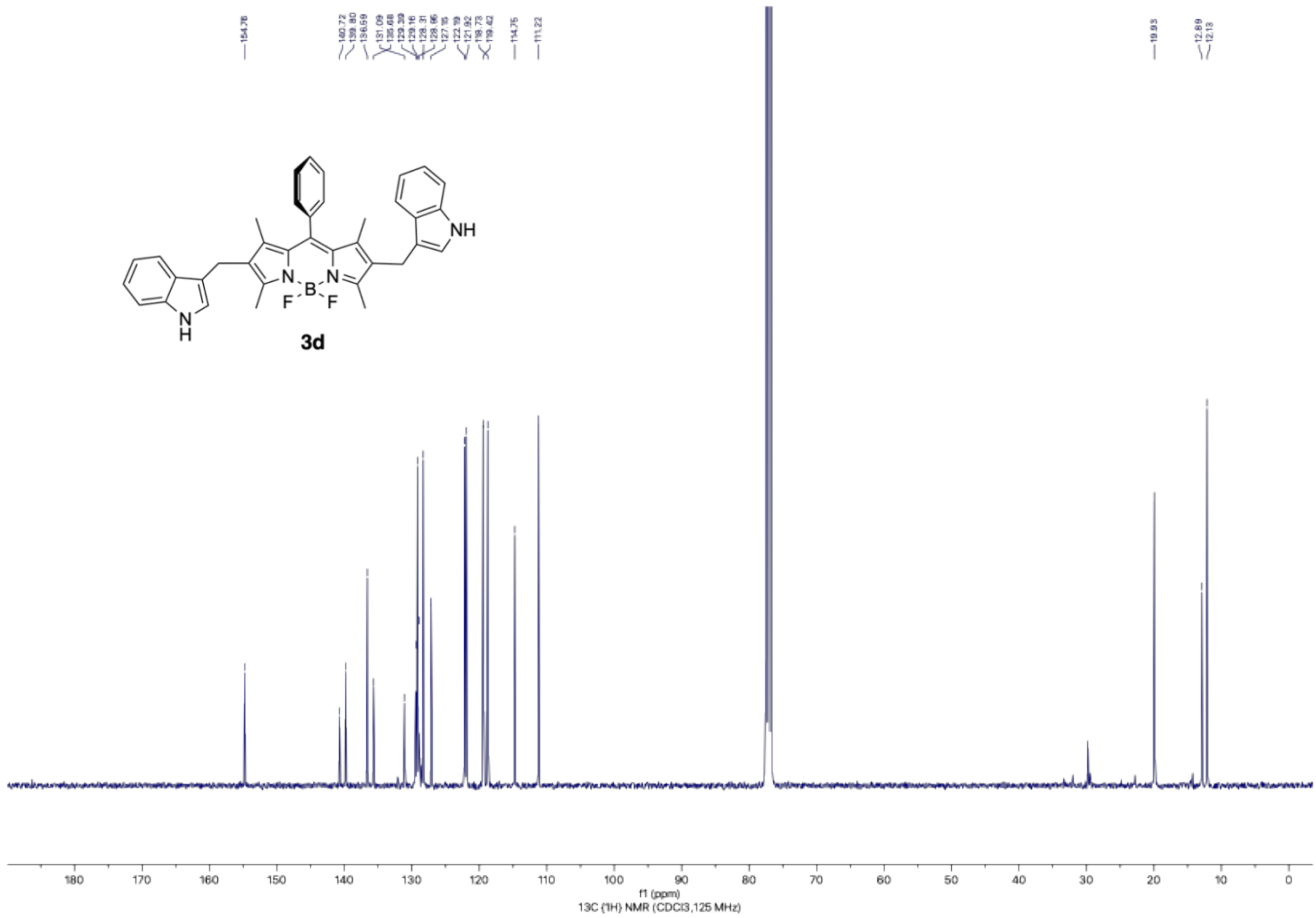

^{13}C {^{1}H}-NMR ($CDCl_3$, 125 MHz) of Compound **3d**

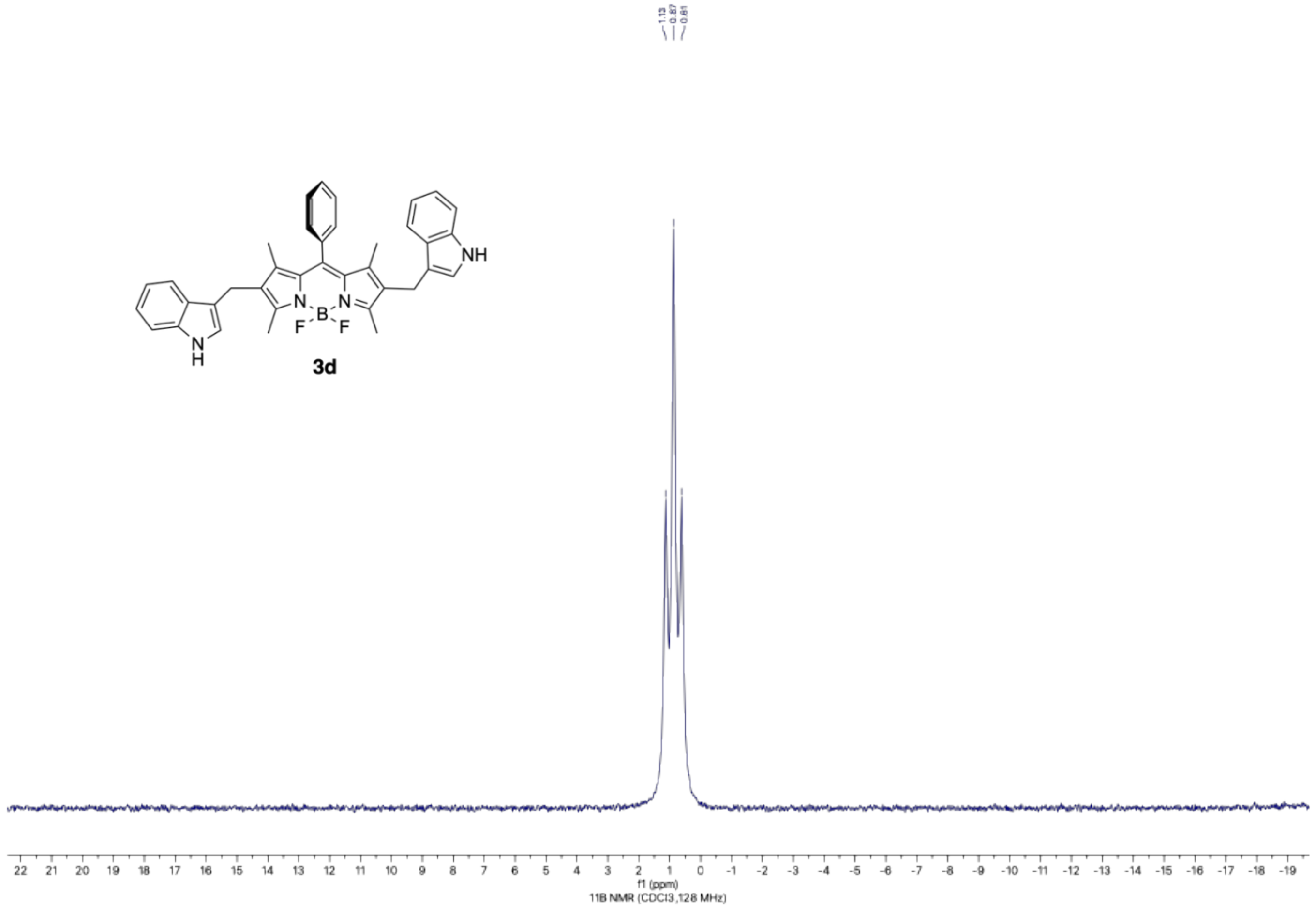

^{11}B-NMR ($CDCl_3$, 128 MHz) of Compound **3d**

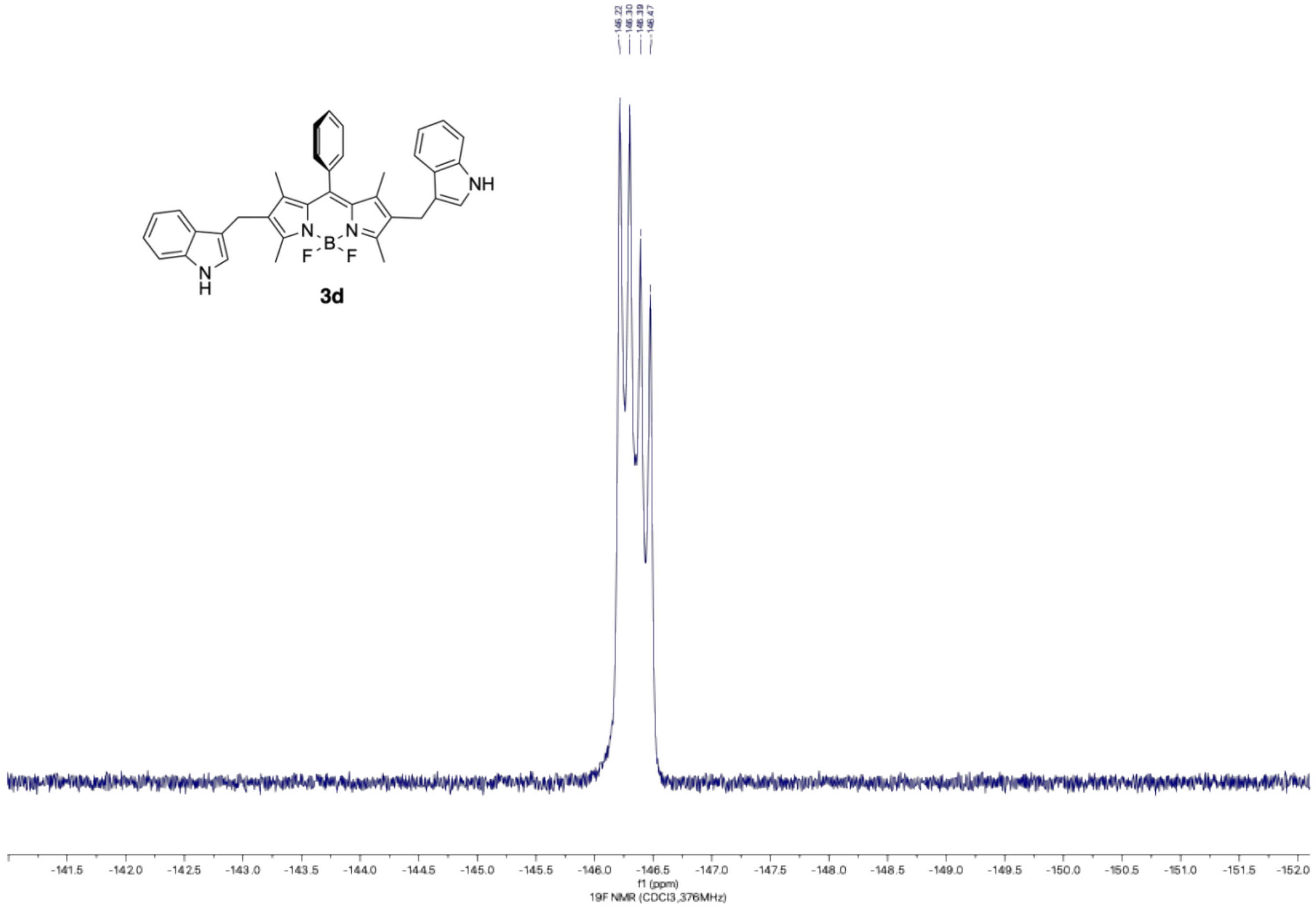

^{19}F-NMR ($CDCl_3$, 376 MHz) of Compound **3d**

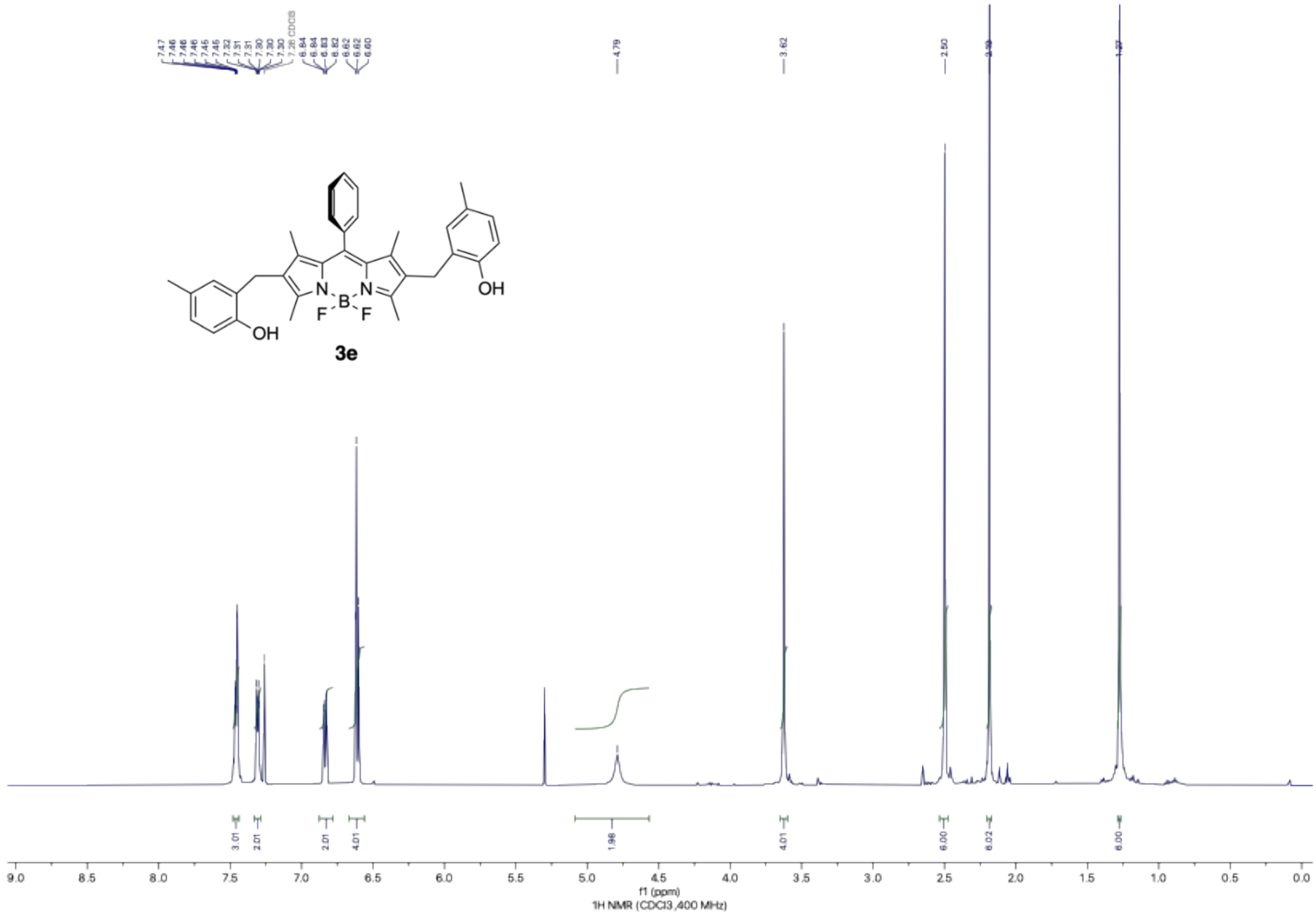

^{1}H-NMR ($CDCl_3$, 400 MHz) of Compound **3e**

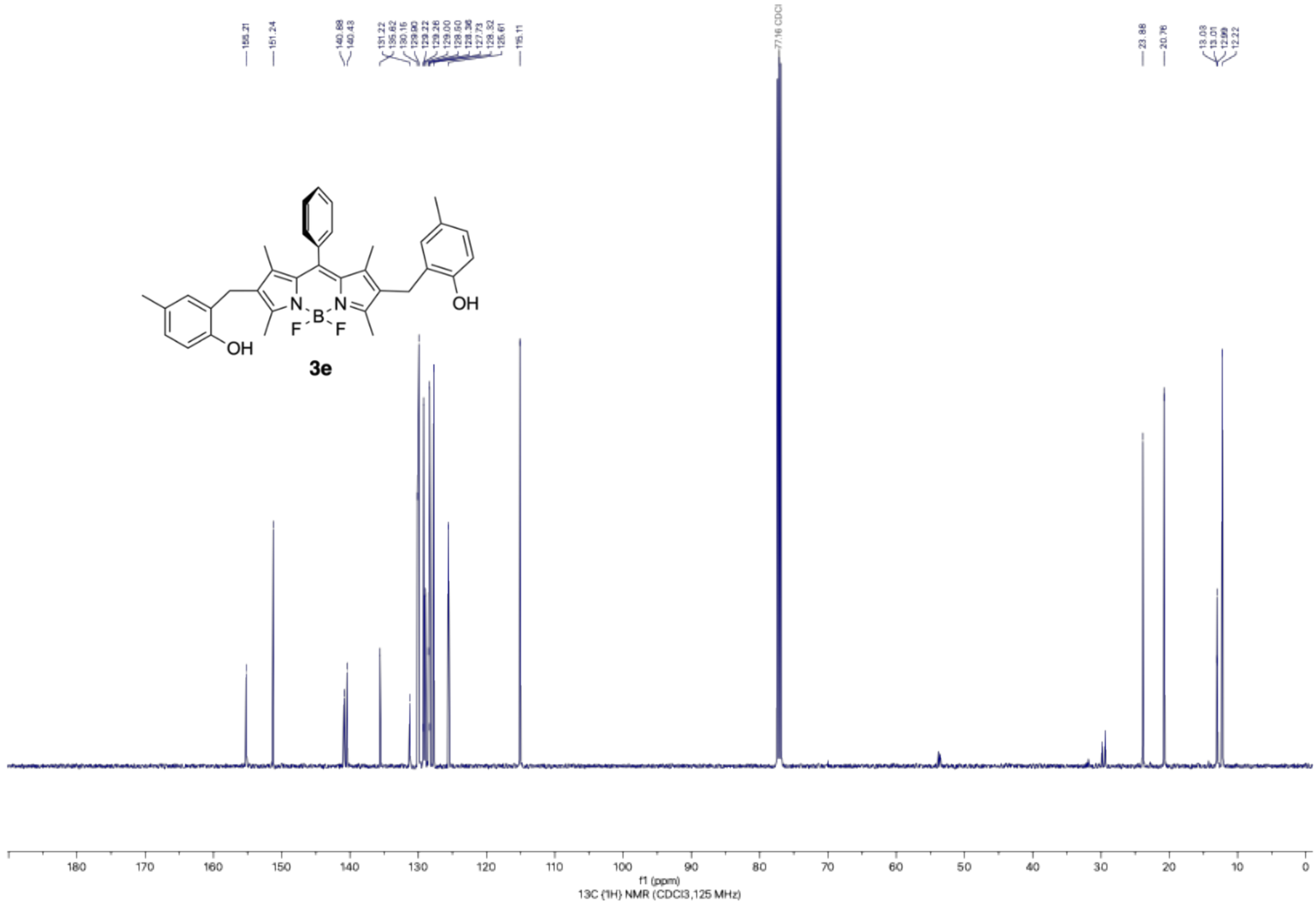

^{13}C {^{1}H}-NMR ($CDCl_3$, 125 MHz) of Compound **3e**

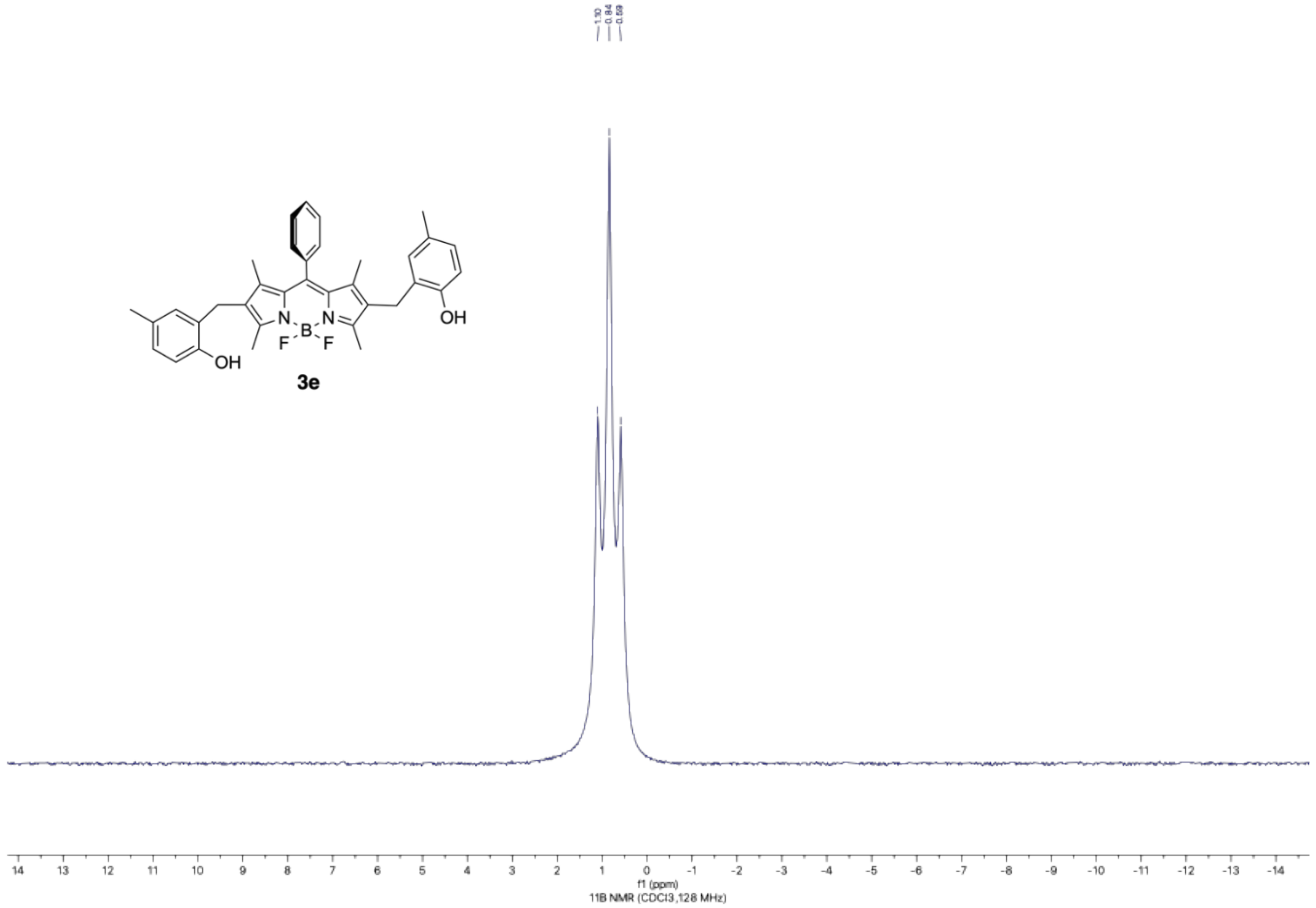

^{11}B-NMR ($CDCl_3$, 128 MHz) of Compound **3e**

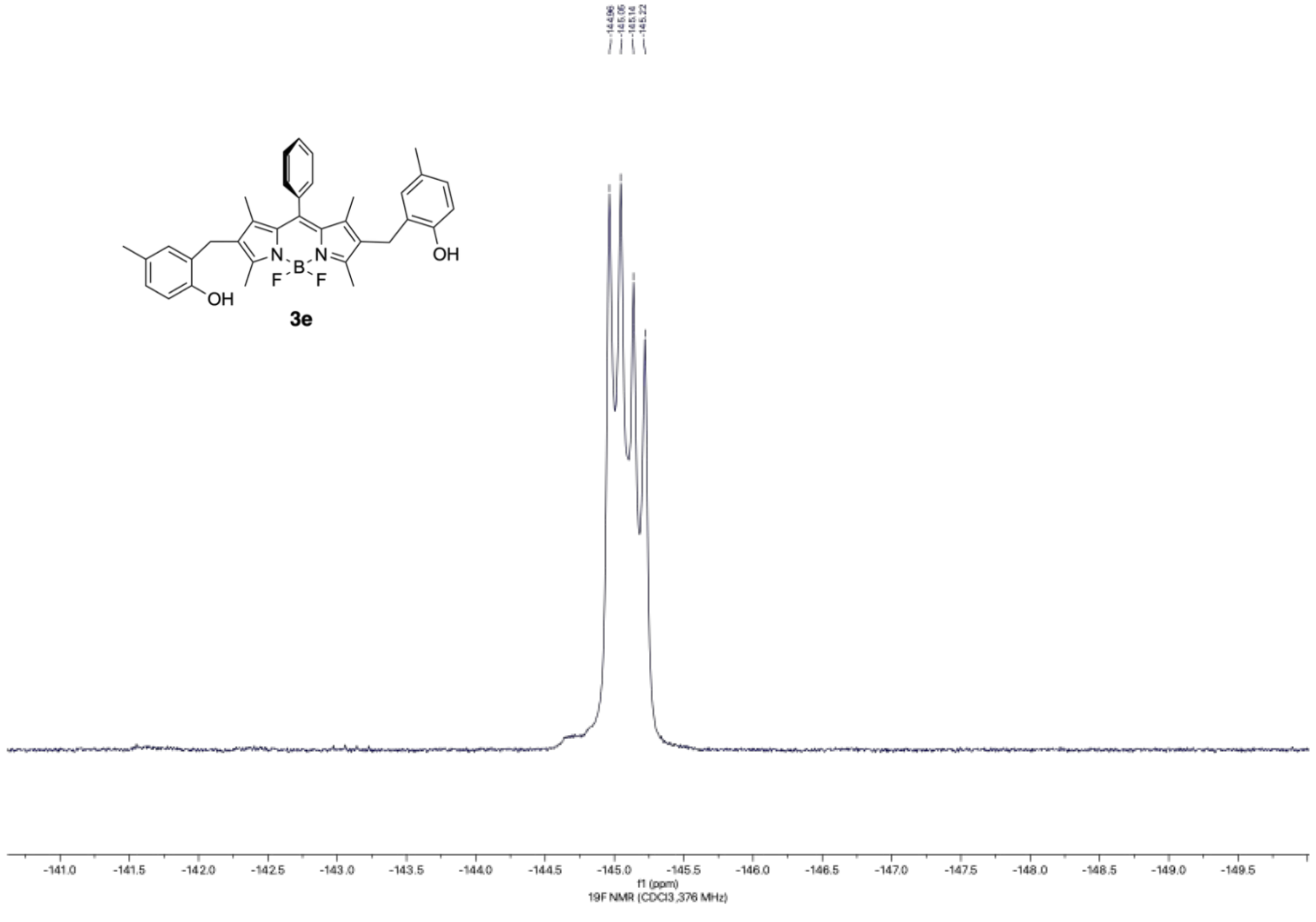

^{19}F-NMR ($CDCl_3$, 376 MHz) of Compound **3e**

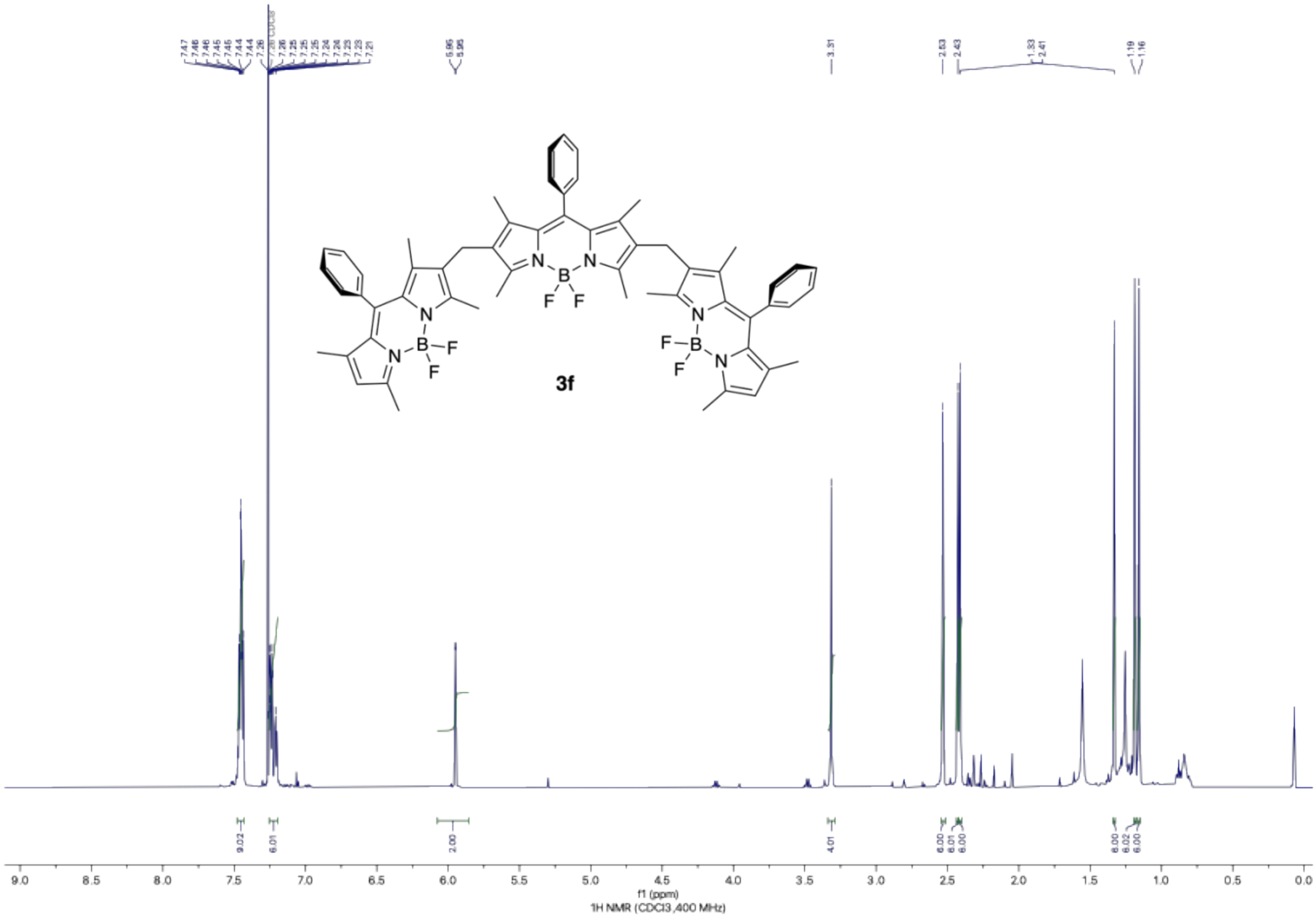

^{1}H-NMR ($CDCl_3$, 400 MHz) of Compound **3f**

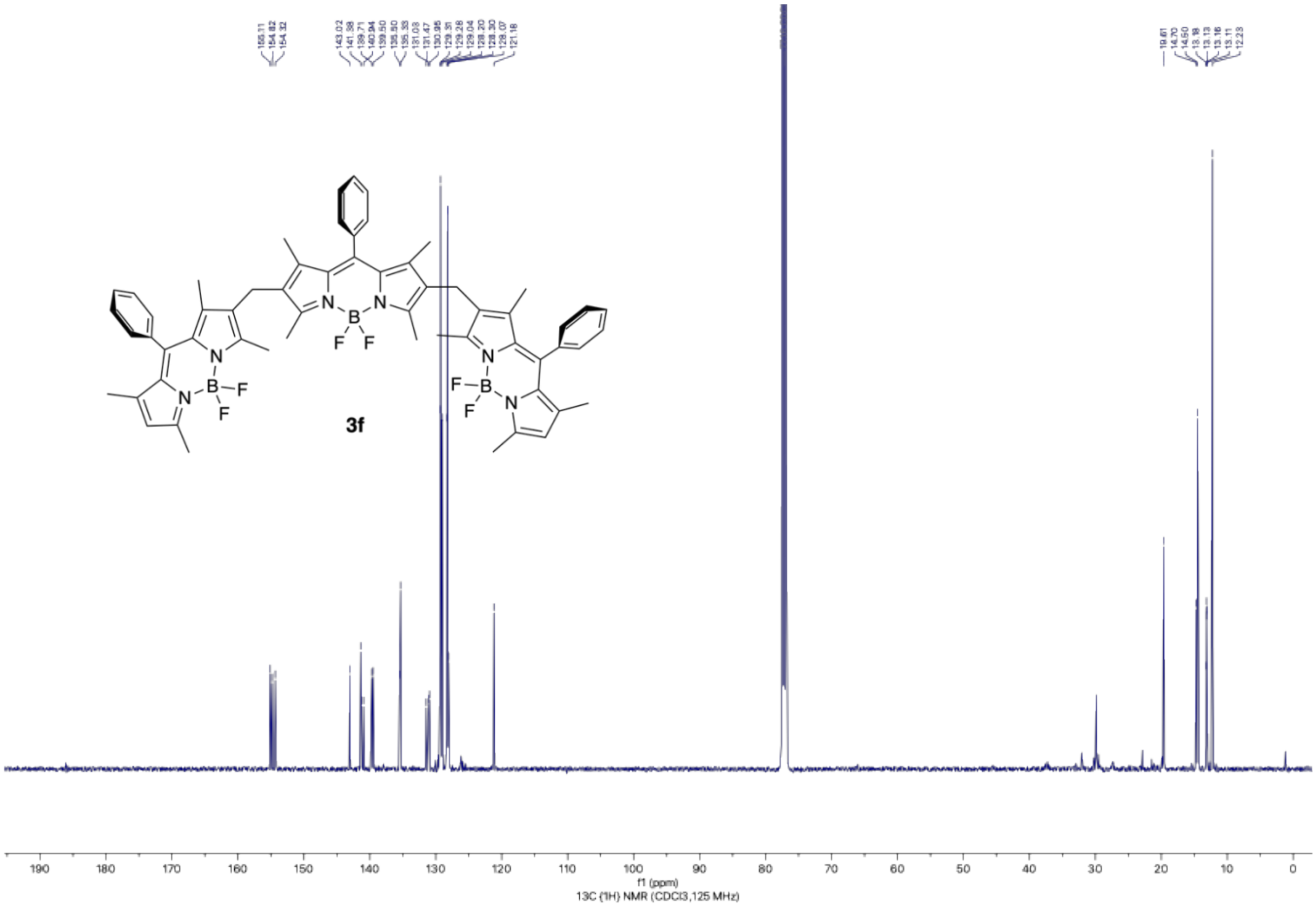

^{13}C {^{1}H}-NMR ($CDCl_3$, 125 MHz) of Compound **3f**

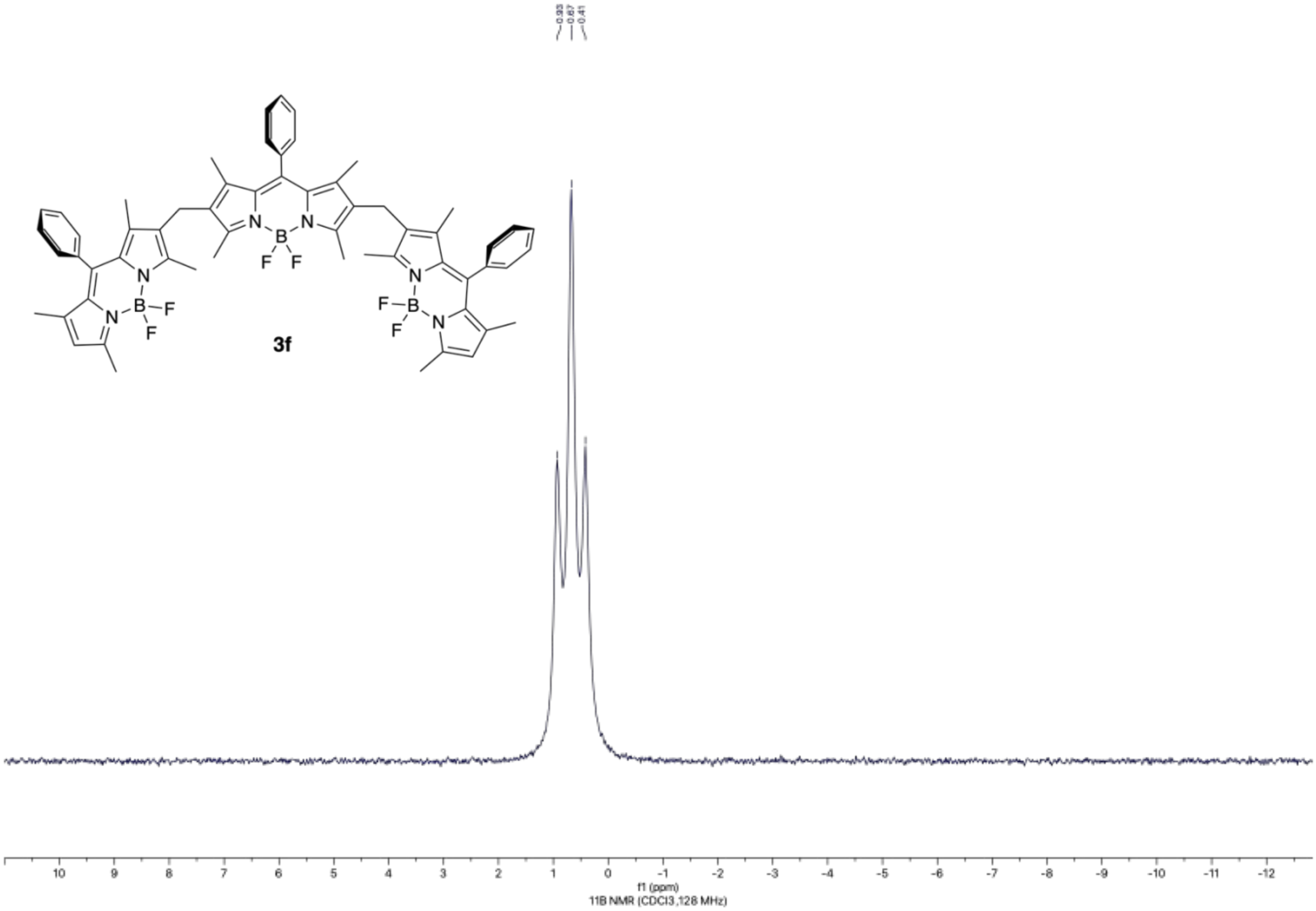

^{11}B-NMR ($CDCl_3$, 128 MHz) of Compound **3f**

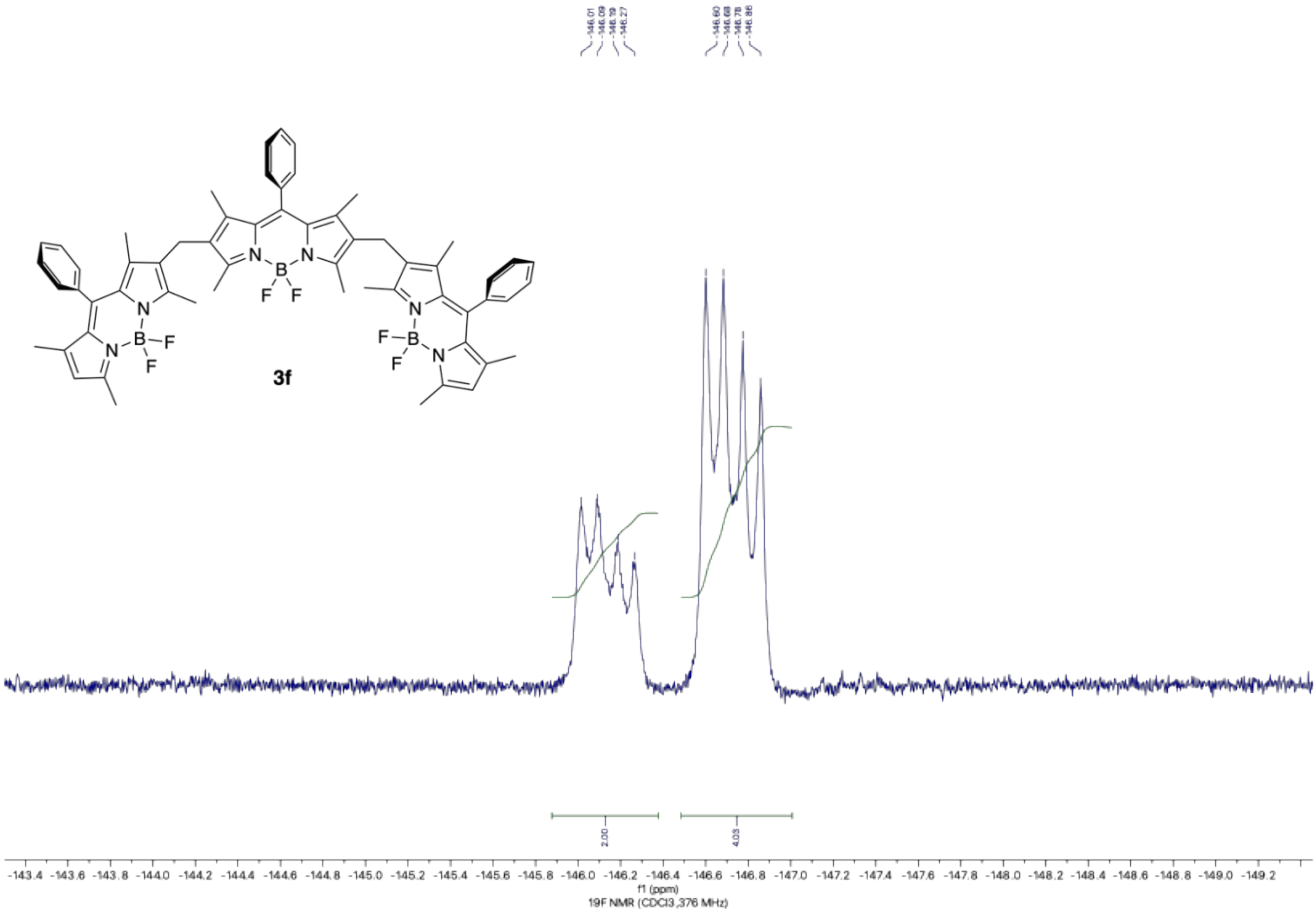

^{19}F-NMR ($CDCl_3$, 376 MHz) of Compound **3f**

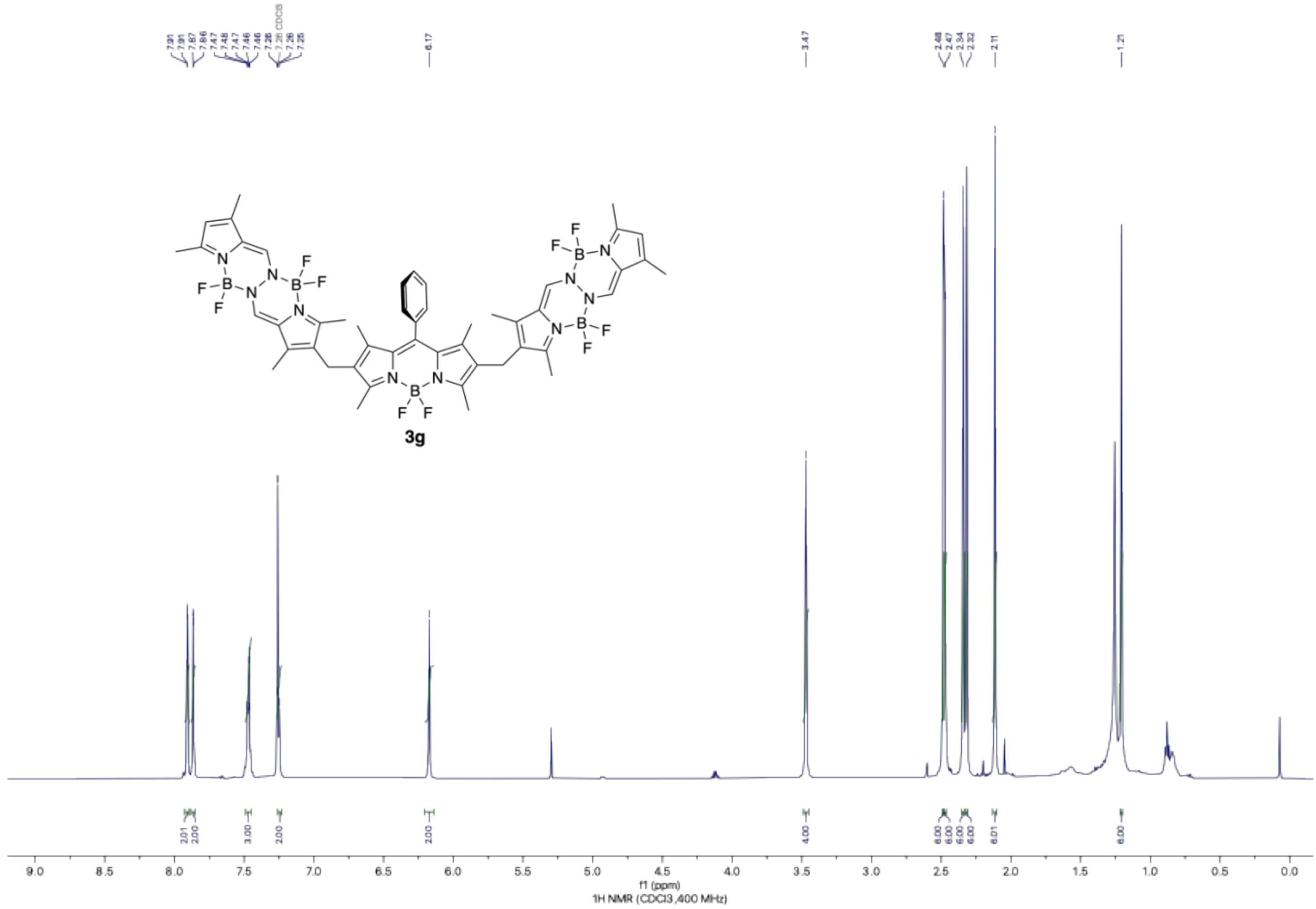

^{1}H-NMR ($CDCl_3$, 400 MHz) of Compound **3g**

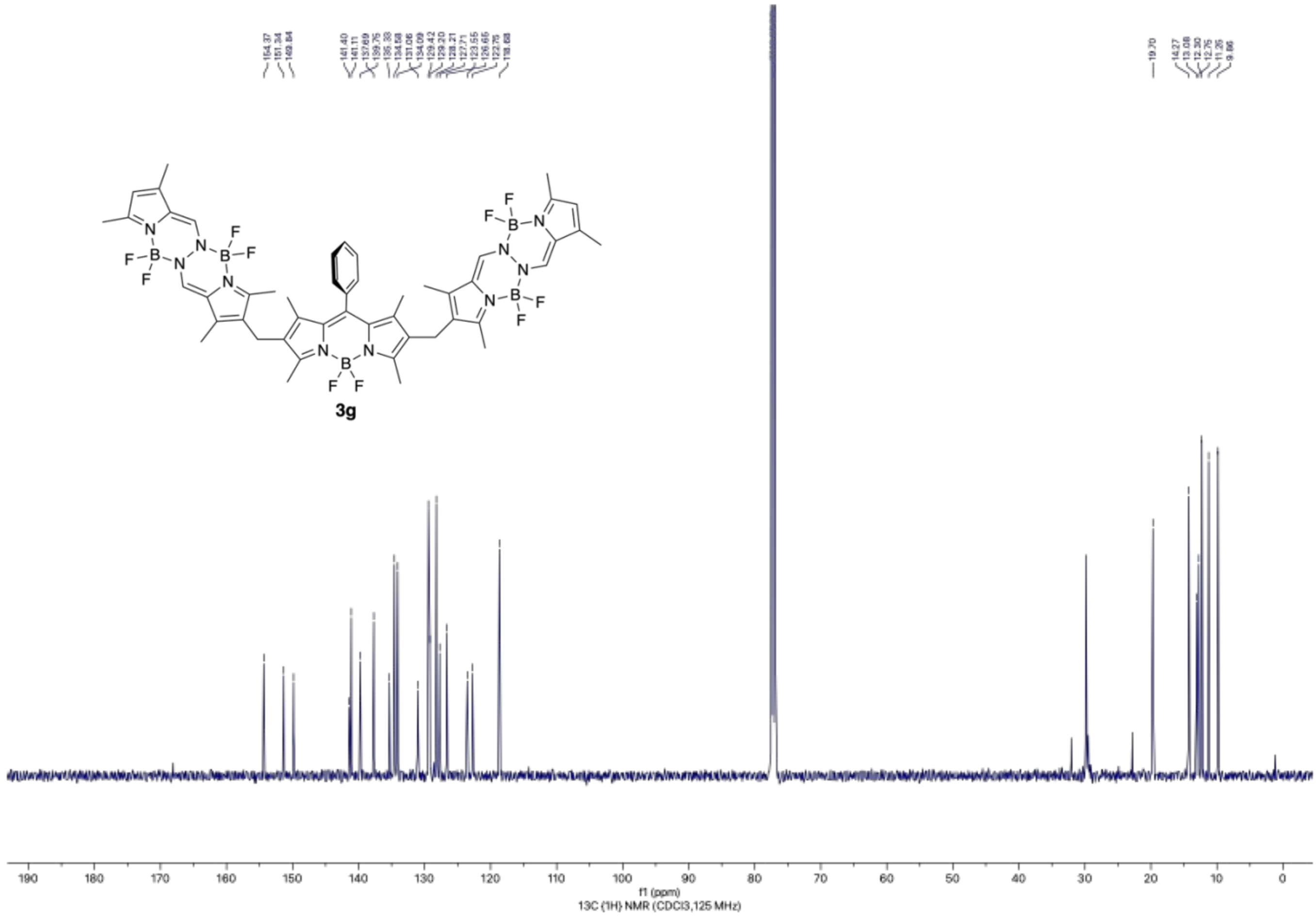

^{13}C {^{1}H}-NMR ($CDCl_3$, 125 MHz) of Compound **3g**

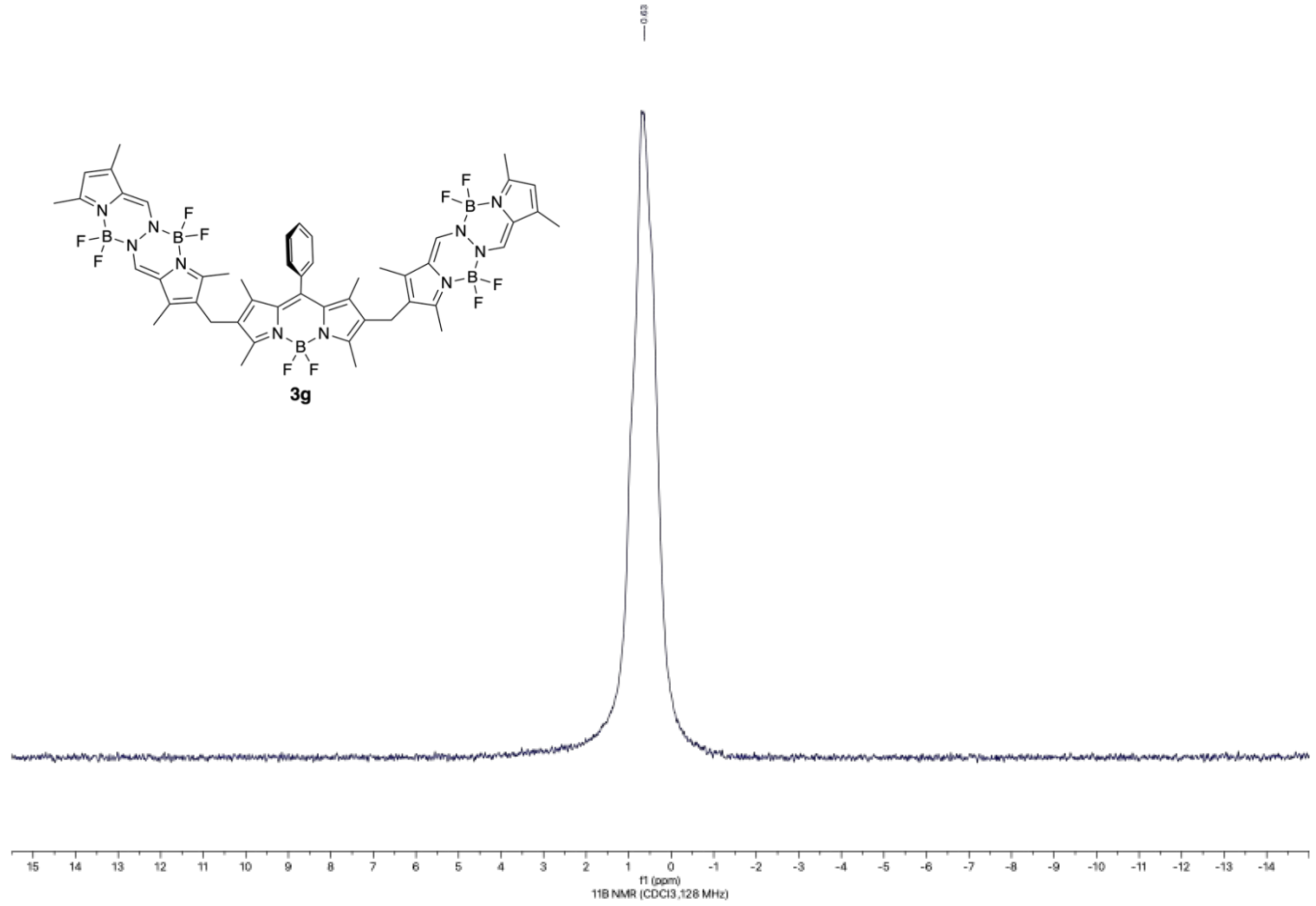

^{11}B-NMR ($CDCl_3$, 128 MHz) of Compound **3g**

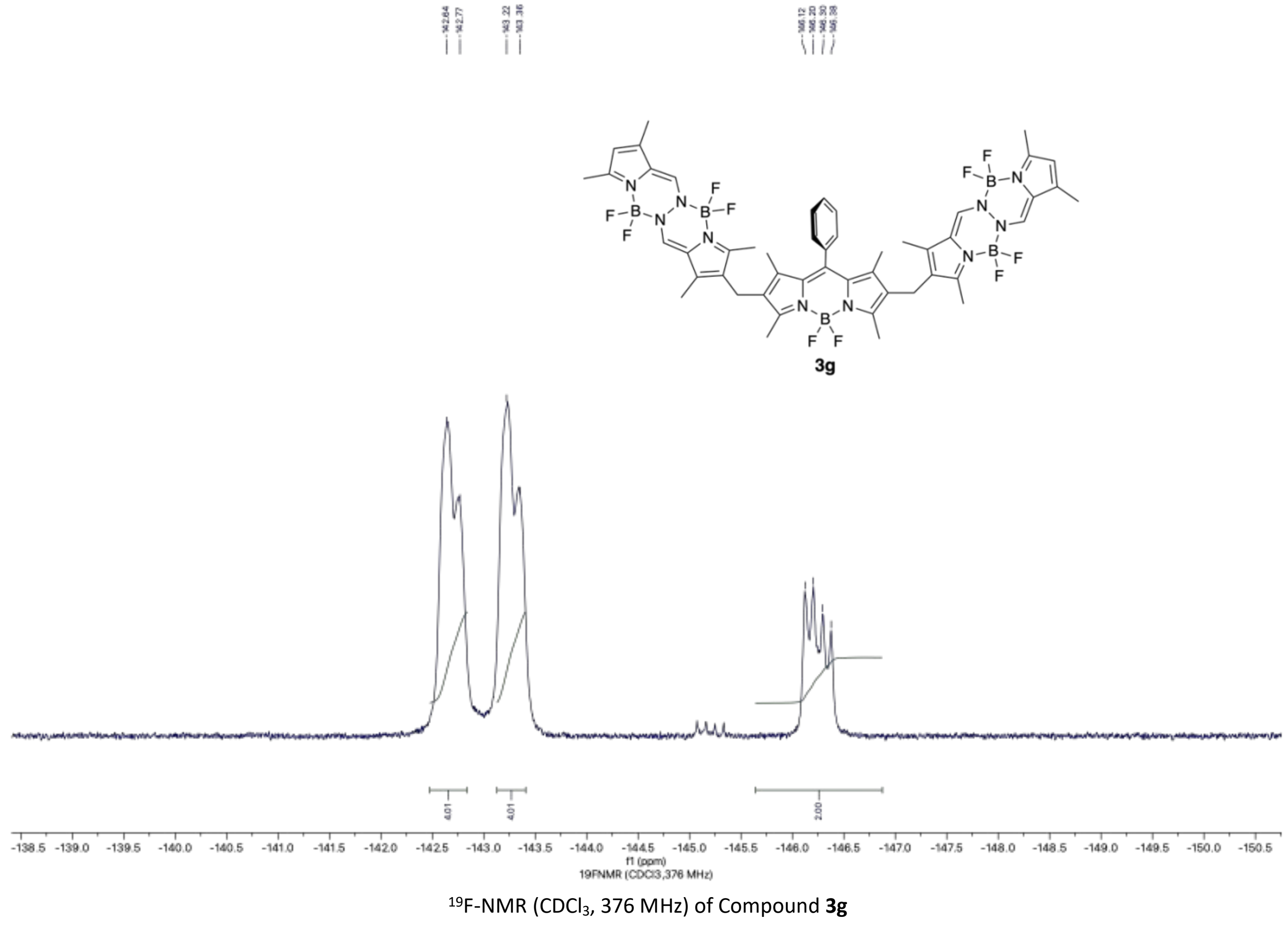

^{19}F-NMR ($CDCl_3$, 376 MHz) of Compound **3g**

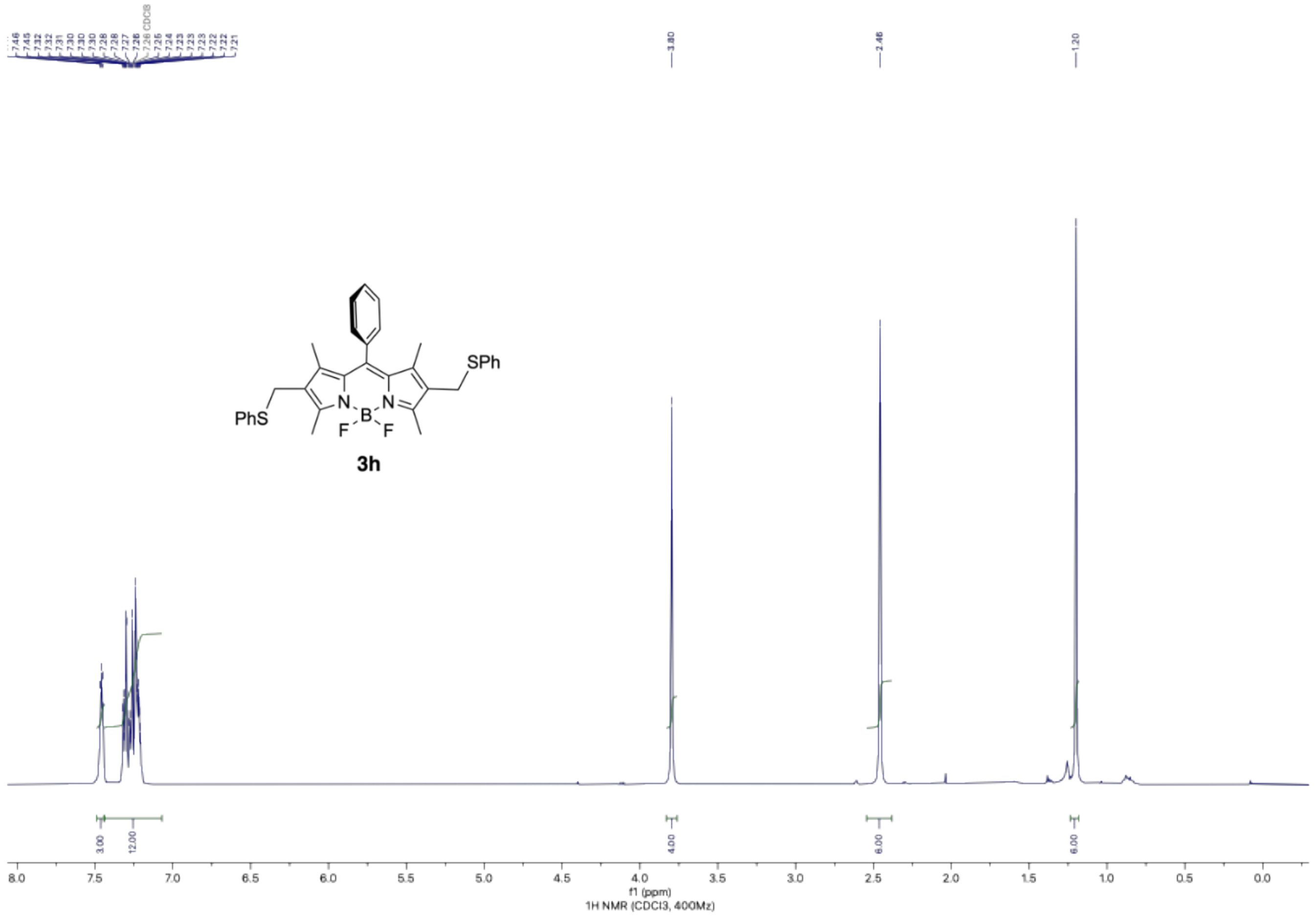

^{1}H-NMR ($CDCl_3$, 400 MHz) of Compound **3h**

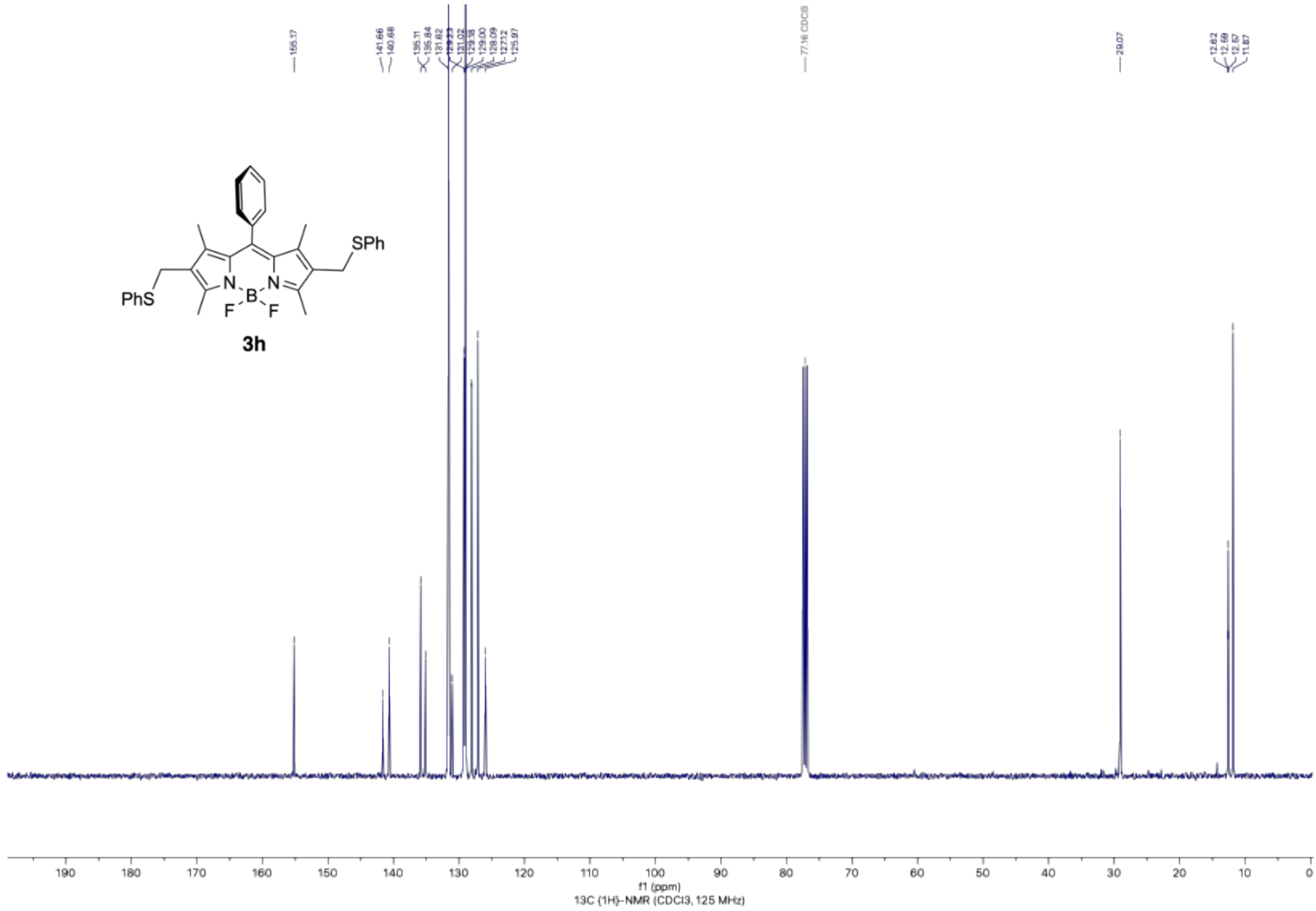

^{13}C {^{1}H}-NMR ($CDCl_3$, 125 MHz) of Compound **3h**

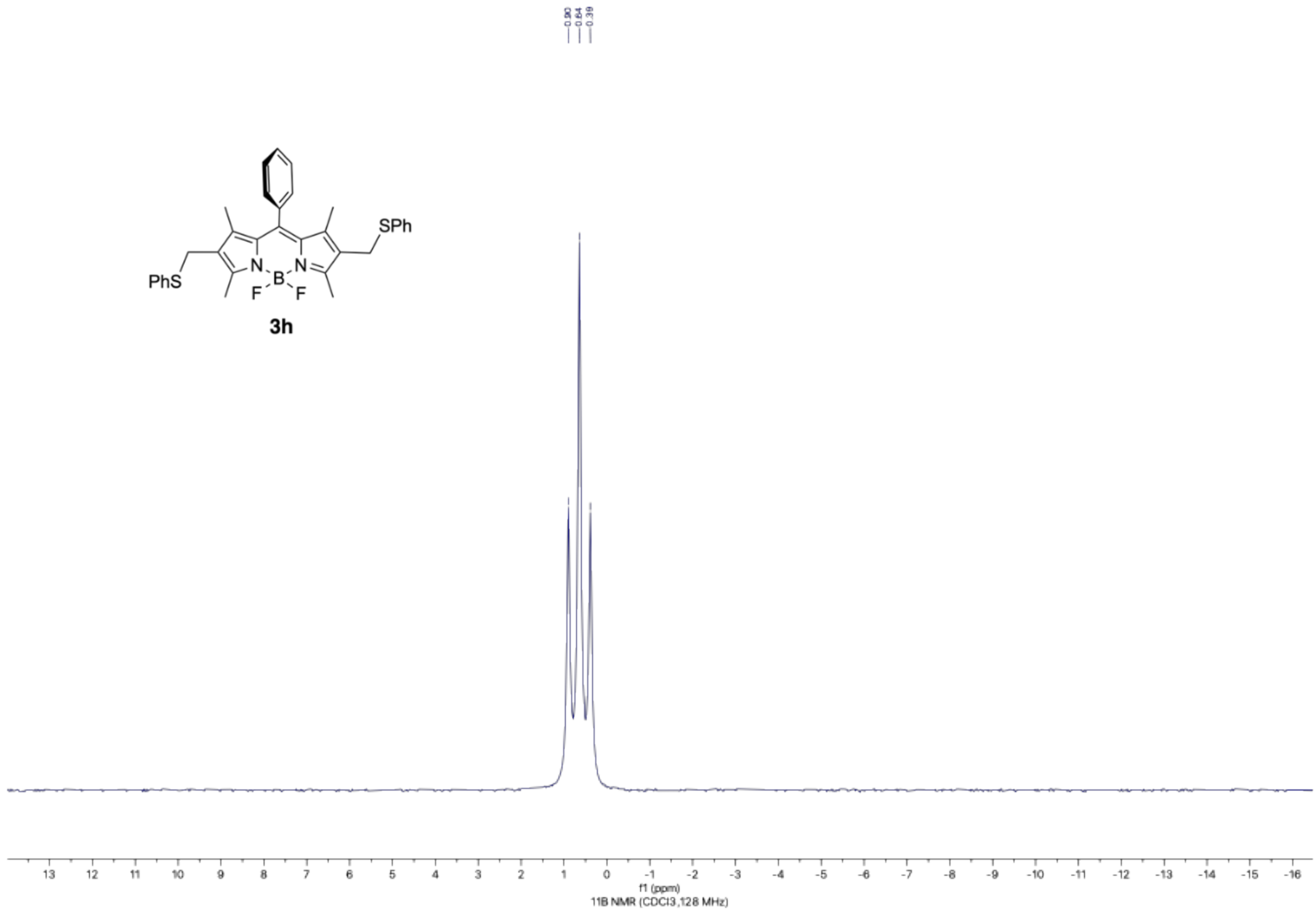

^{11}B-NMR ($CDCl_3$, 128 MHz) of Compound **3h**

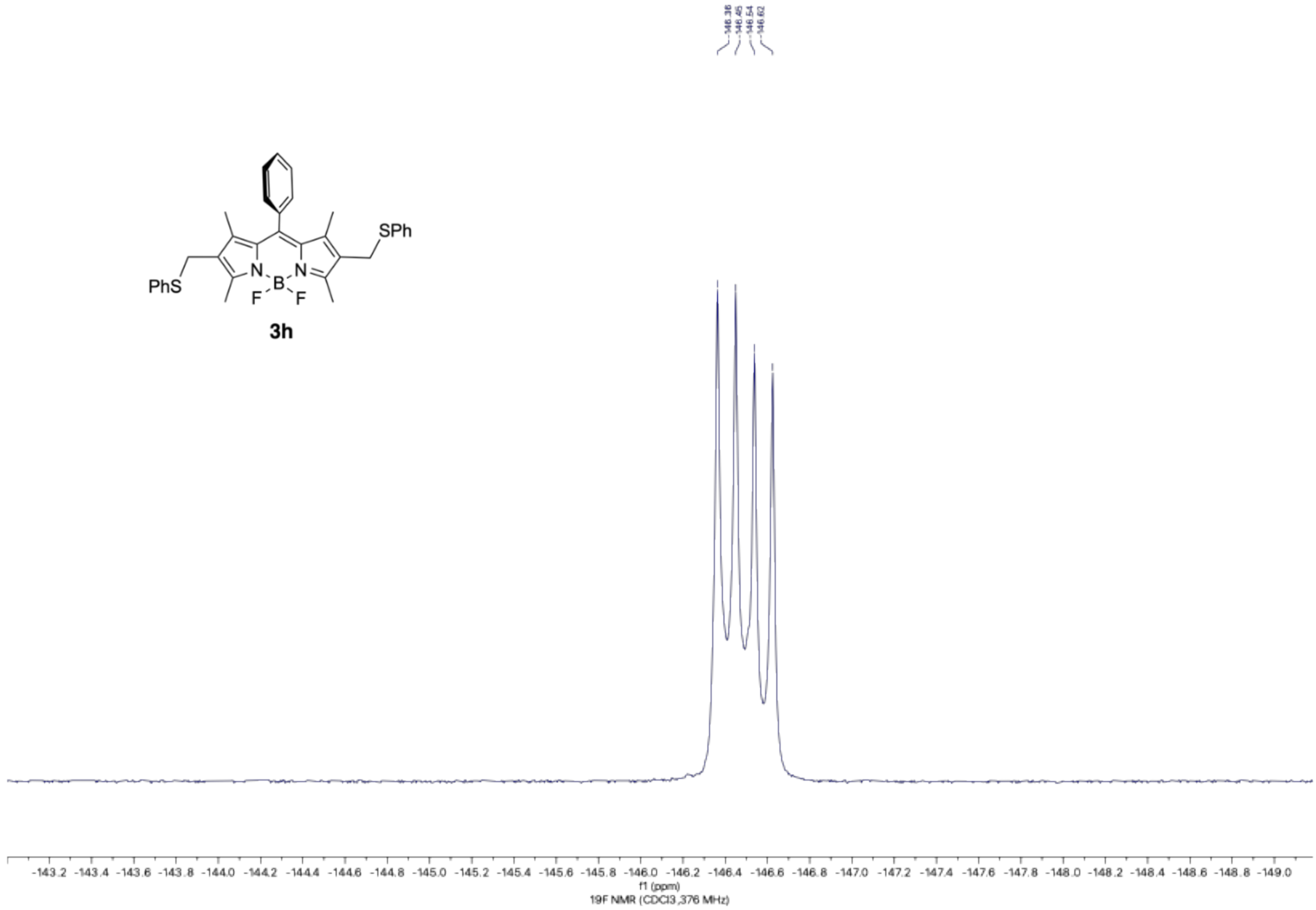

^{19}F-NMR ($CDCl_3$, 376 MHz) of Compound **3h**

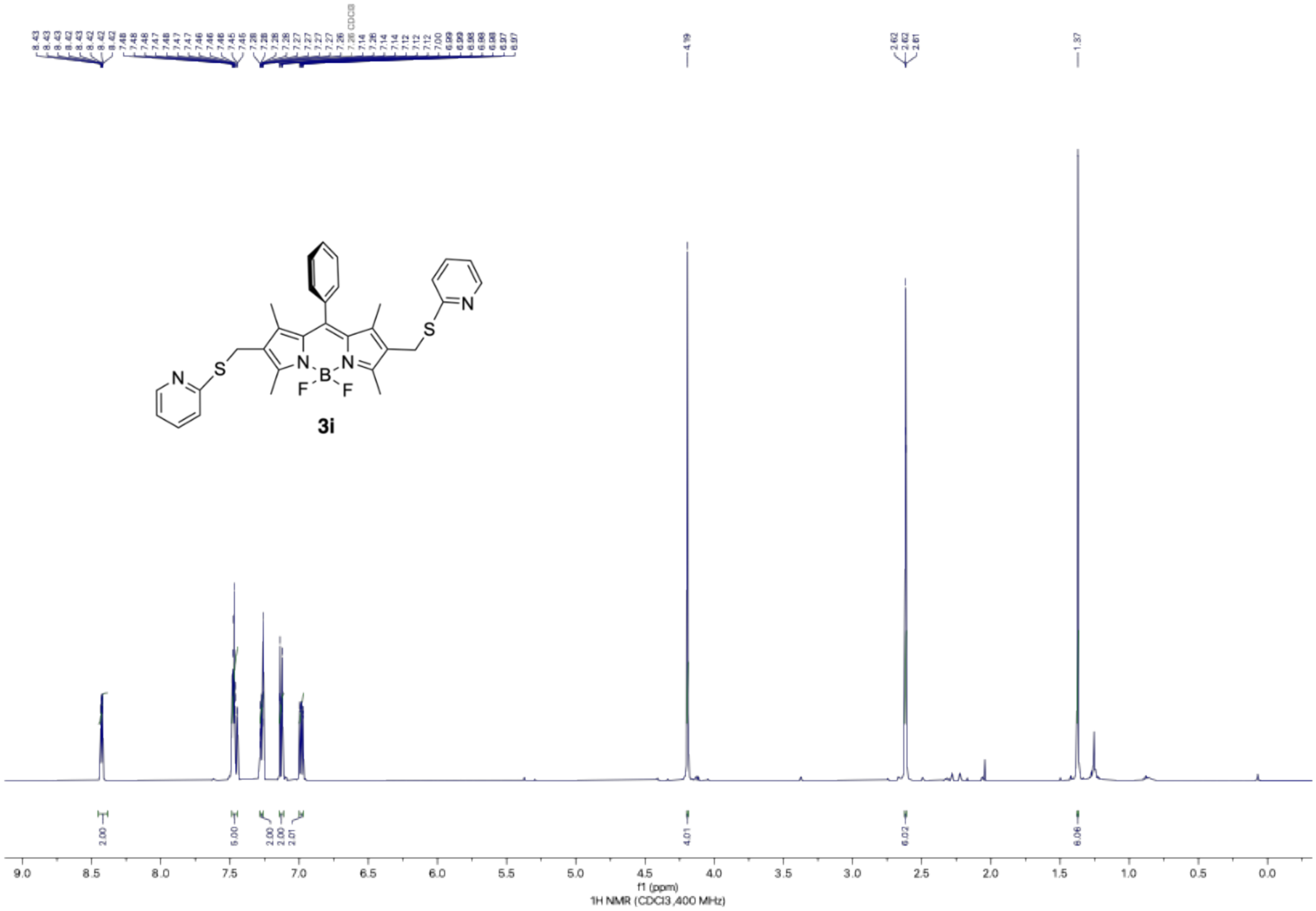

^{1}H-NMR ($CDCl_3$, 400 MHz) of Compound **3i**

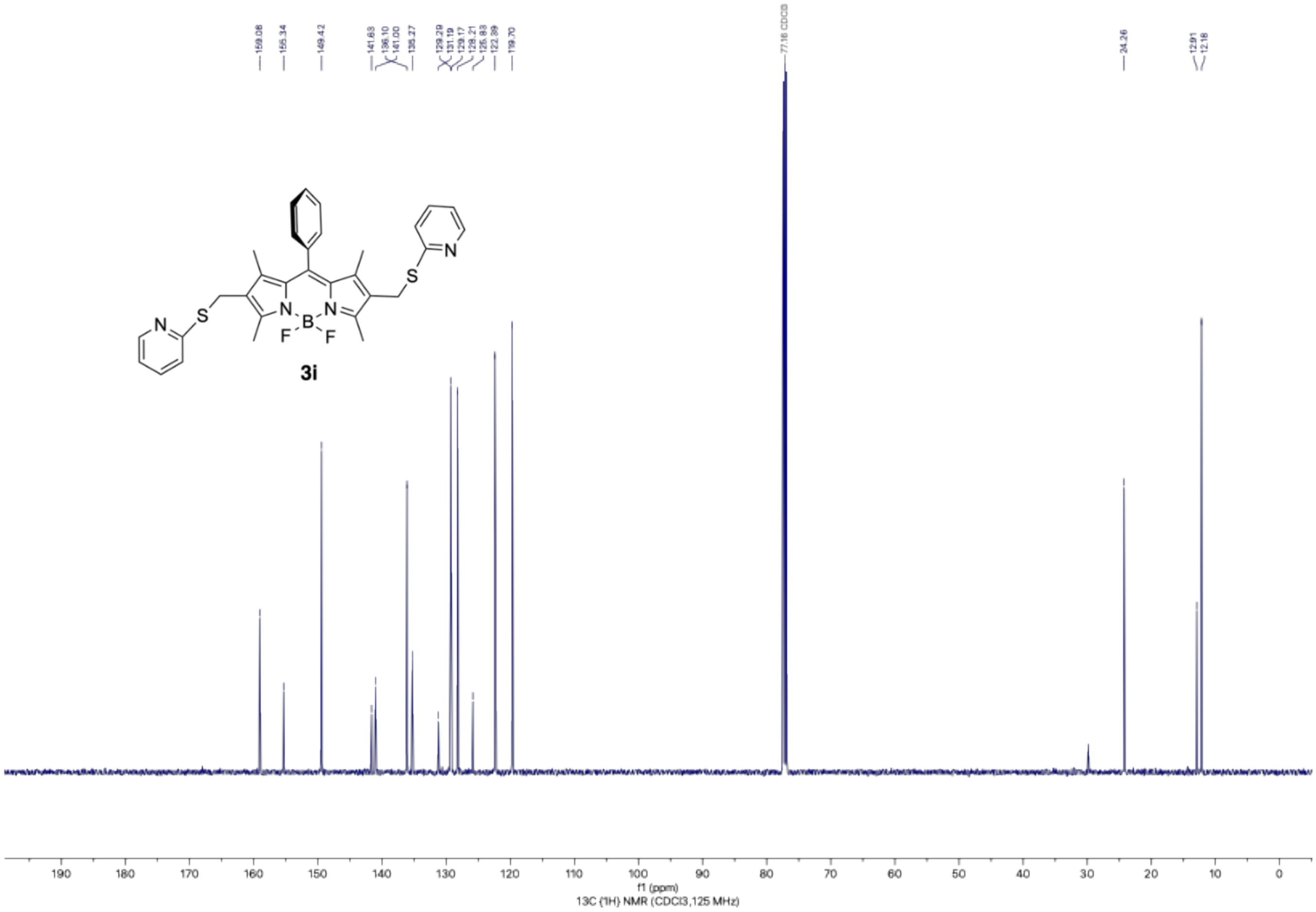

^{13}C {^{1}H}-NMR ($CDCl_3$, 125 MHz) of Compound **3i**

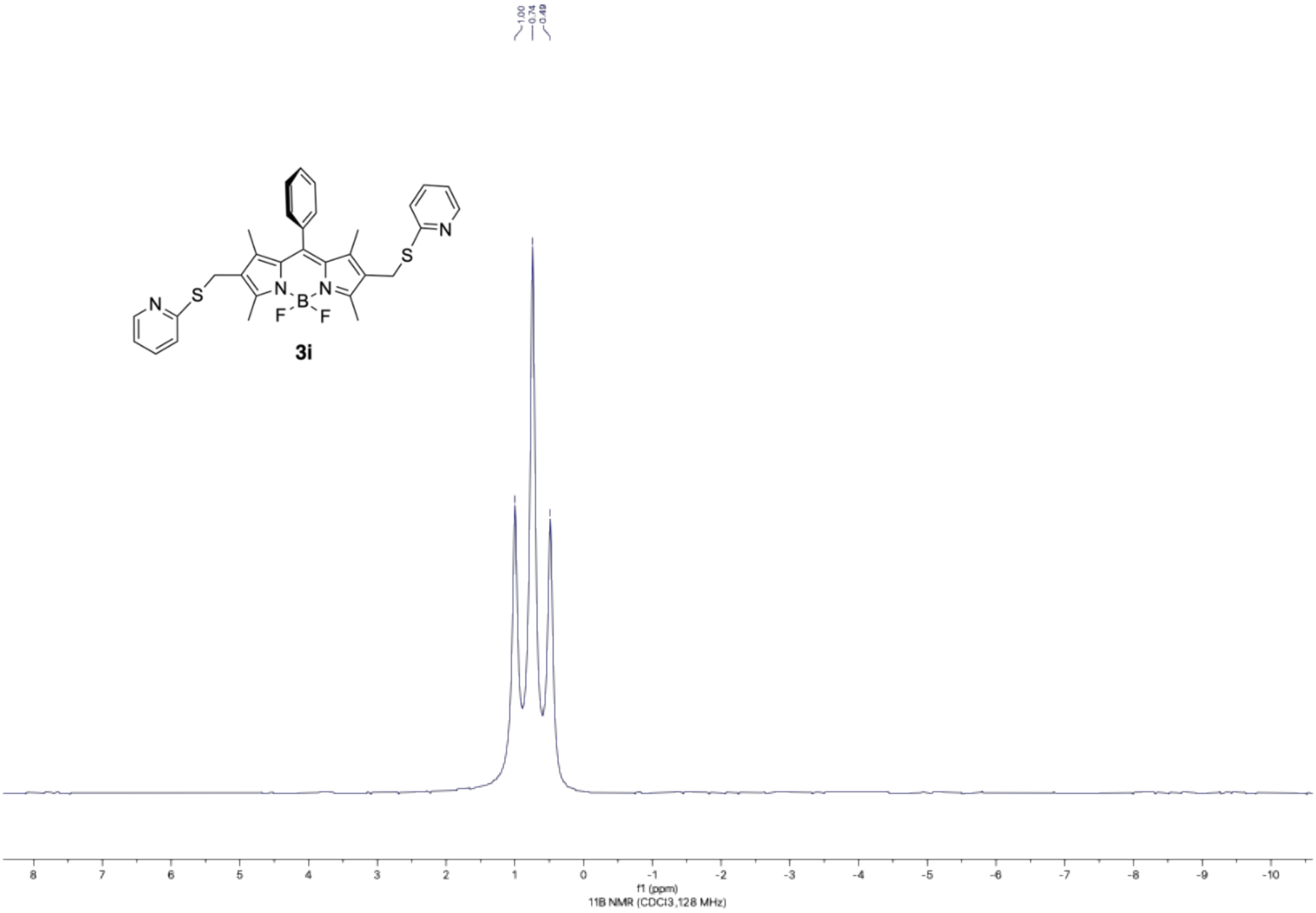

^{11}B-NMR ($CDCl_3$, 128 MHz) of Compound **3i**

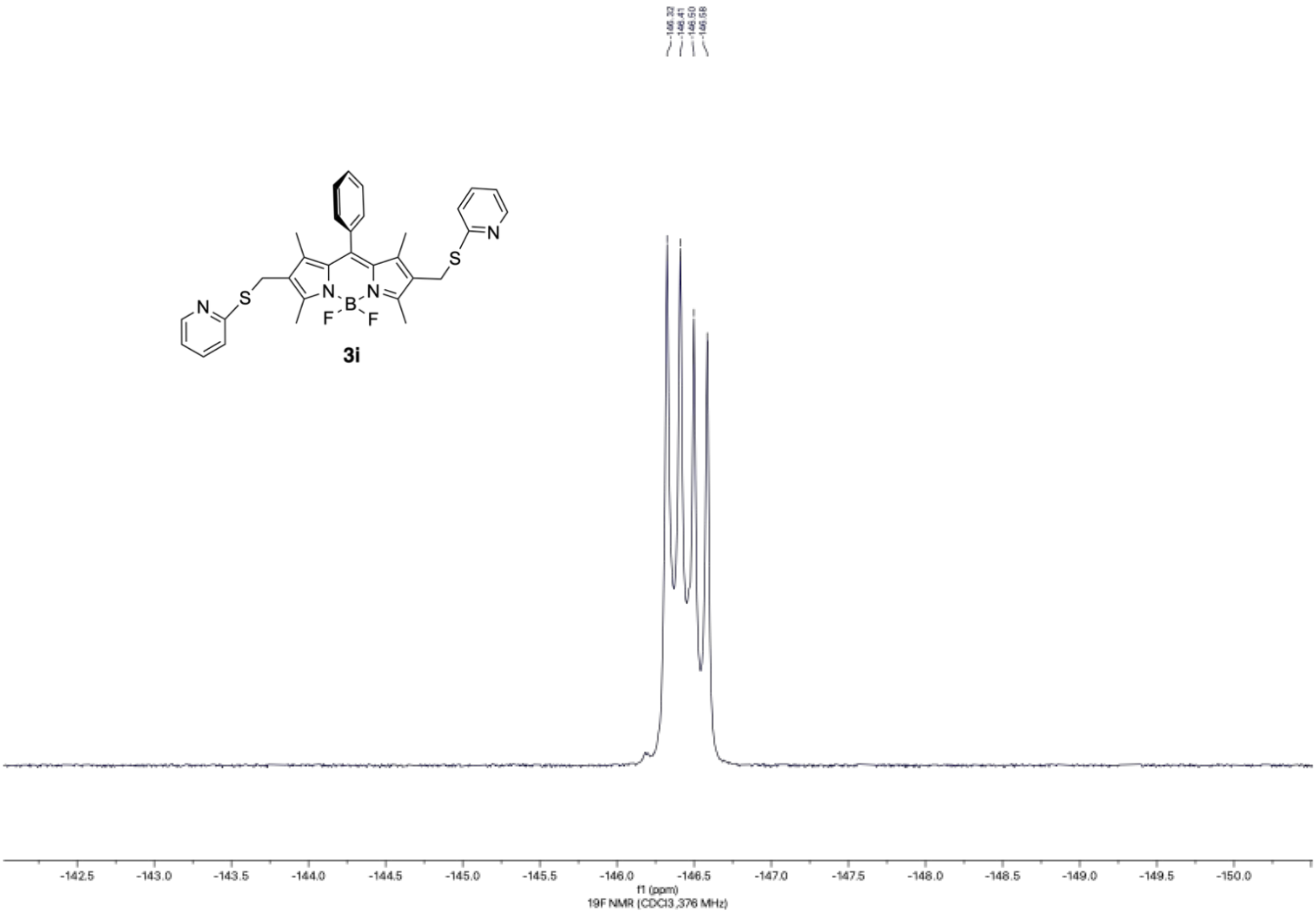

^{19}F-NMR ($CDCl_3$, 376 MHz) of Compound **3i**

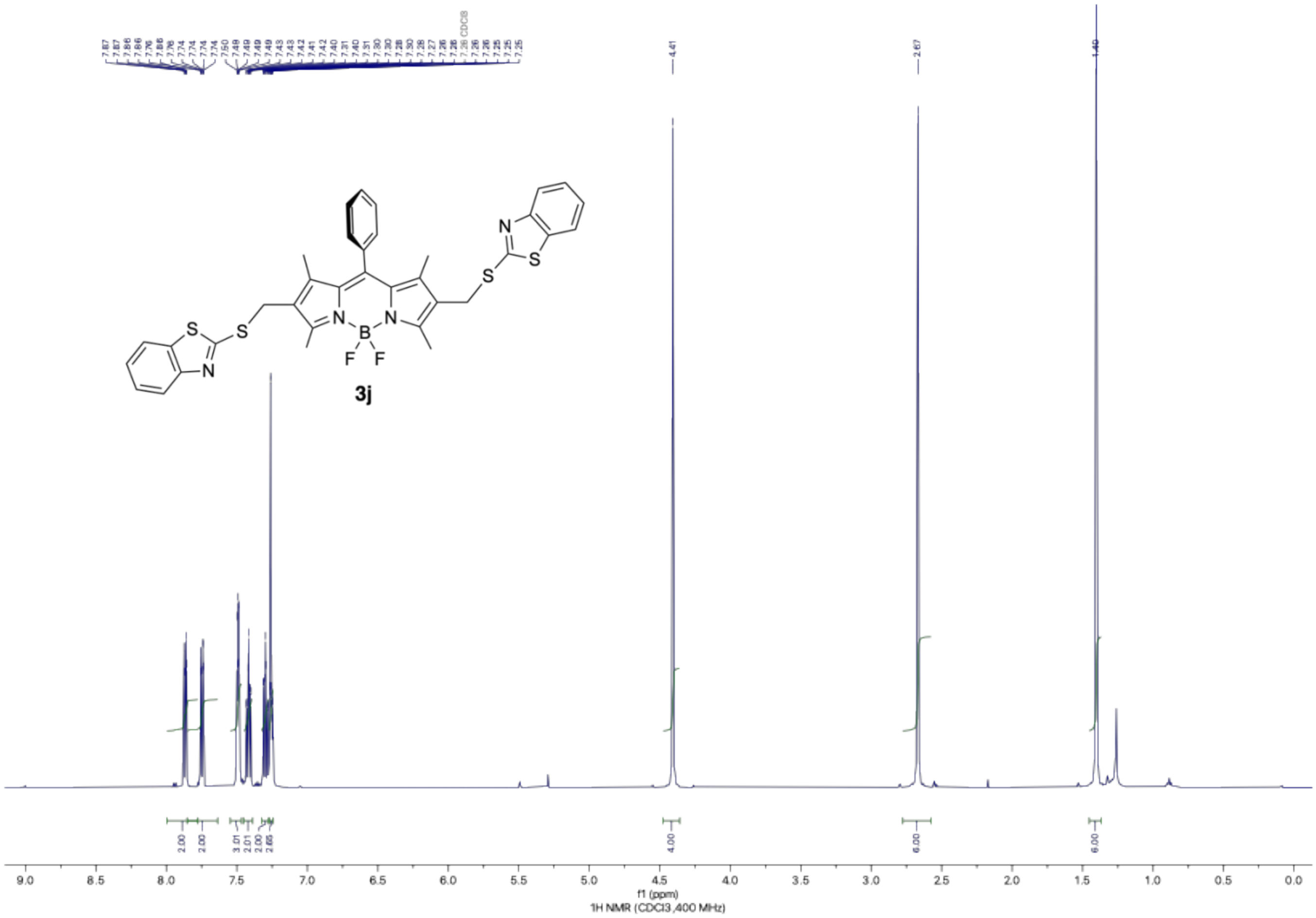

^{1}H-NMR ($CDCl_3$, 400 MHz) of Compound **3j**

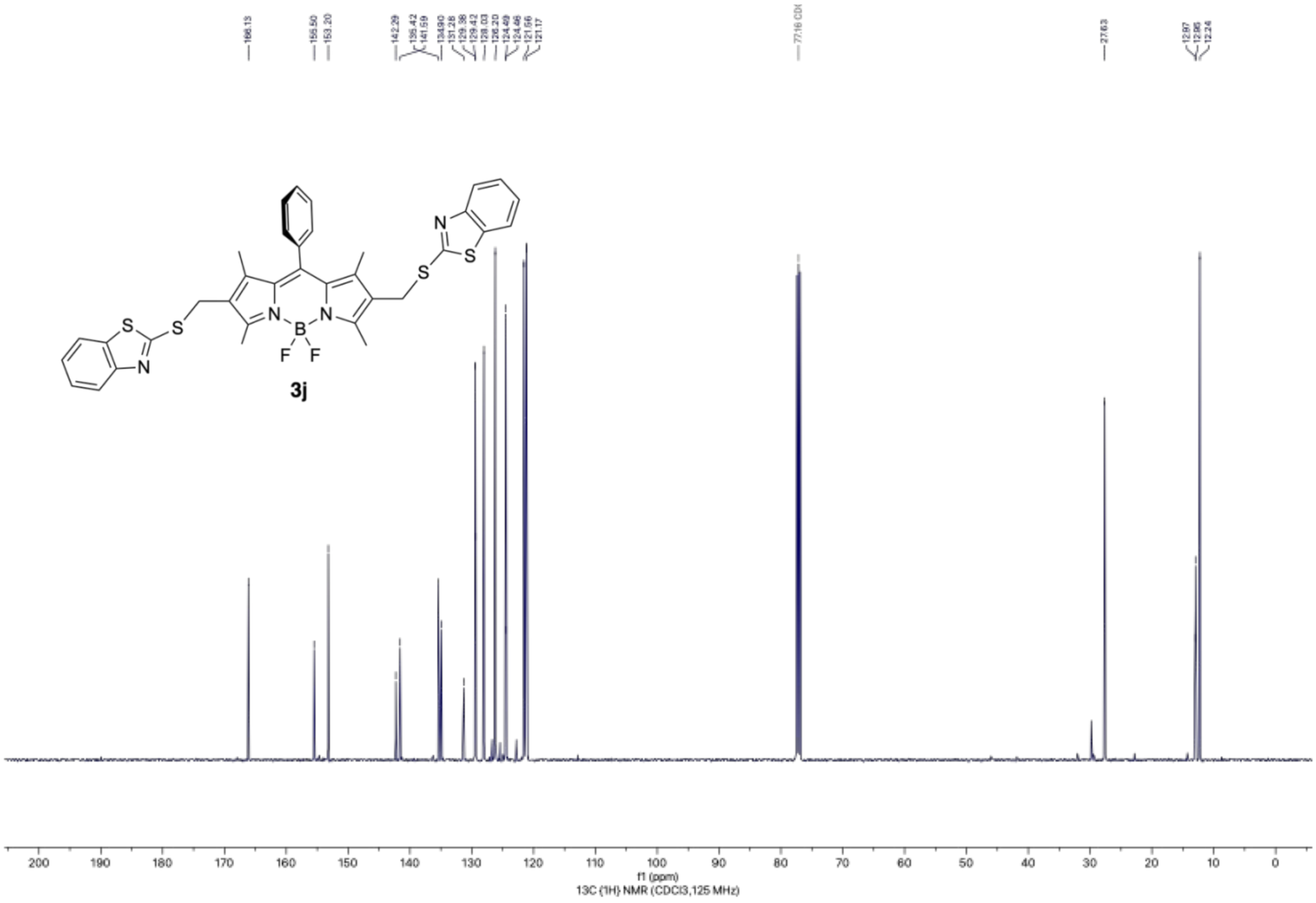

^{13}C {^{1}H}-NMR ($CDCl_3$, 125 MHz) of Compound **3j**

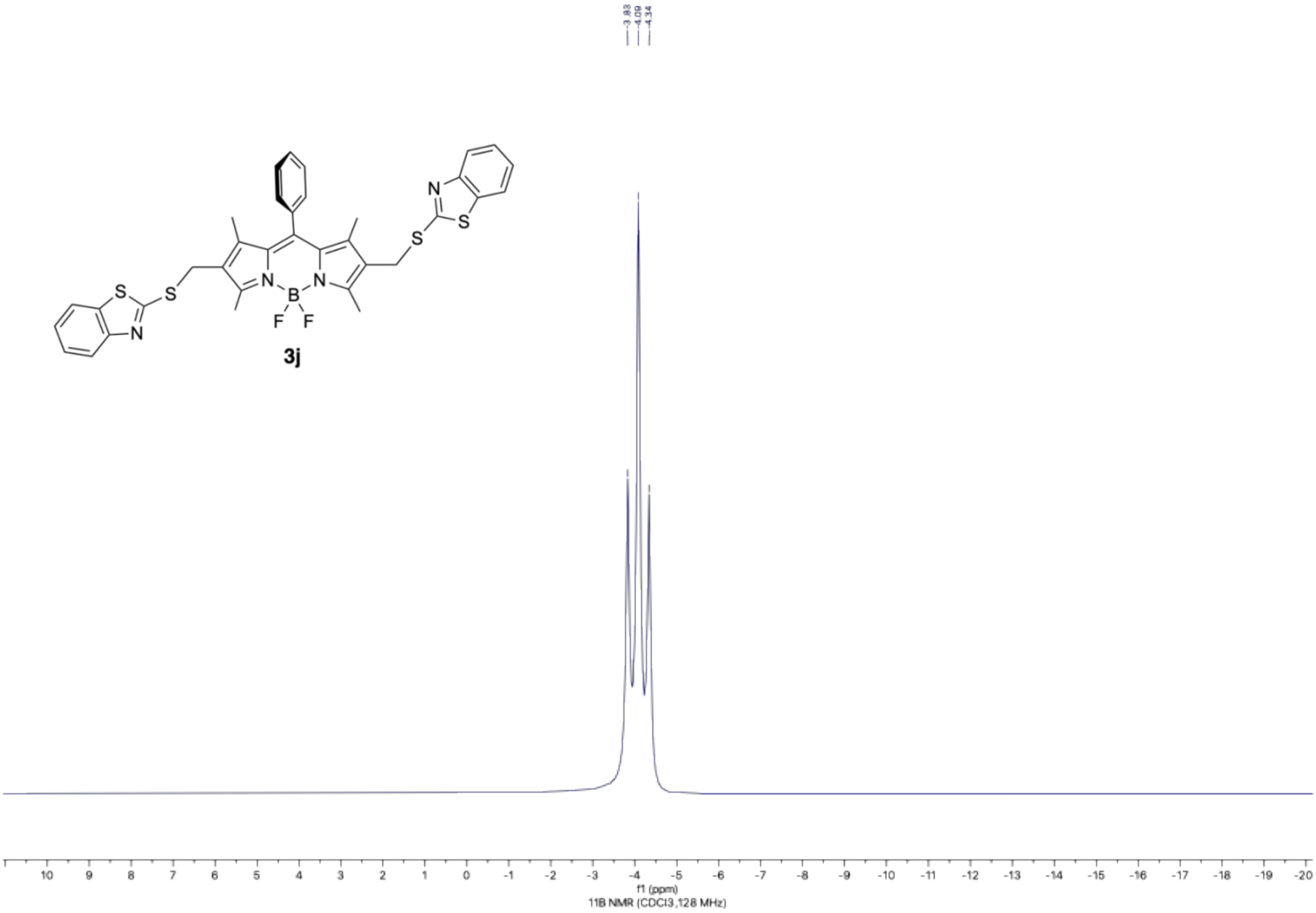

^{11}B-NMR (CDCl$_3$, 128 MHz) of Compound **3j**

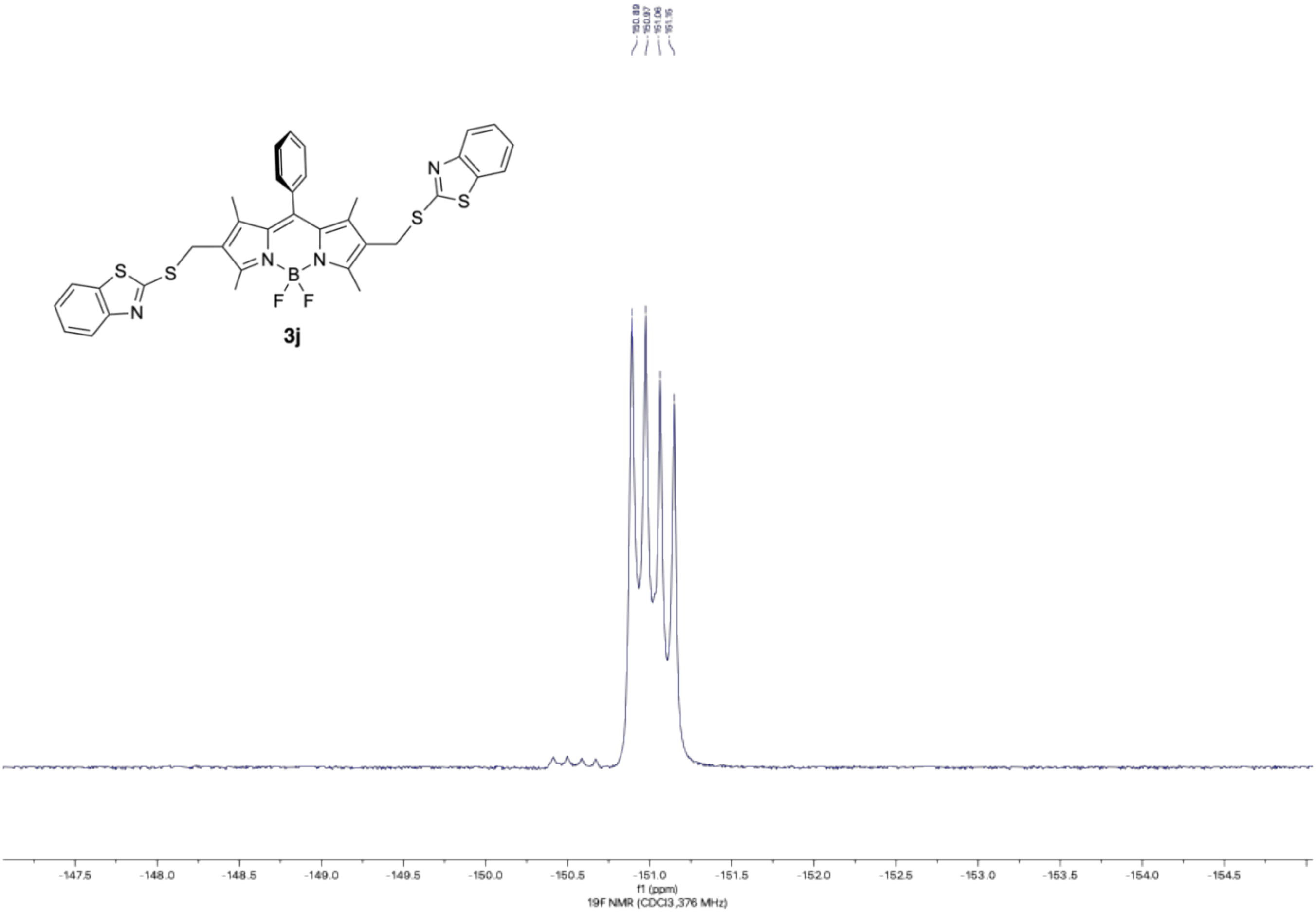

^{19}F-NMR (CDCl$_3$, 376 MHz) of Compound **3j**

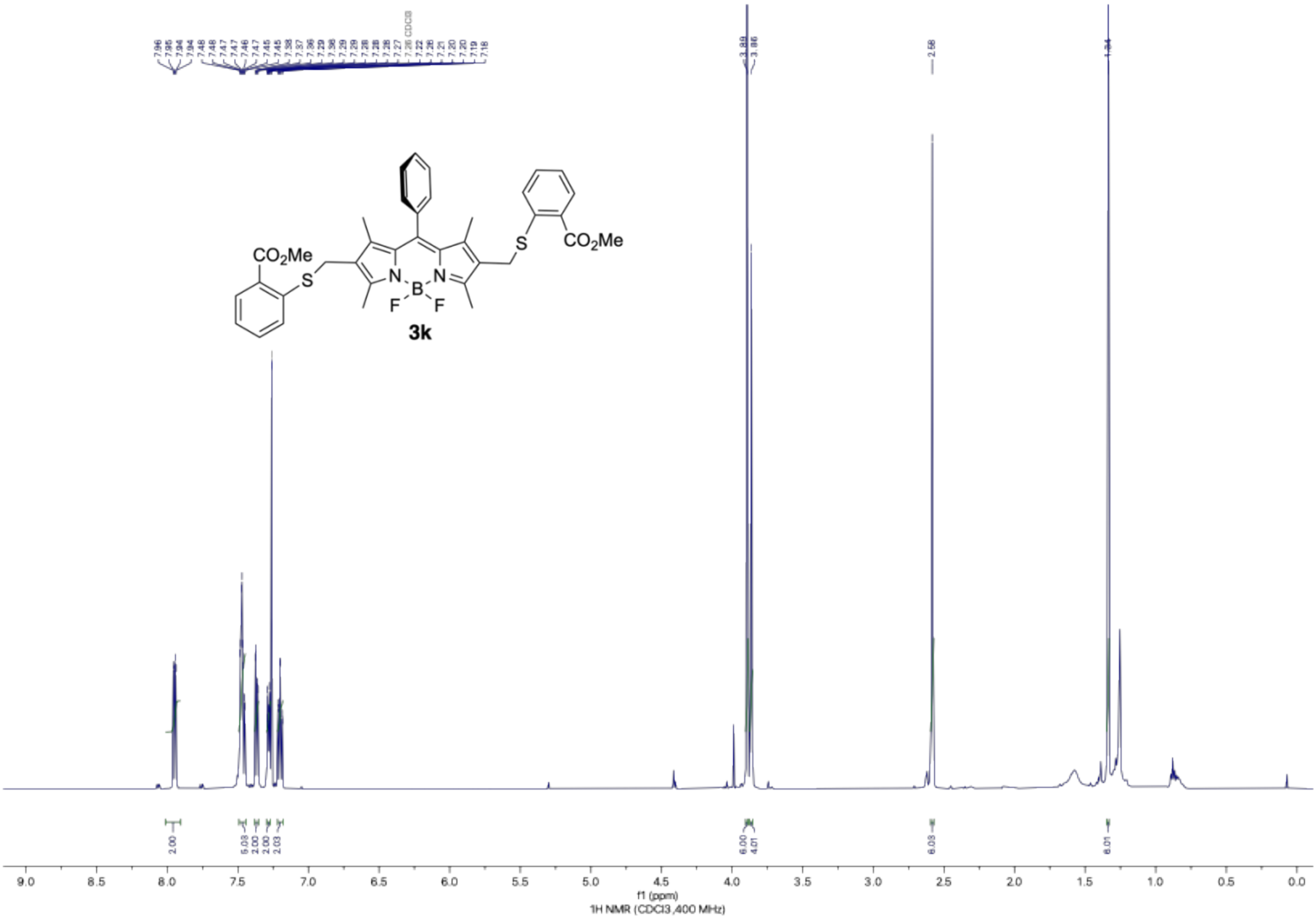

^{1}H-NMR ($CDCl_3$, 400 MHz) of Compound **3k**

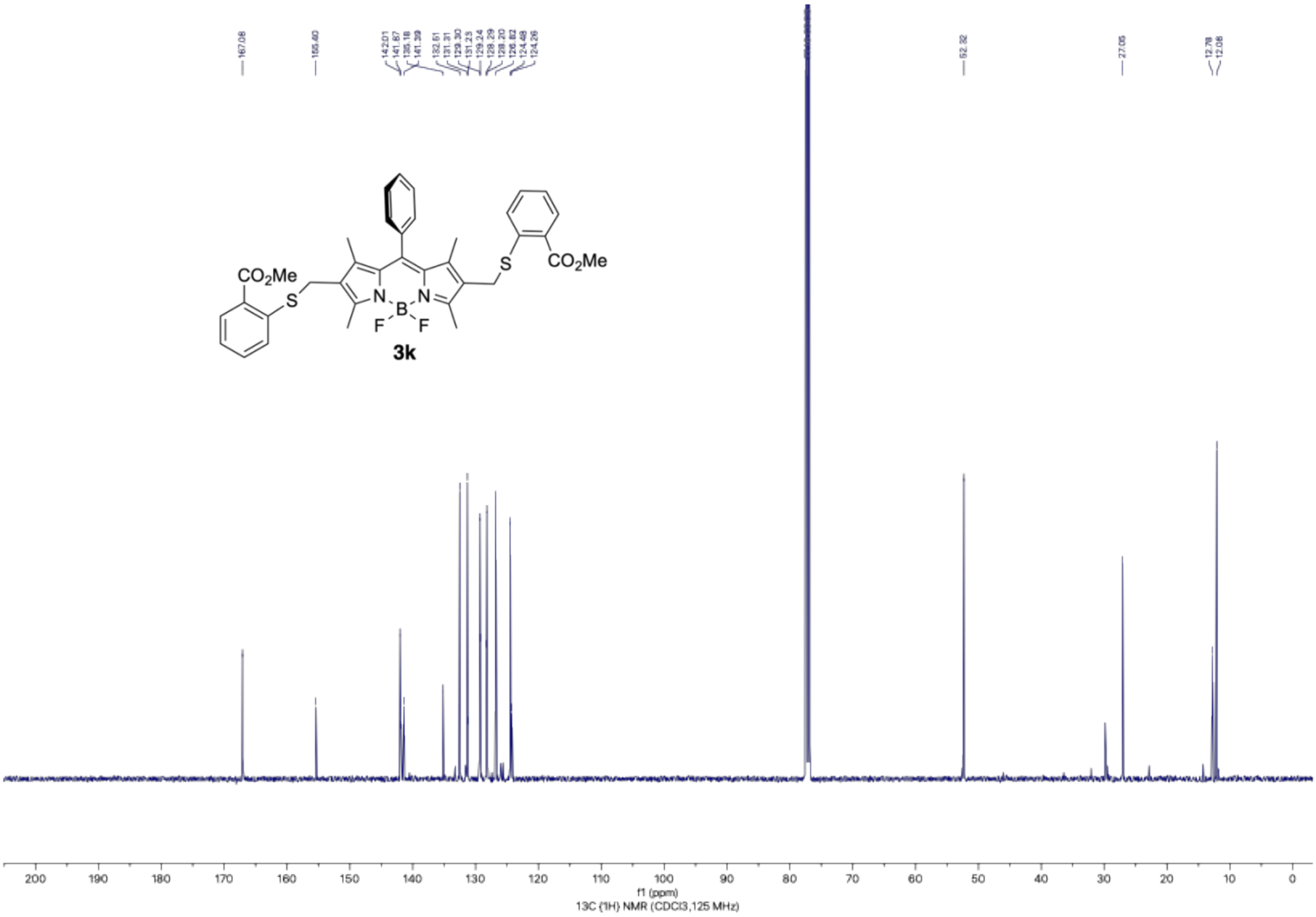

^{13}C {^{1}H}-NMR ($CDCl_3$, 125 MHz) of Compound **3k**

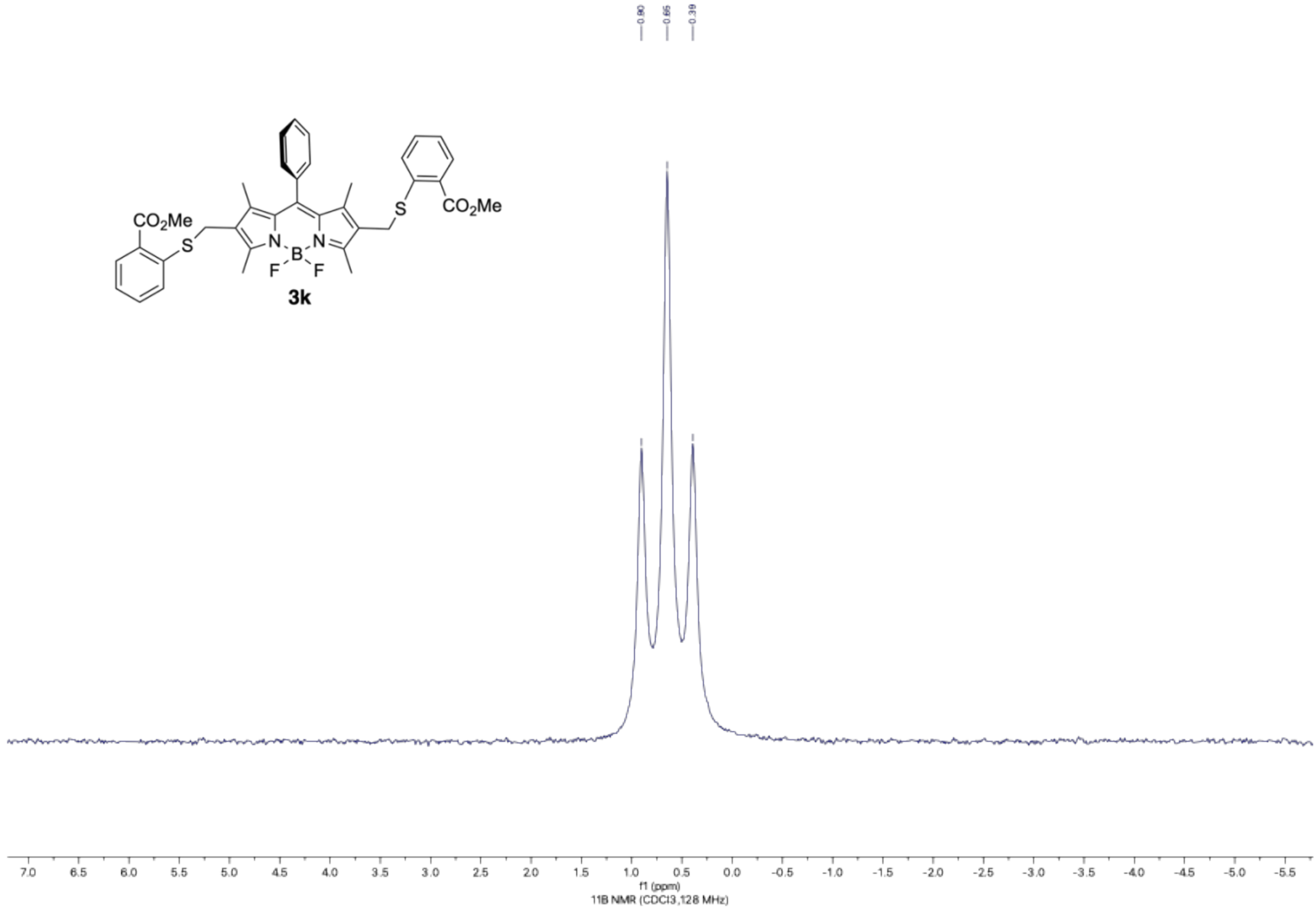

^{11}B-NMR ($CDCl_3$, 128 MHz) of Compound **3k**

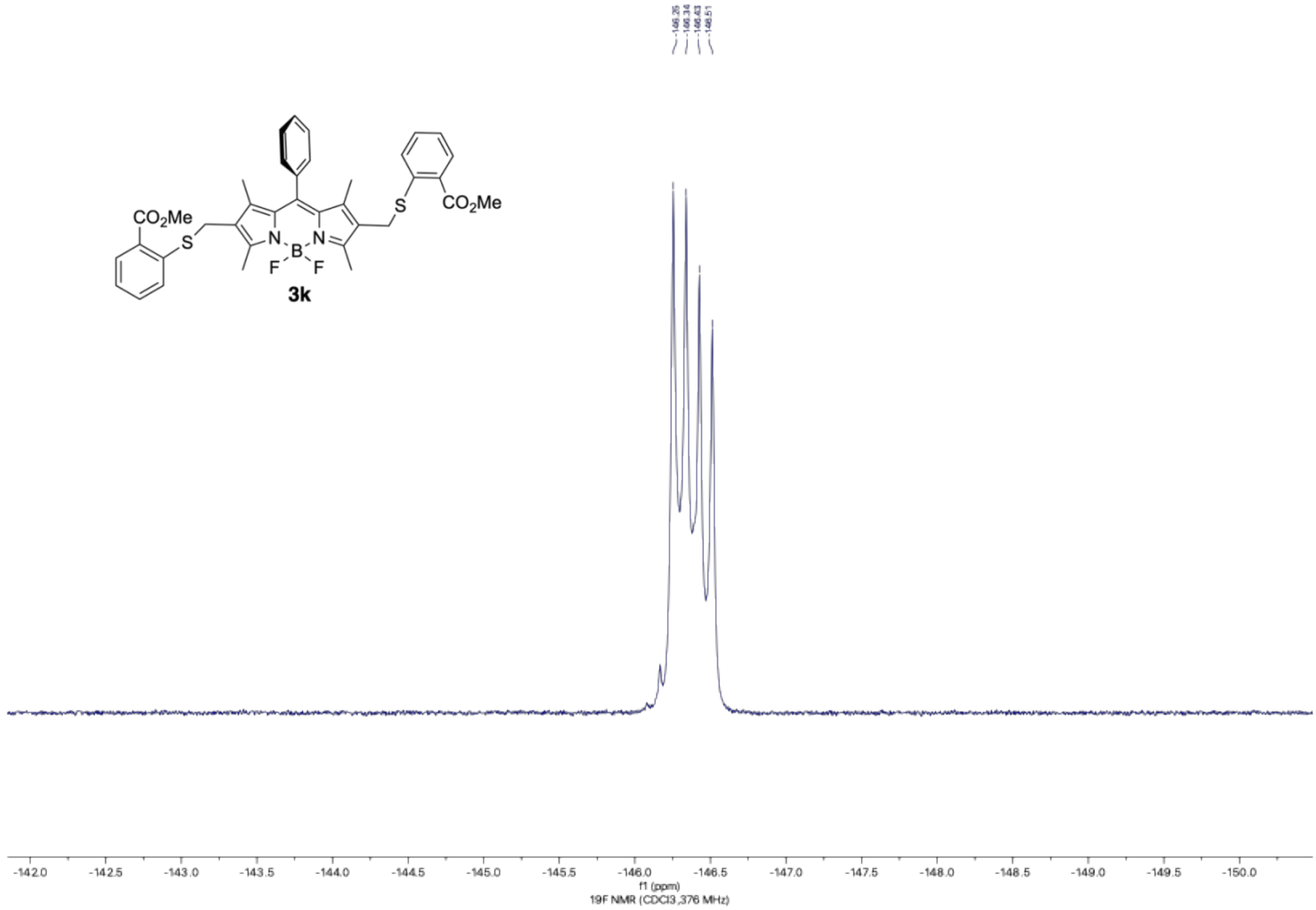

^{19}F-NMR ($CDCl_3$, 376 MHz) of Compound **3k**

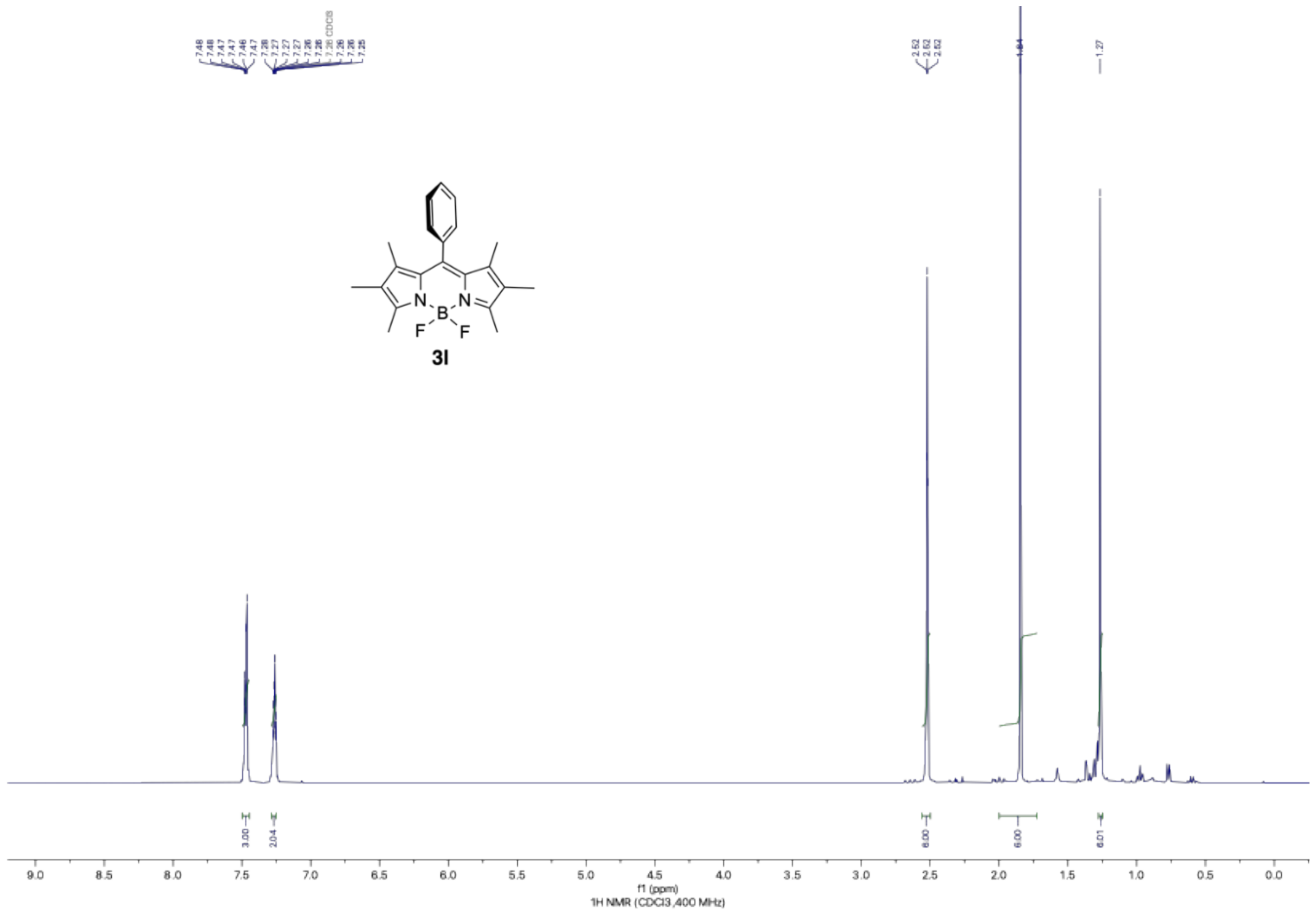

^{1}H-NMR ($CDCl_3$, 400 MHz) of Compound **3l**

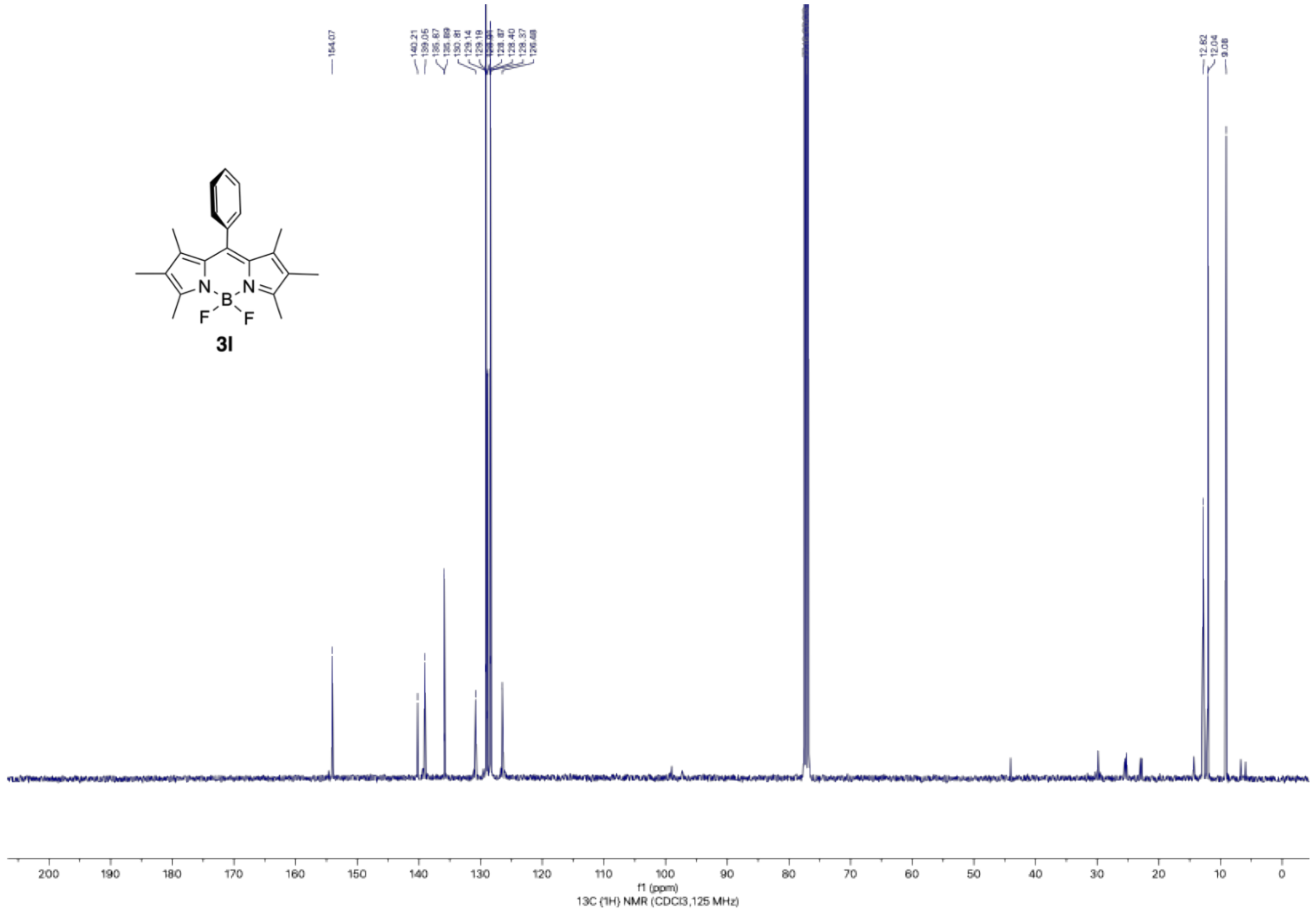

^{13}C {^{1}H}-NMR ($CDCl_3$, 125 MHz) of Compound **3l**

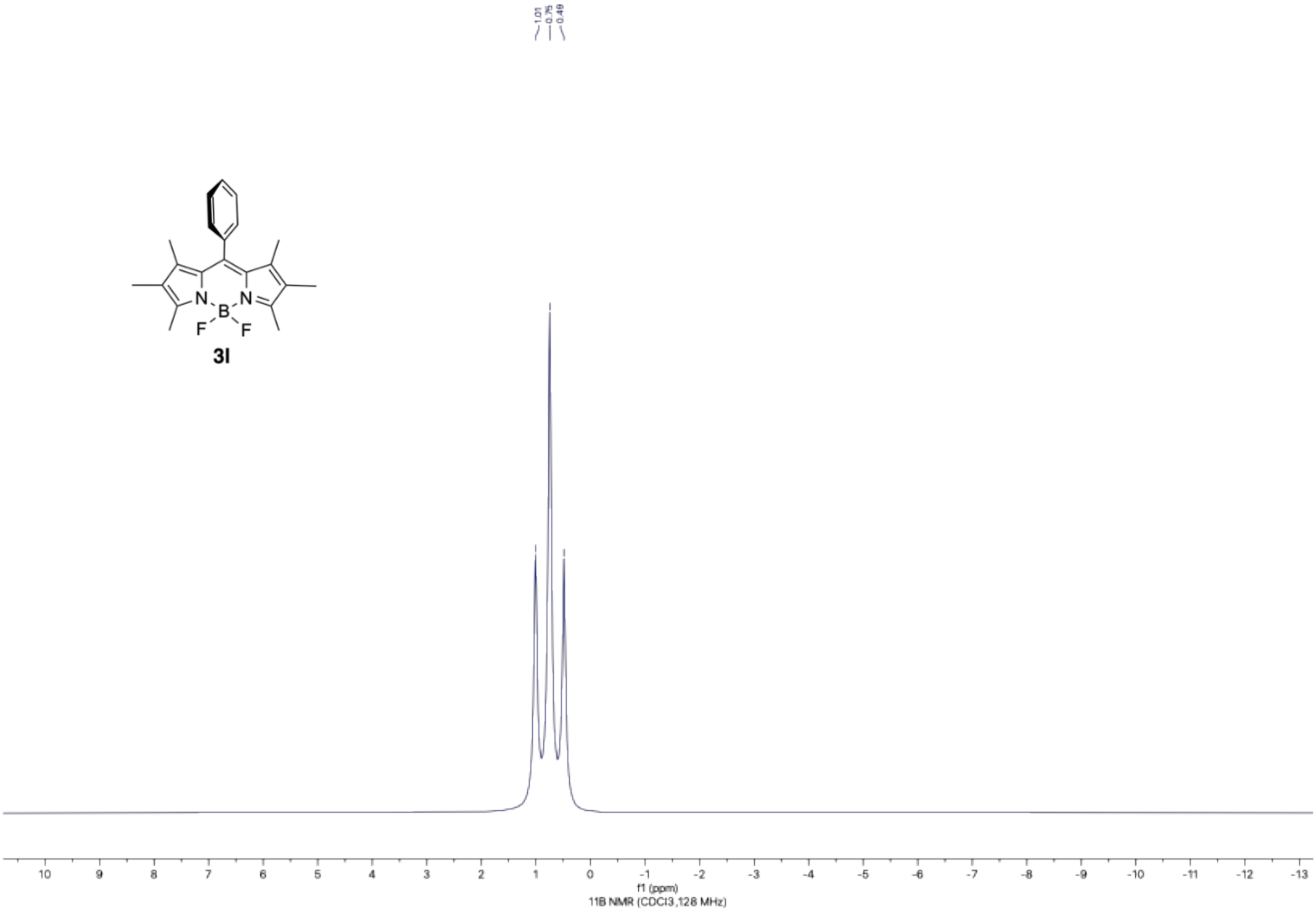

^{11}B-NMR ($CDCl_3$, 128 MHz) of Compound **3l**

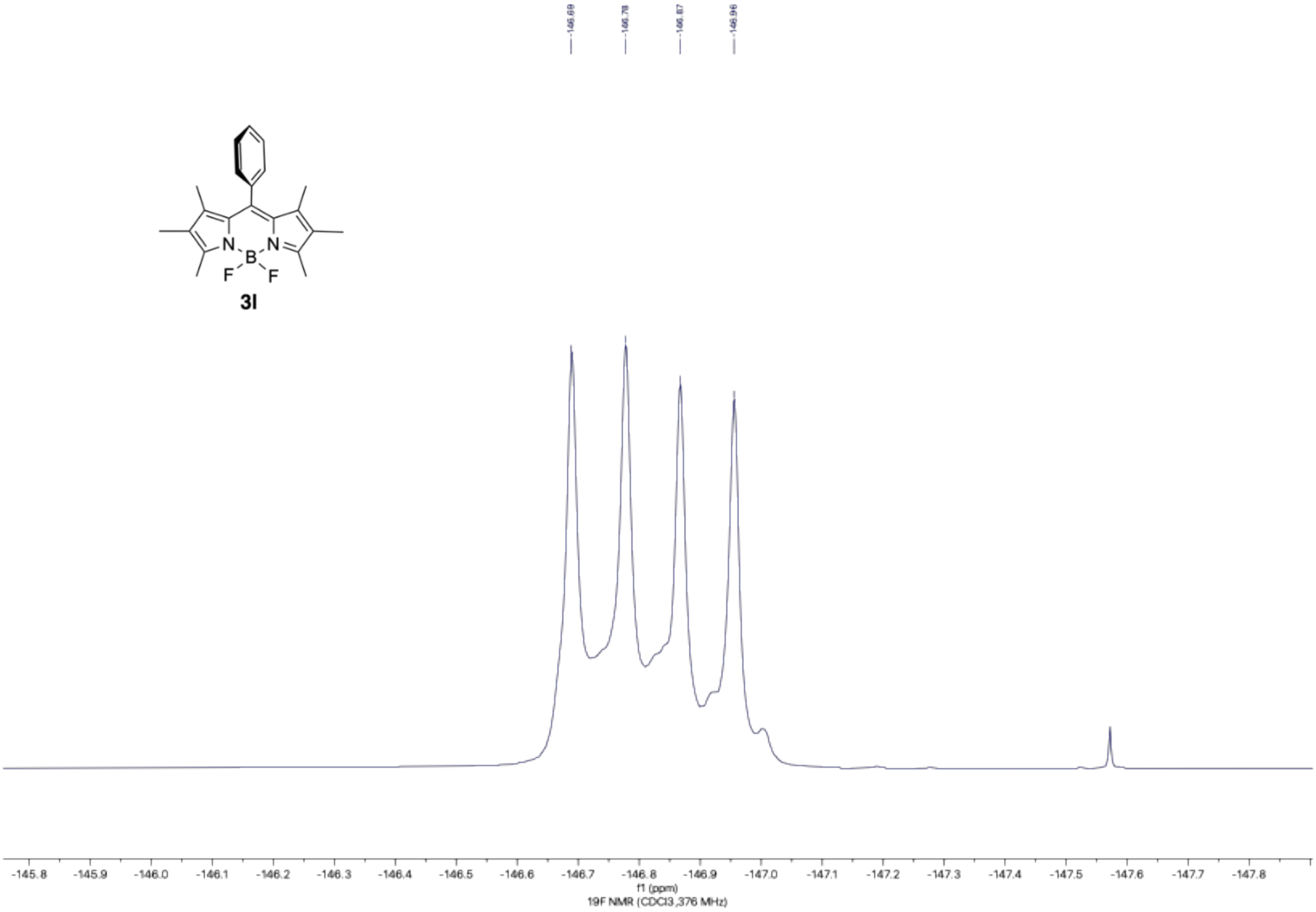

^{19}F-NMR ($CDCl_3$, 376 MHz) of Compound **3l**

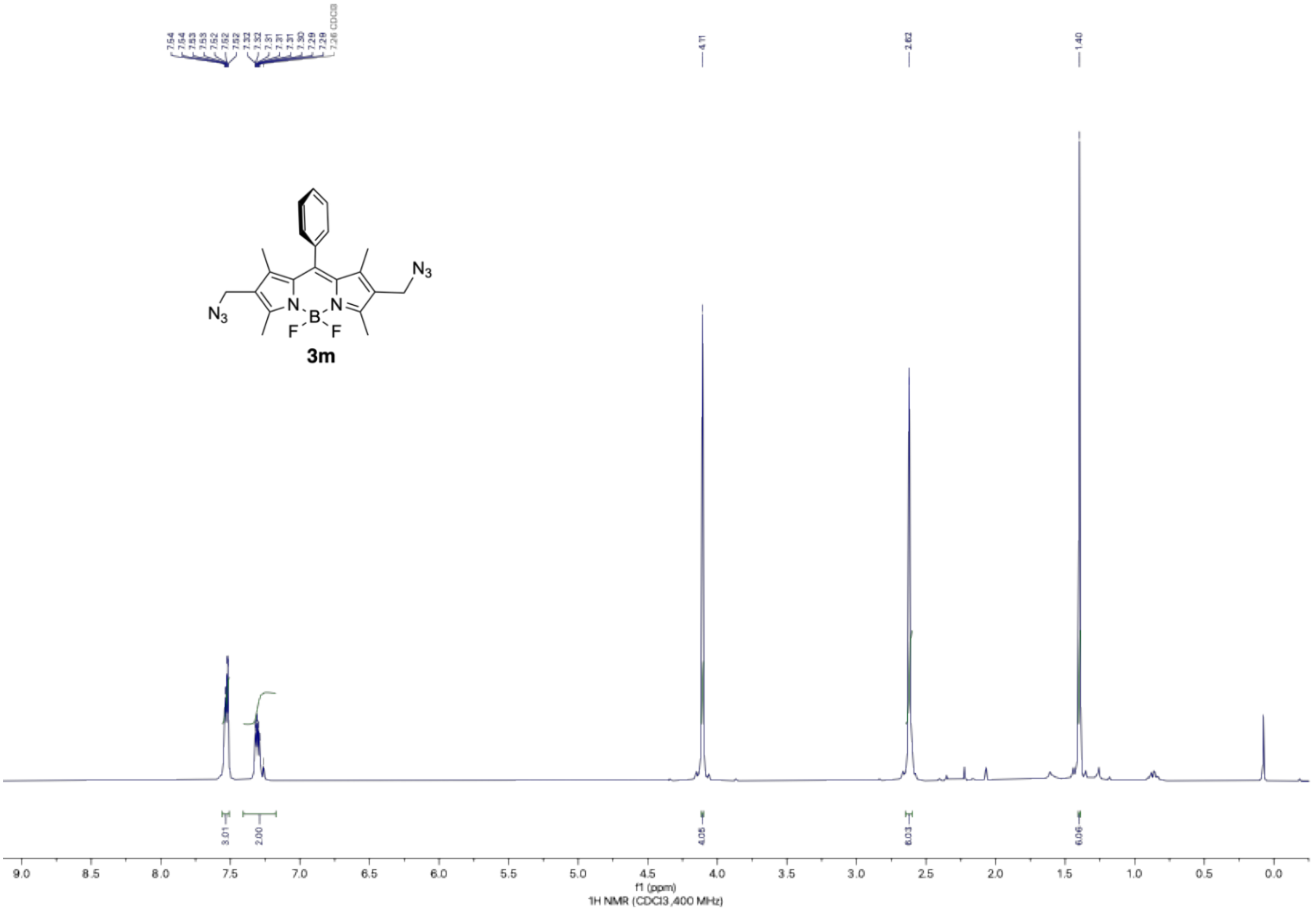

^{1}H-NMR ($CDCl_3$, 400 MHz) of Compound **3m**

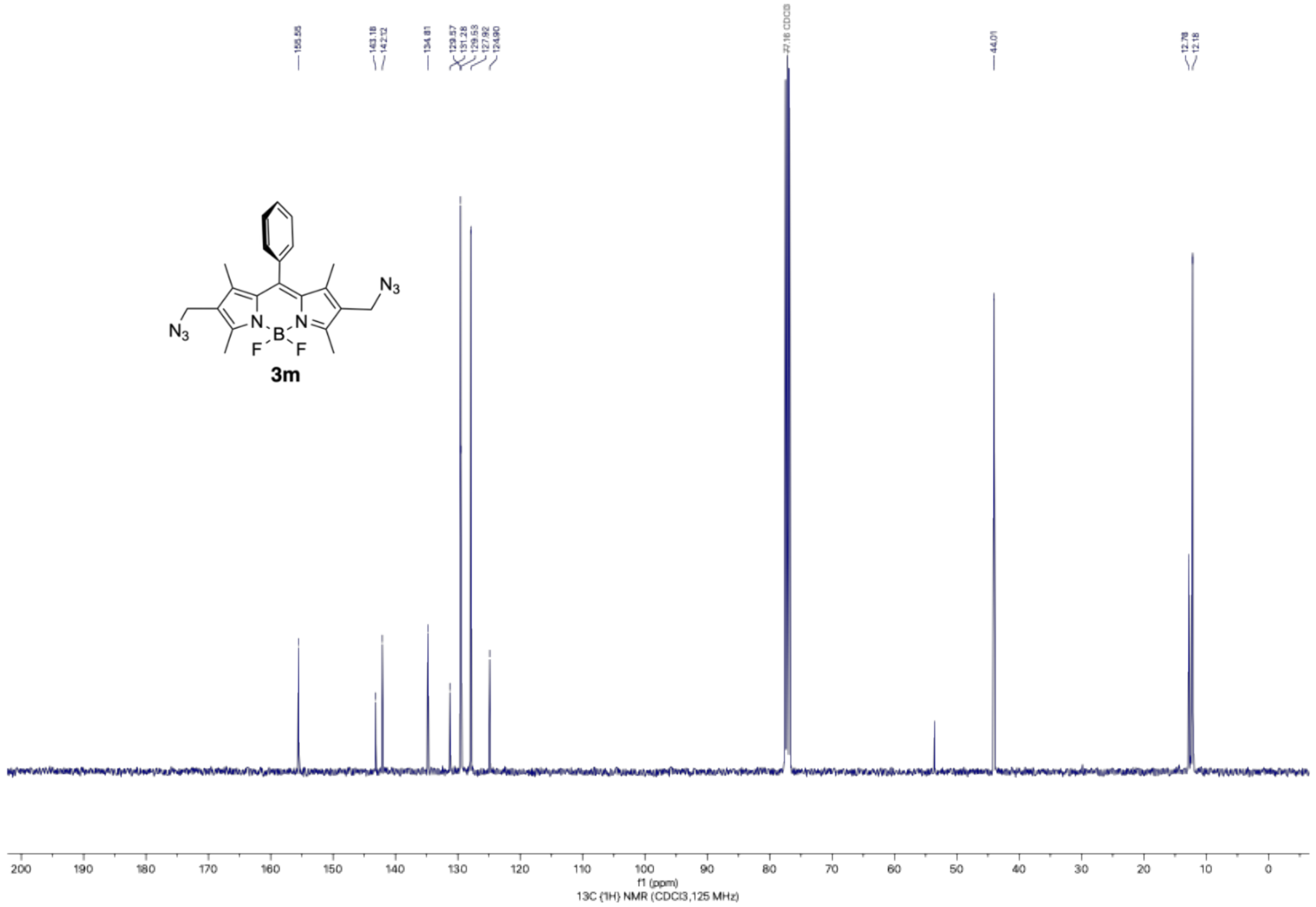

^{13}C {^{1}H}-NMR ($CDCl_3$, 125 MHz) of Compound **3m**

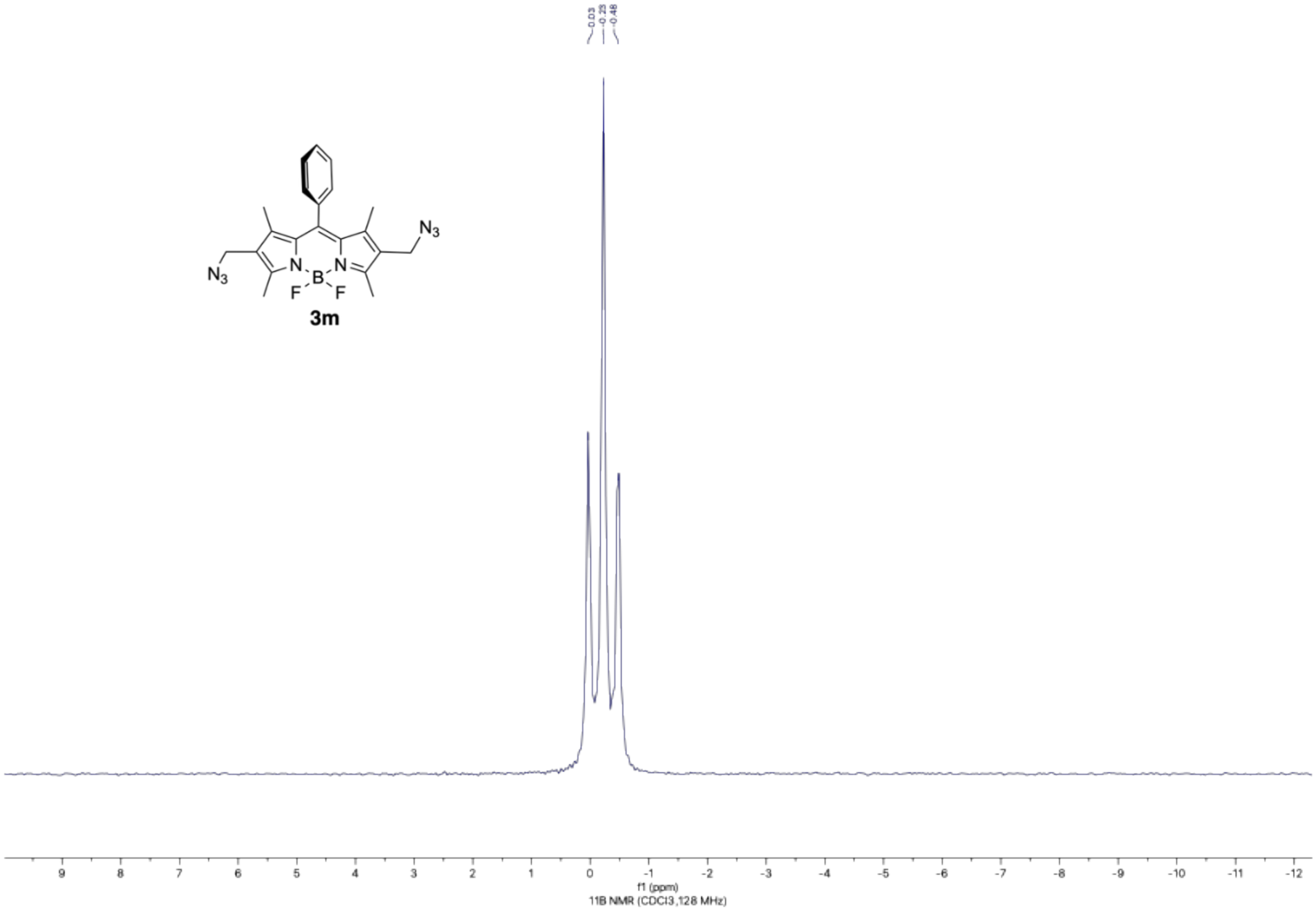

^{11}B-NMR ($CDCl_3$, 128 MHz) of Compound **3m**

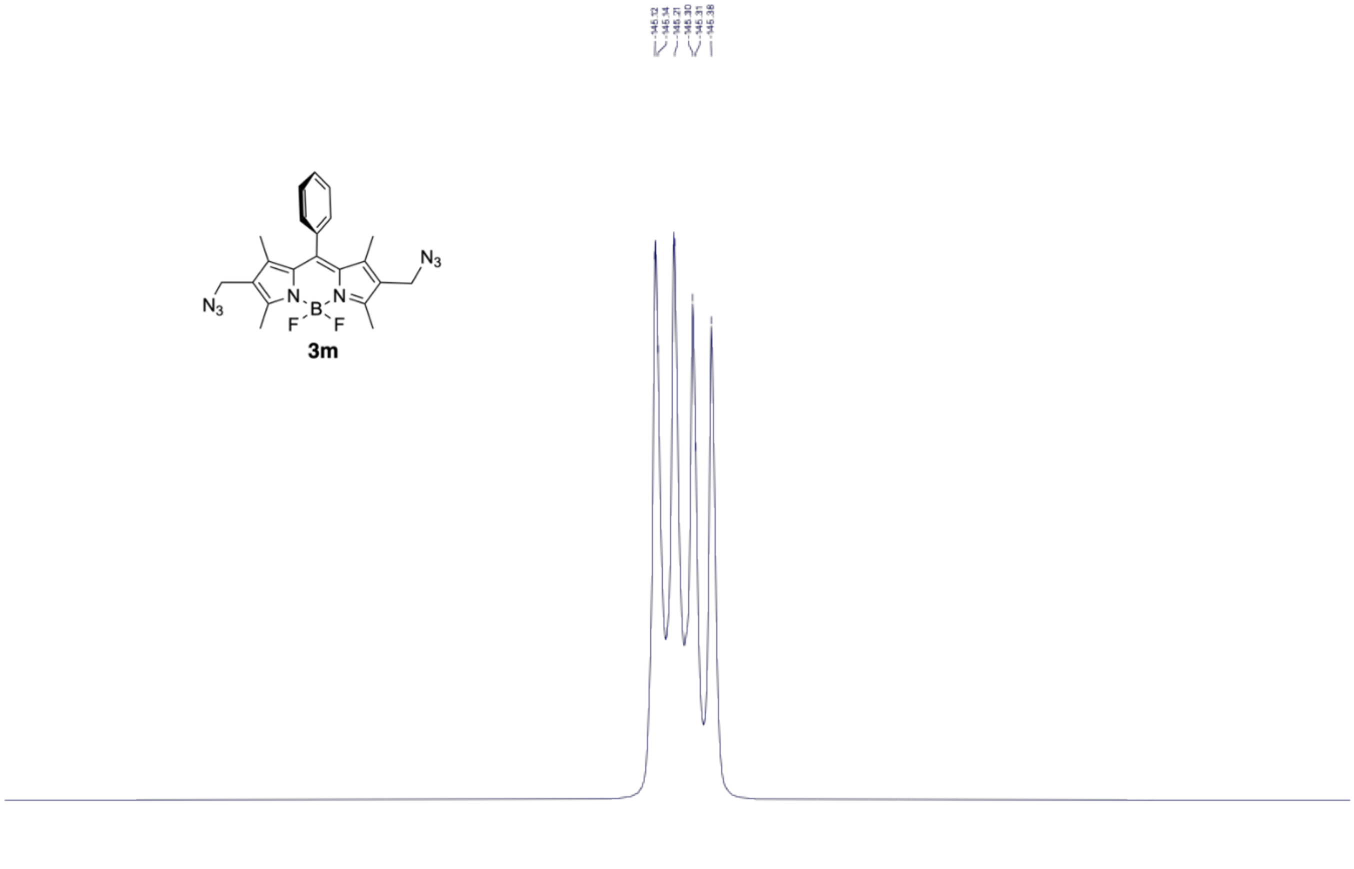

^{19}F-NMR ($CDCl_3$, 376 MHz) of Compound **3m**

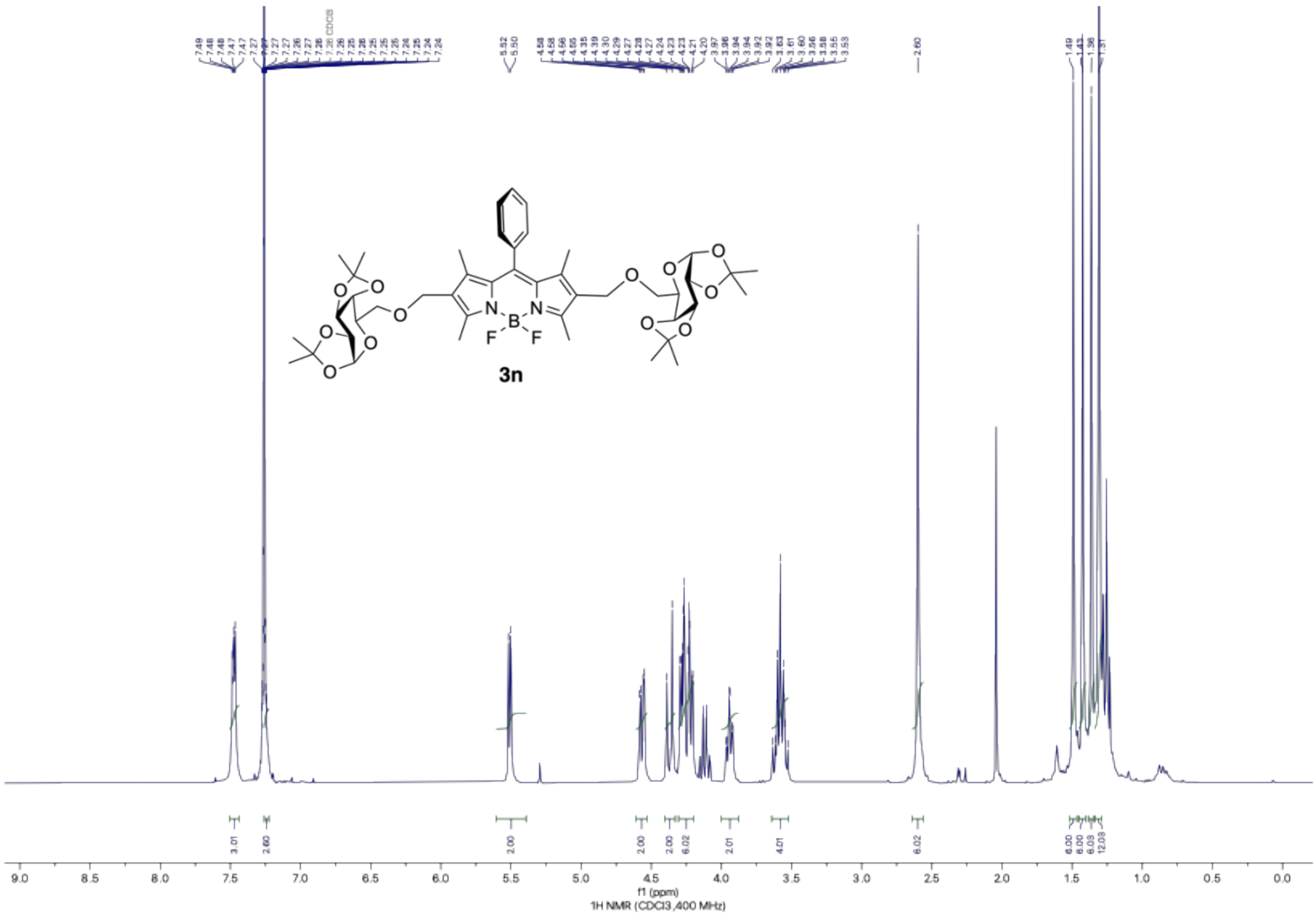

^{1}H-NMR ($CDCl_3$, 400 MHz) of Compound **3n**

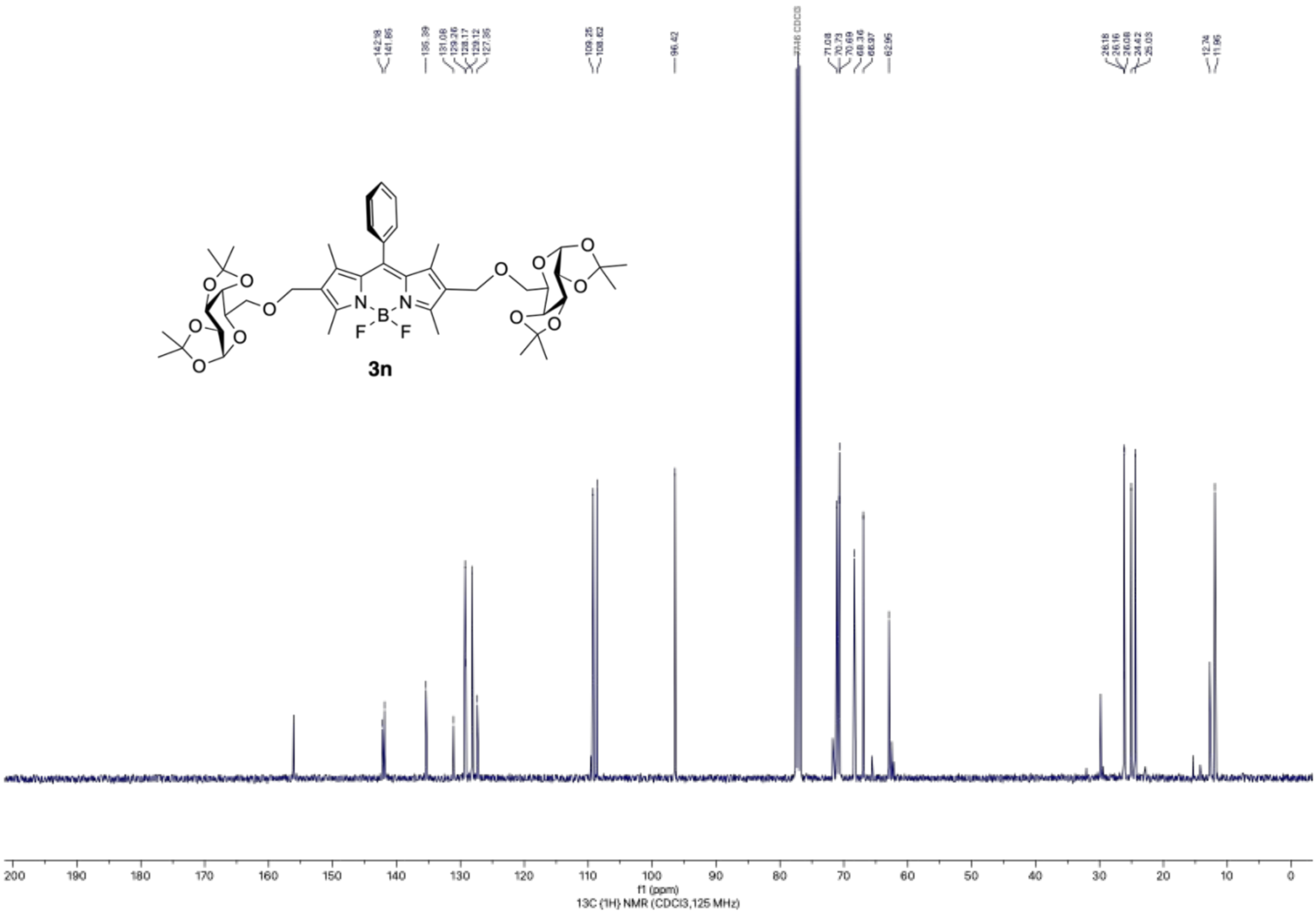

^{13}C {^{1}H}-NMR ($CDCl_3$, 125 MHz) of Compound **3n**

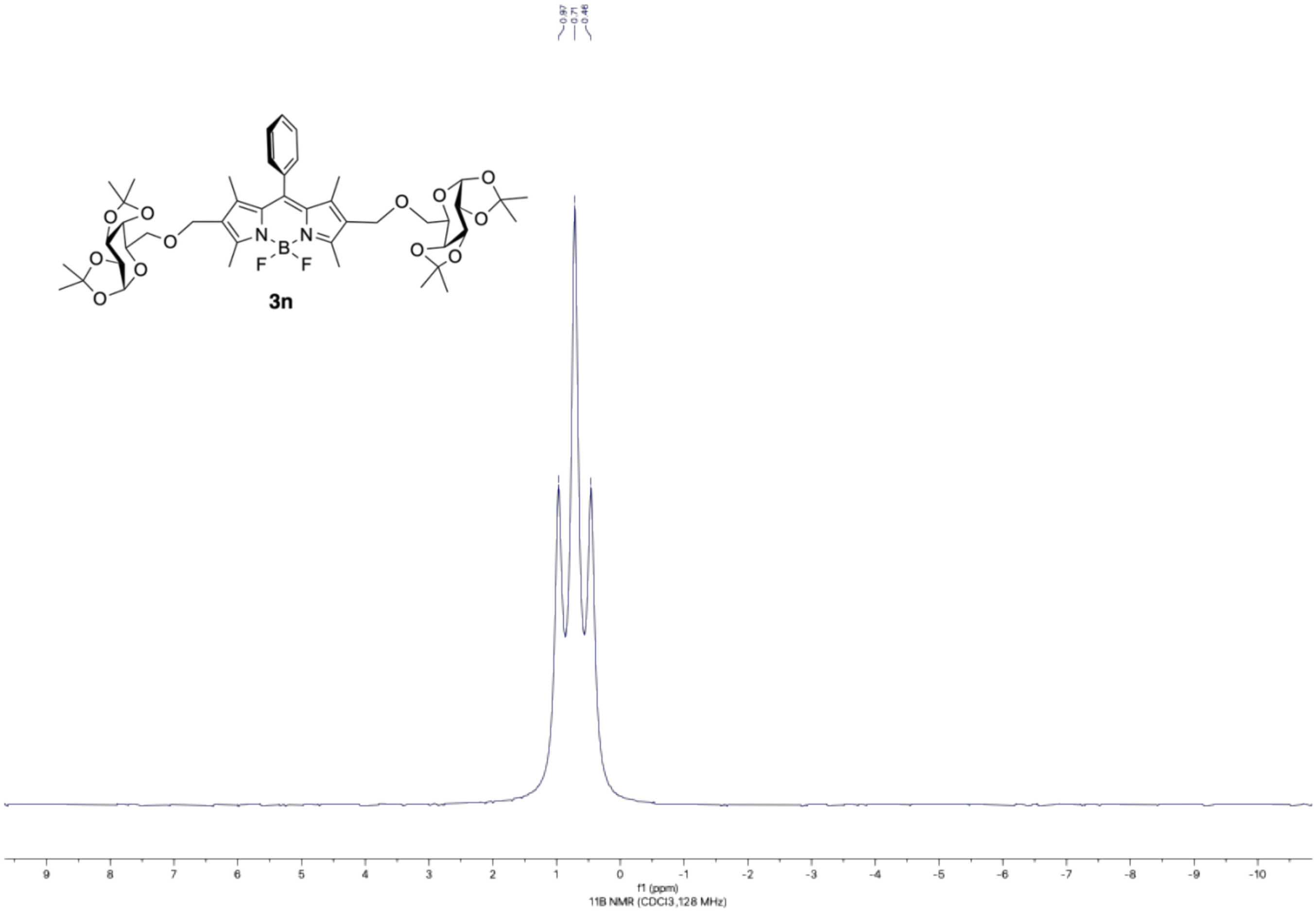

^{11}B-NMR ($CDCl_3$, 128 MHz) of Compound **3n**

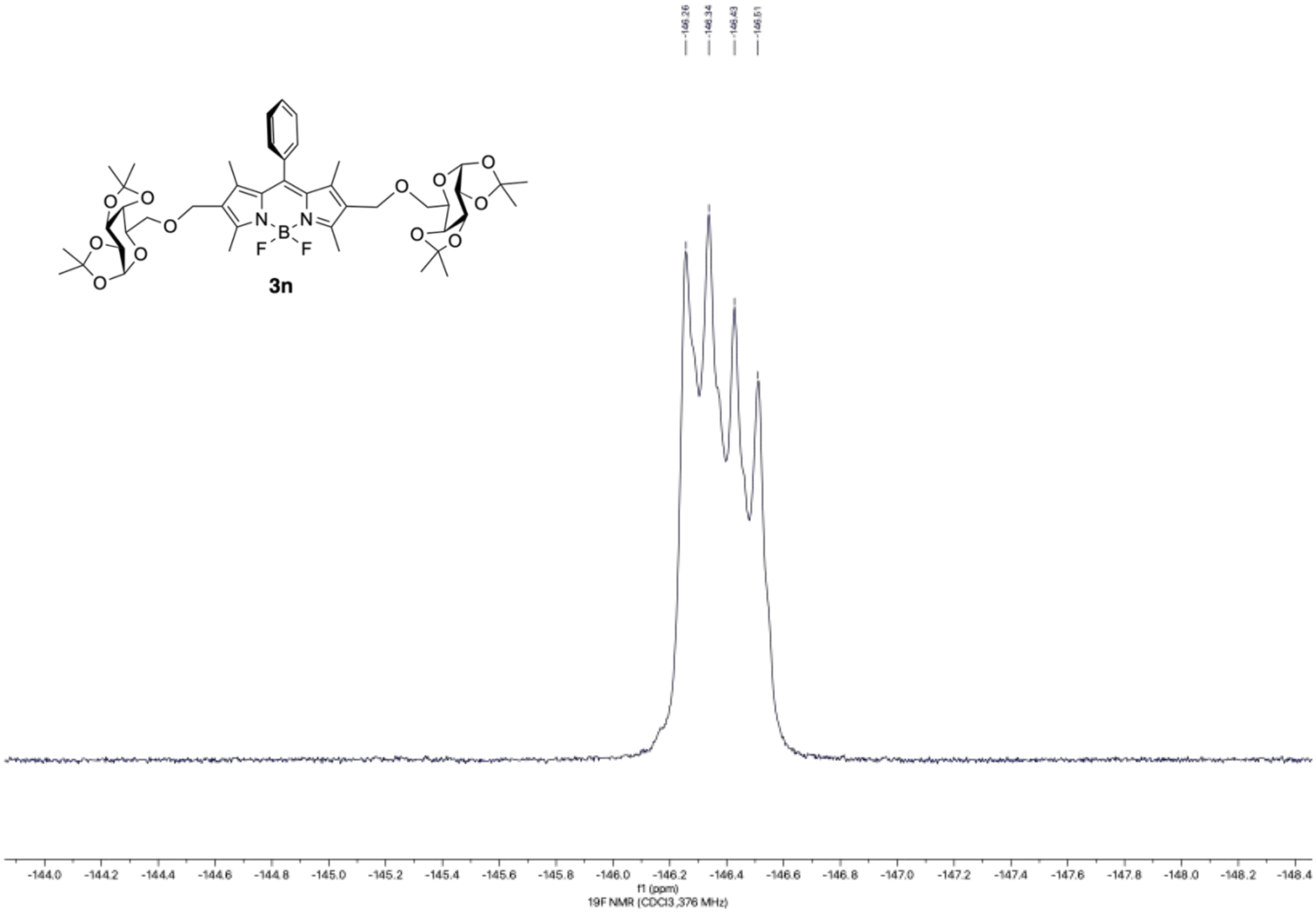

^{19}F-NMR ($CDCl_3$, 376 MHz) of Compound **3n**

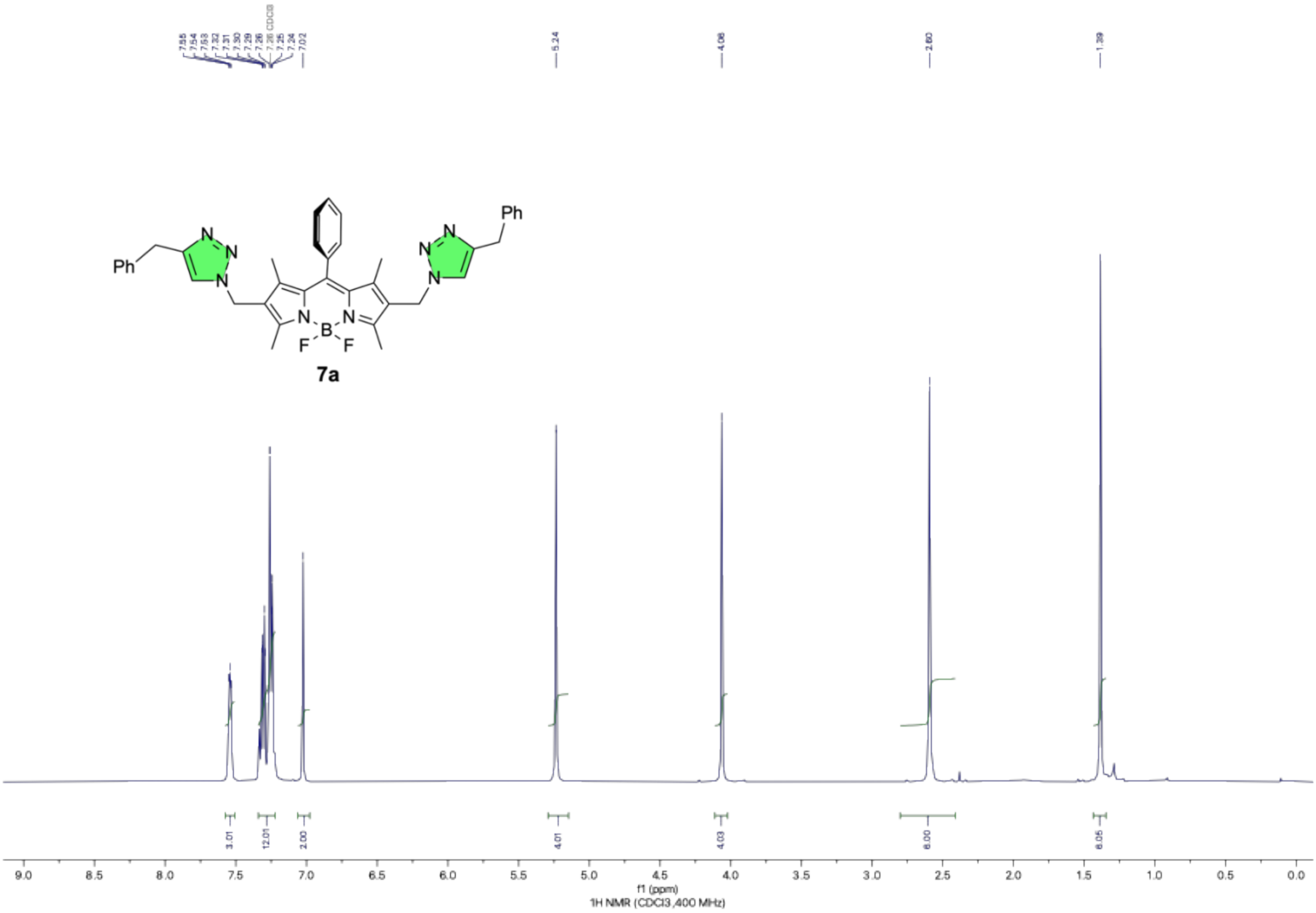

^{1}H-NMR ($CDCl_3$, 400 MHz) of Compound **7a**

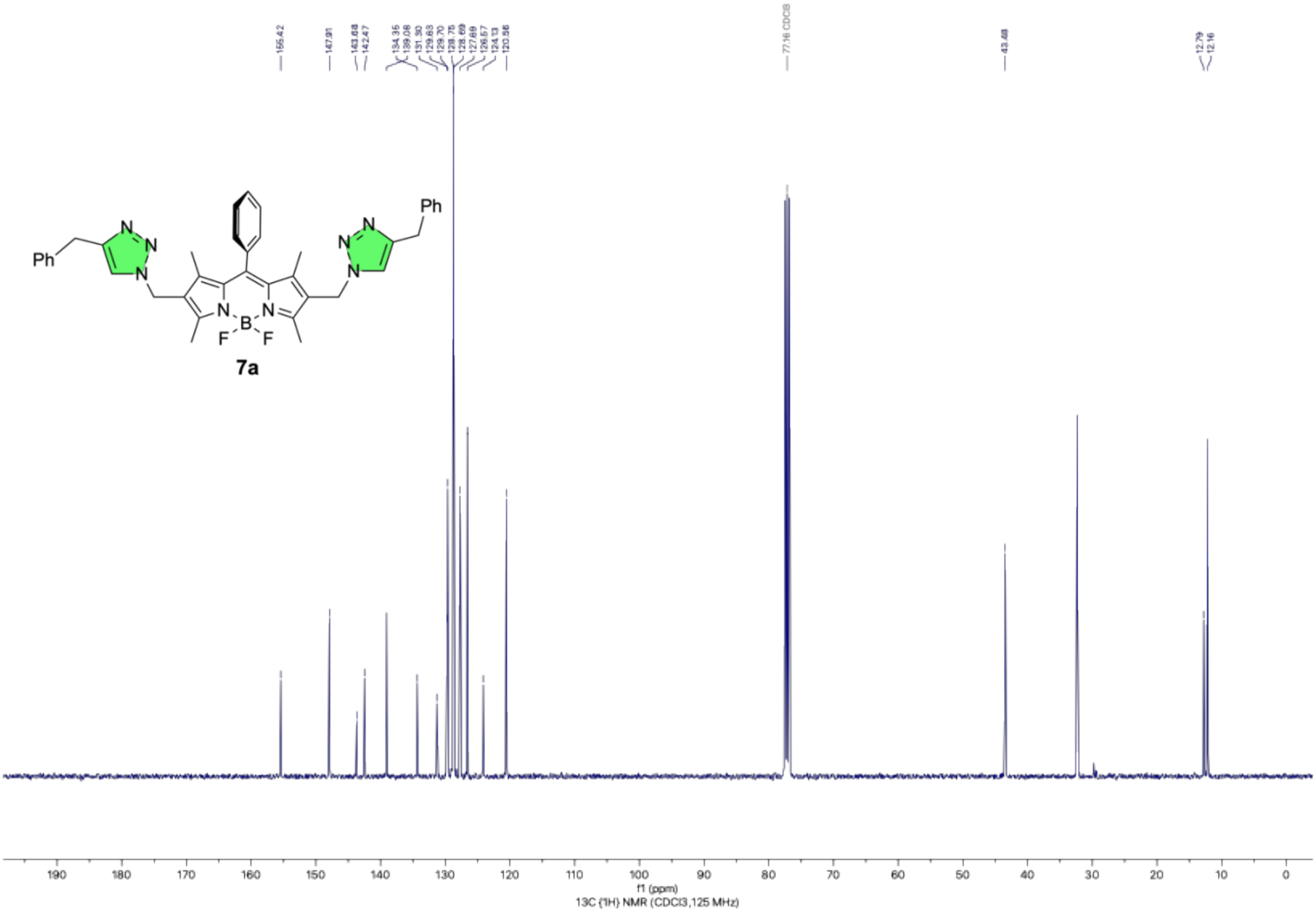

^{13}C {^{1}H}-NMR ($CDCl_3$, 125 MHz) of Compound **7a**

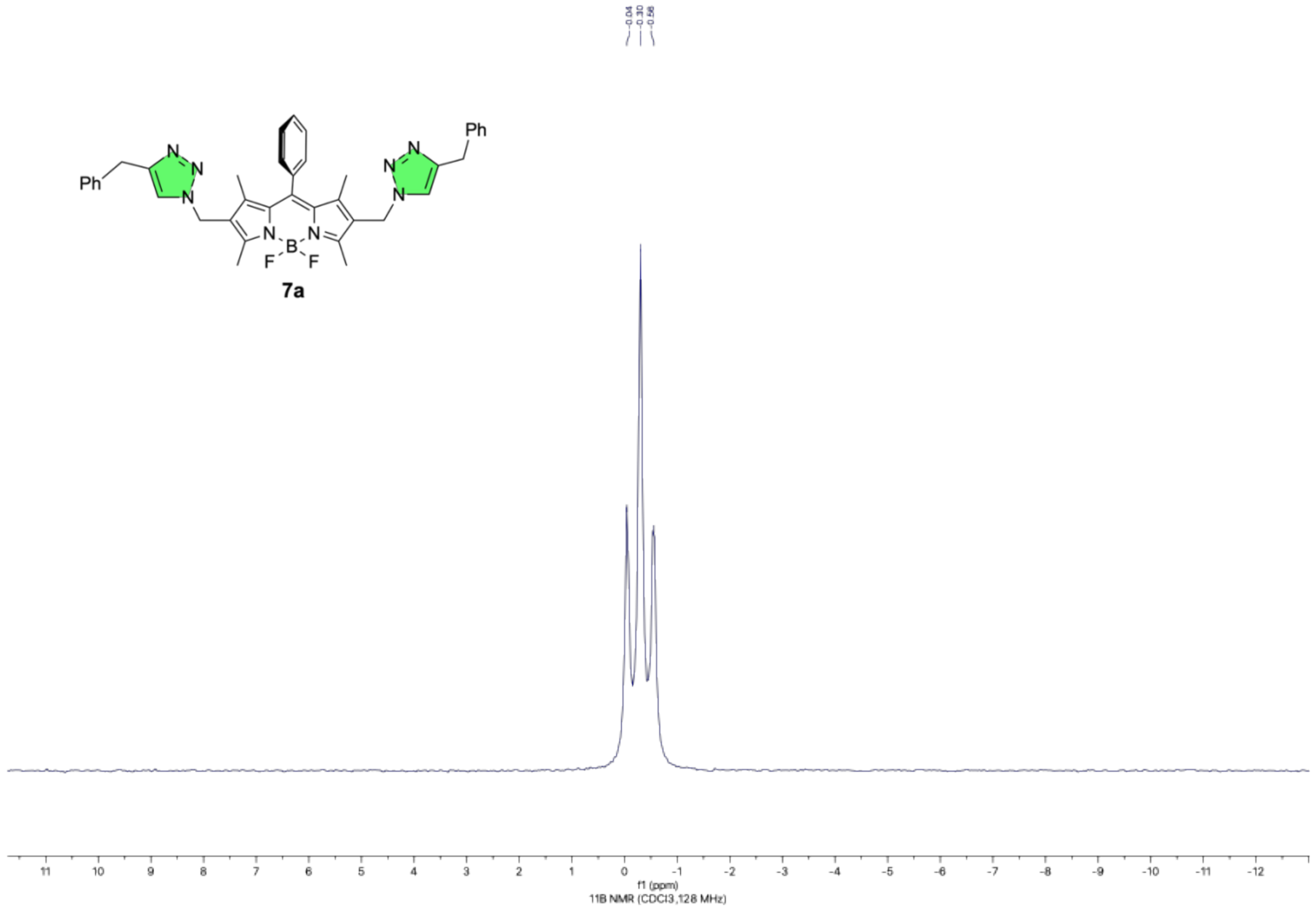

^{11}B-NMR ($CDCl_3$, 128 MHz) of Compound **7a**

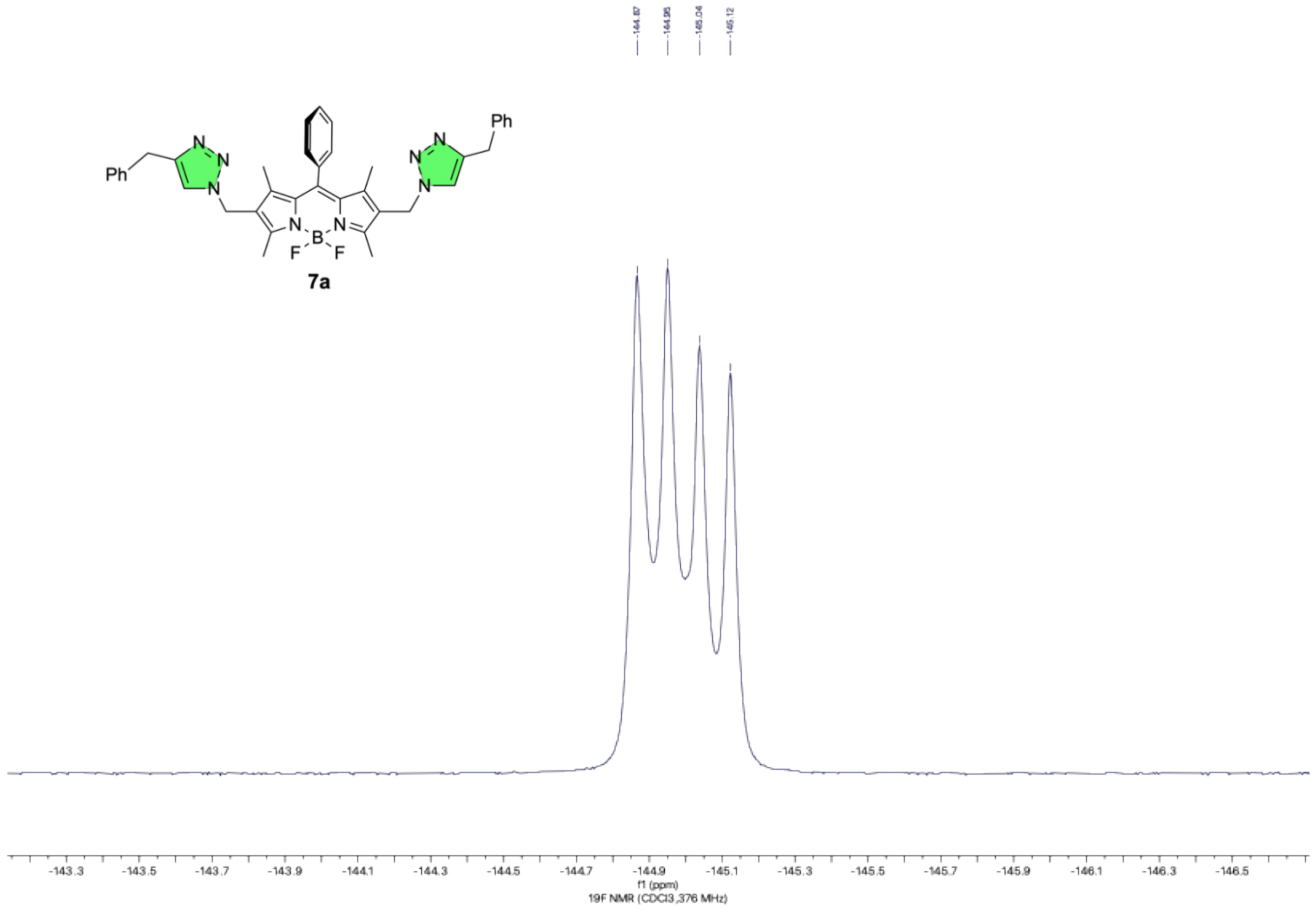

^{19}F-NMR ($CDCl_3$, 376 MHz) of Compound **7a**

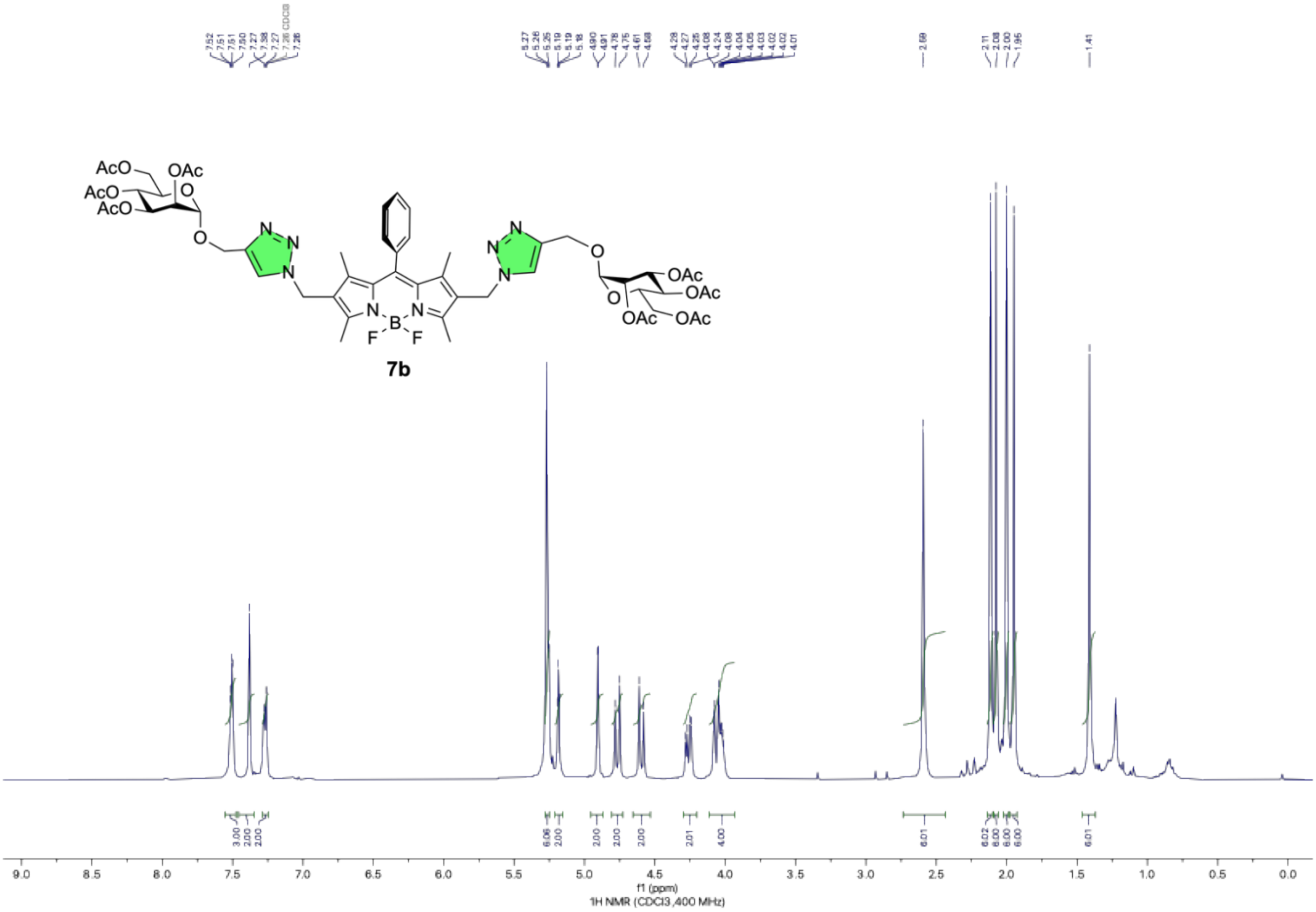

^{1}H-NMR ($CDCl_3$, 400 MHz) of Compound **7b**

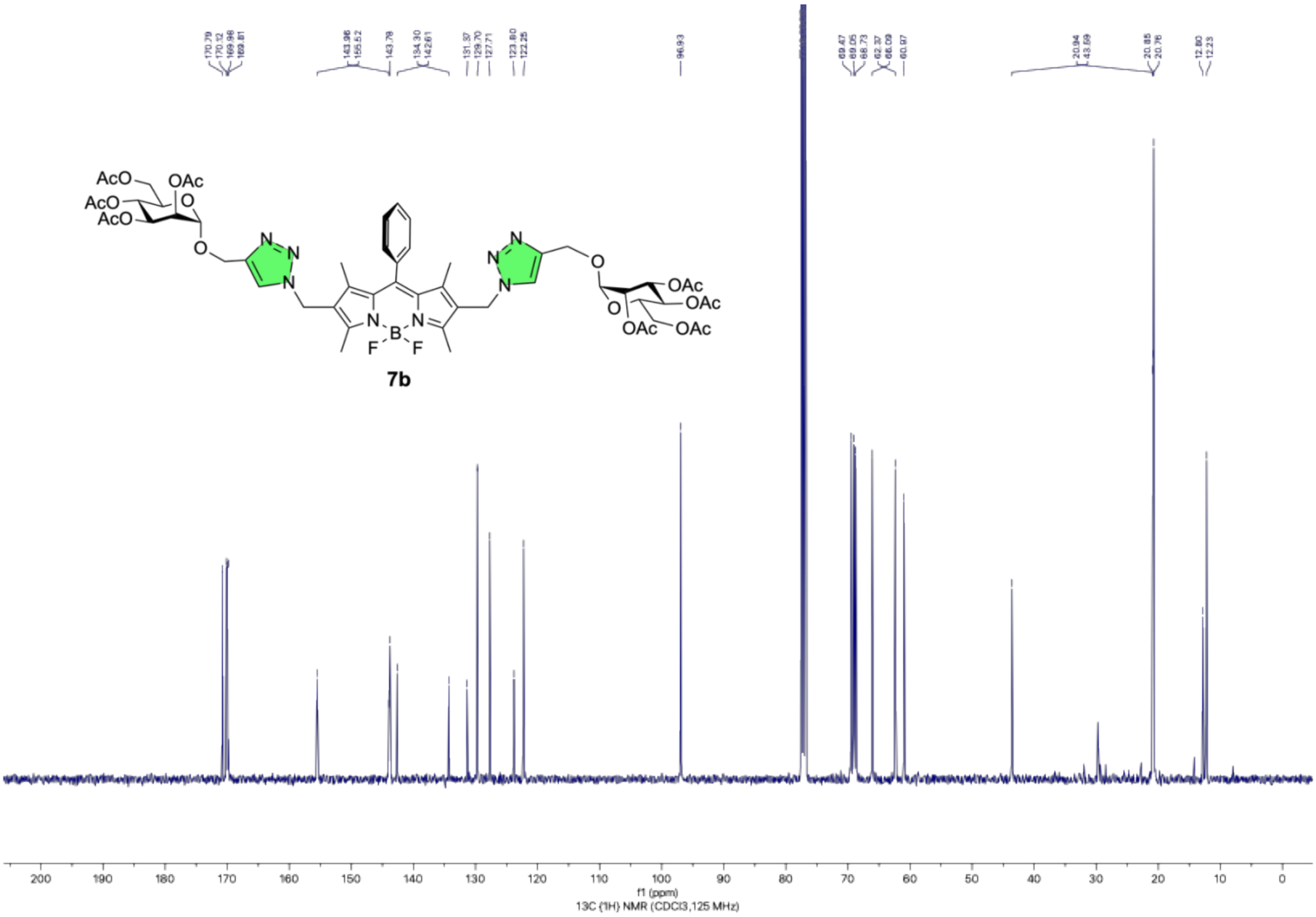

^{13}C {^{1}H}-NMR ($CDCl_3$, 125 MHz) of Compound **7b**

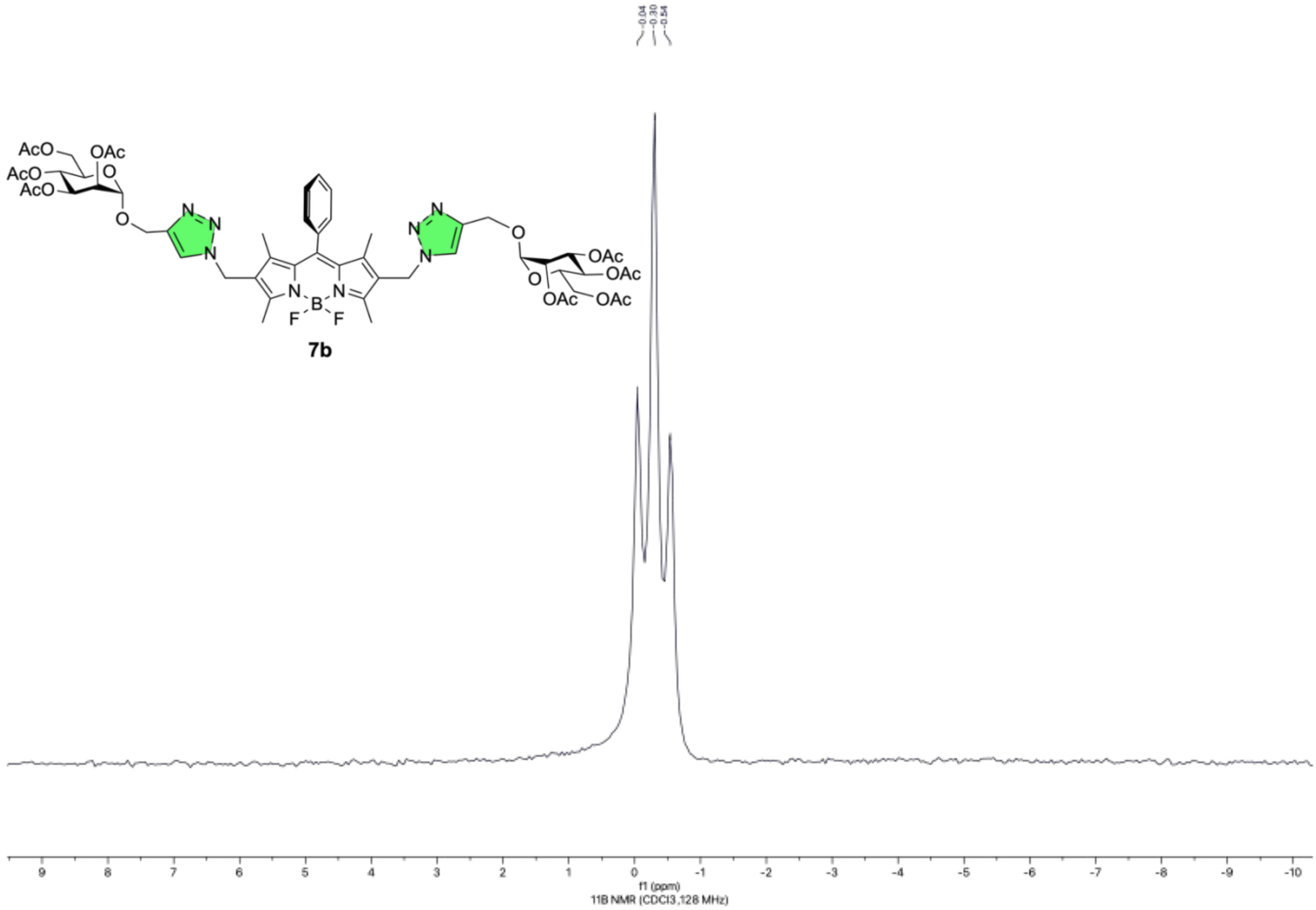

^{11}B-NMR ($CDCl_3$, 128 MHz) of Compound **7b**

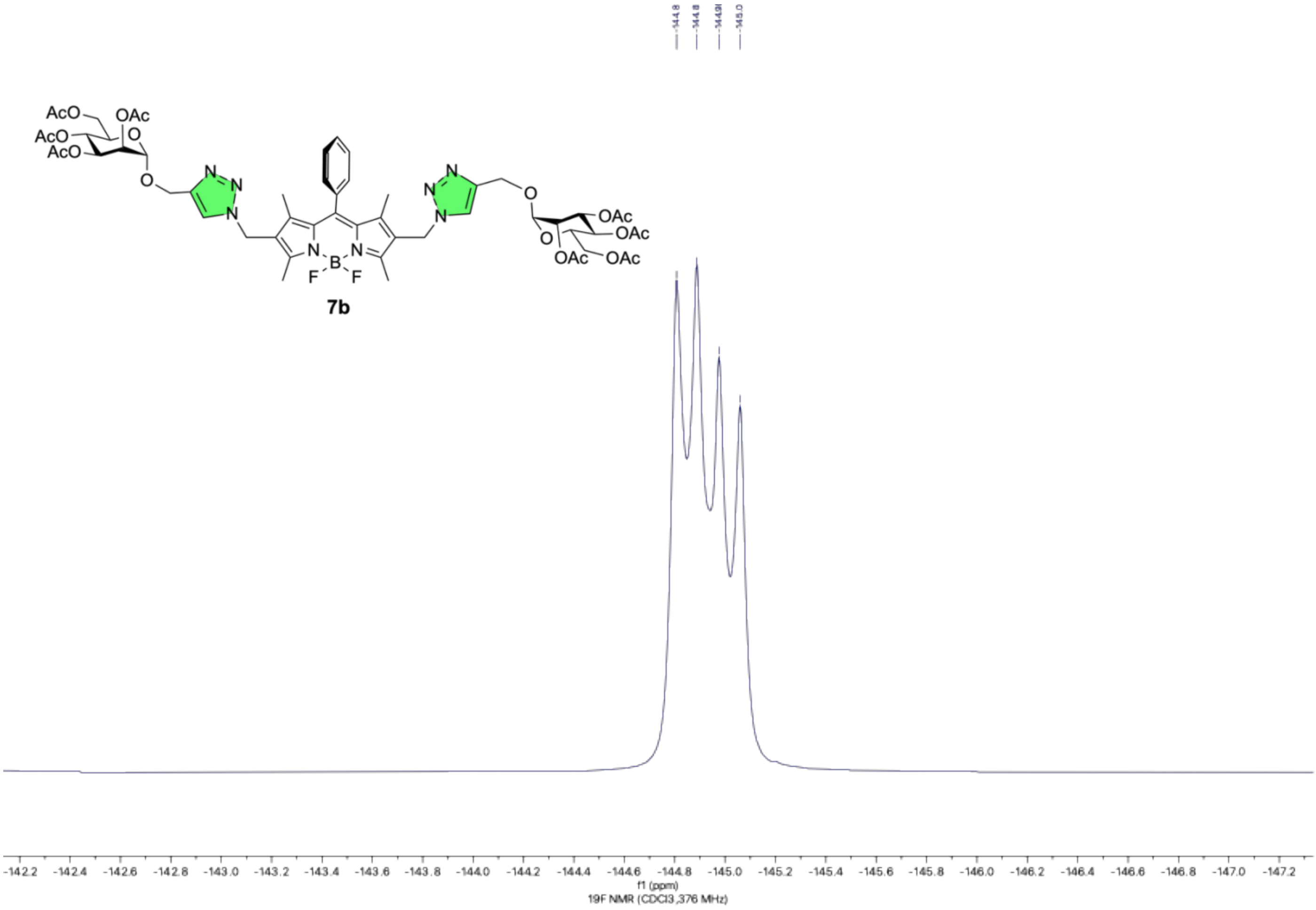

^{19}F-NMR ($CDCl_3$, 376 MHz) of Compound **7b**

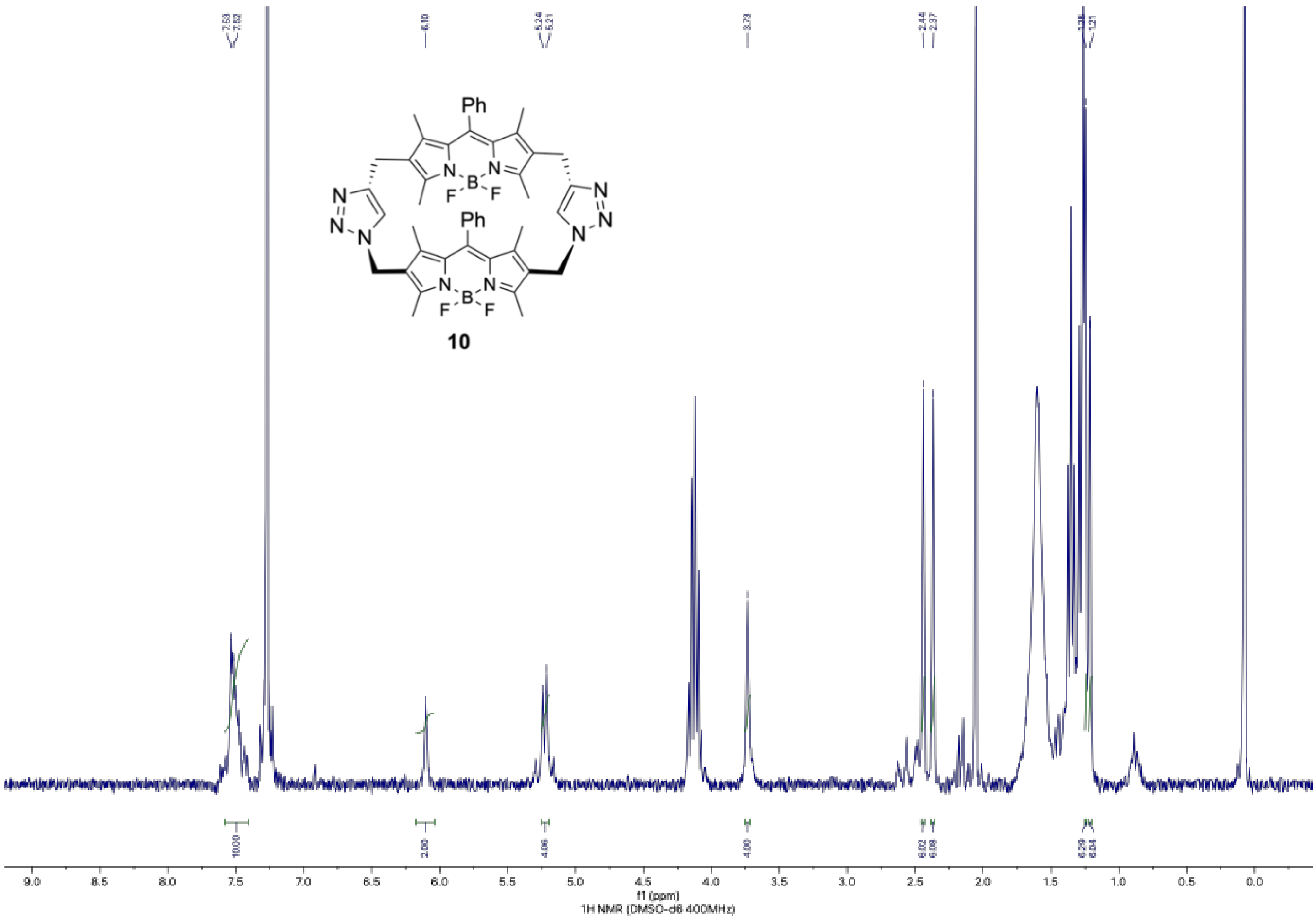

^{1}H-NMR (DMSO-d6/ $CDCl_3$, 400 MHz) of Compound **10**
